# Mini‐synplastomes for plastid genetic engineering

**DOI:** 10.1111/pbi.13717

**Published:** 2021-10-24

**Authors:** Alessandro Occhialini, Alexander C. Pfotenhauer, Li Li, Stacee A. Harbison, Andrew J. Lail, Jason N. Burris, Cristiano Piasecki, Agnieszka A. Piatek, Henry Daniell, C. Neal Stewart, Scott C. Lenaghan

**Affiliations:** ^1^ Department of Food Science University of Tennessee Knoxville TN USA; ^2^ Center for Agricultural Synthetic Biology University of Tennessee Institute of Agriculture Knoxville TN USA; ^3^ Department of Plant Sciences University of Tennessee Knoxville TN USA; ^4^ Department of Basic and Translational Sciences School of Dental Medicine University of Pennsylvania Philadelphia PA USA

**Keywords:** *Solanum tuberosum*, plastid engineering, plastome, small synthetic plastome ‘mini‐synplastome’, episomal replication, homologous recombination

## Abstract

In the age of synthetic biology, plastid engineering requires a nimble platform to introduce novel synthetic circuits in plants. While effective for integrating relatively small constructs into the plastome, plastid engineering via homologous recombination of transgenes is over 30 years old. Here we show the design–build–test of a novel synthetic genome structure that does not disturb the native plastome: the ‘mini‐synplastome’. The mini‐synplastome was inspired by dinoflagellate plastome organization, which is comprised of numerous minicircles residing in the plastid instead of a single organellar genome molecule. The first mini‐synplastome in plants was developed *in vitro* to meet the following criteria: (i) episomal replication in plastids; (ii) facile cloning; (iii) predictable transgene expression in plastids; (iv) non‐integration of vector sequences into the endogenous plastome; and (v) autonomous persistence in the plant over generations in the absence of exogenous selection pressure. Mini‐synplastomes are anticipated to revolutionize chloroplast biotechnology, enable facile marker‐free plastid engineering, and provide an unparalleled platform for one‐step metabolic engineering in plants.

## Introduction

With the emergence of plant synthetic biology, the need to secure the global food supply for a rapidly growing population, and the increased interest in biopharming, chloroplast engineering has once again emerged as a potential solution to meet these demands. For example, in the context of the current SARS‐CoV‐2 pandemic, angiotensin‐converting enzyme 2 expressed in lettuce chloroplasts has advanced to the clinic to treat COVID‐19 patients, after completion of toxicology and pharmacokinetic studies required for regulatory approval (Daniell *et al*., 2020). Therapeutic proteins expressed in edible plant cells have already been approved by FDA for both injectable and oral delivery (Fox, 2012; Vickery *et al*., 2018), paving the way for advancing protein drugs made in chloroplasts. In addition, chloroplast‐engineered molecules have been validated for developing oral booster vaccines to prolong immunity against infectious diseases, eliminating the need for cold storage and transportation (Chan *et al*., 2016; Daniell *et al*., 2019a; Xiao and Daniell, 2017). Further, chloroplast biotechnology offers very high production ceilings for heterologous proteins (up to 70% by total leaf protein) (Ruhlman *et al*., 2010), far surpassing the potential of traditional genetic engineering of the plant nuclear genome. Recent advances in chloroplast biotechnology have enhanced the ability of plastids to express large, complex eukaryotic and viral proteins through the development of new software and algorithms (Kwon *et al*., 2016, 2018). For more complex metabolic engineering, the prokaryotic transcription/translation machinery housed in plastids enables coordinated expression of multiple genes by a single promoter, simplifying the control of complex transgene cassettes (De Cosa *et al*., 2001; Kumar *et al*., 2012; Malhotra *et al*., 2016).

A hallmark of chloroplast biotechnology is the use of homologous recombination (HR) to insert transgenes, with a selection cassette, into the native plastome enabling the generation of stable transplastomic lines (Daniell *et al*., 1998; Kota *et al*., 1999; Lu *et al*., 2013; Svab *et al*., 1990; Svab and Maliga, 1993; Zhang *et al*., 2015). Marker free transplastomic lines have also been created in crops such as soybean (Dufourmantel *et al*., 2007) and lettuce (Daniell *et al*., 2019b, 2020; Kumari *et al*., 2019; Park *et al*., 2020). Currently, there is no alternative to HR for chloroplast genome engineering. Episomally replicating plasmids may provide an alternative engineering platform that leaves the native plastome untouched, with no foreign DNA integrated into native molecules. Indeed, the very first report of expressing foreign genes in chloroplasts demonstrated the value of autonomously replicating chloroplast vectors containing a fully characterized chloroplast origin of replication D‐loops (*ori*) (Daniell *et al*., 1990; Meeker *et al*., 1988; Nielsen and Tewari, 1988). Chloroplast vectors containing the *ori* showed prolonged expression in bombarded cells when compared to vectors without the chloroplast *ori* (Daniell *et al*., 1990). Although prolonged expression of foreign genes was observed in chloroplast vectors containing a chloroplast *ori*, this was a short‐term study. This study was followed by subsequent reports of the presence of an 868 bp *Nicotiana* plastid extrachromosomal element, NICE1, as an unexpected product of HR (Staub and Maliga, 1994, 1995). While the mechanism of replication of NICE1‐containing plasmids still remains unclear (Mühlbauer *et al*., 2002; Staub and Maliga, 1994), the NICE1 sequence was devoid of a chloroplast *ori* sequence. Shuttle vectors utilizing NICE1 were developed and proven capable of replication in *Escherichia coli* and able to generate transplastomic tobacco through expression of *aadA* on the episomal construct (Staub and Maliga, 1994, 1995). Other chloroplast transformation constructs, with promoter and terminator sequences homologous to host plastid DNA, have also been shown to generate episomal plasmids that persist at least to the T3 generation (Min *et al*., 2015b). As an alternative approach, a previous attempt was made to develop an episomal plastid transformation system in tobacco that was bioinspired by the dinoflagellate plastome organization (Min *et al*., 2015a). While the angiosperm plastome is composed of a single circular molecule ranging in size from 107 to 218 kb (Daniell *et al*., 2016; Shinozaki *et al*., 1986), in some marine dinoflagellates the plastome is significantly reduced and composed of multiple small minicircles of 2–3 kb (Howe *et al*., 2003, 2008; Koumandou and Howe, 2007). The core regions of each minicircle contain an *ori* and, in most cases, a coding region comprised of a single gene (Barbrook *et al*., 2012; Barbrook and Howe, 2000; Koumandou and Howe, 2007). Using a dinoflagellate *ori*, it was possible to engineer transplastomic tobacco with extrachromosomal circular DNA, but after relaxing the selective pressure, the plants rapidly lost the episome. Additionally, the recovery of GFP‐positive plants without the extrachromosomal DNA indicated that integration into the native plastome was occurring, preventing achievement of the ultimate goal (Min *et al*., 2015a).

The objective of this work was to develop a minicircle architecture, herein termed the mini‐synplastome, with the ability to express transgenes for selection and visualization without the need for integration of heterologous sequence into the native plastome. Further, the mini‐synplastome must persist for multiple generations without selection. In this way, mini‐synplastomes differ from spontaneous minicircles in that the architecture is stable and meets the goals for chloroplast biotechnology and synthetic biology. To validate the system, potato was chosen, as this is a relevant food crop that would significantly benefit from plastid engineering with regards to biofortification.

## Results and discussion

### Construction of Gen1 vectors with long‐homologous synthetic arms as a first step in the design–build–test cycle of the mini‐synplastome

**Figure 1:**
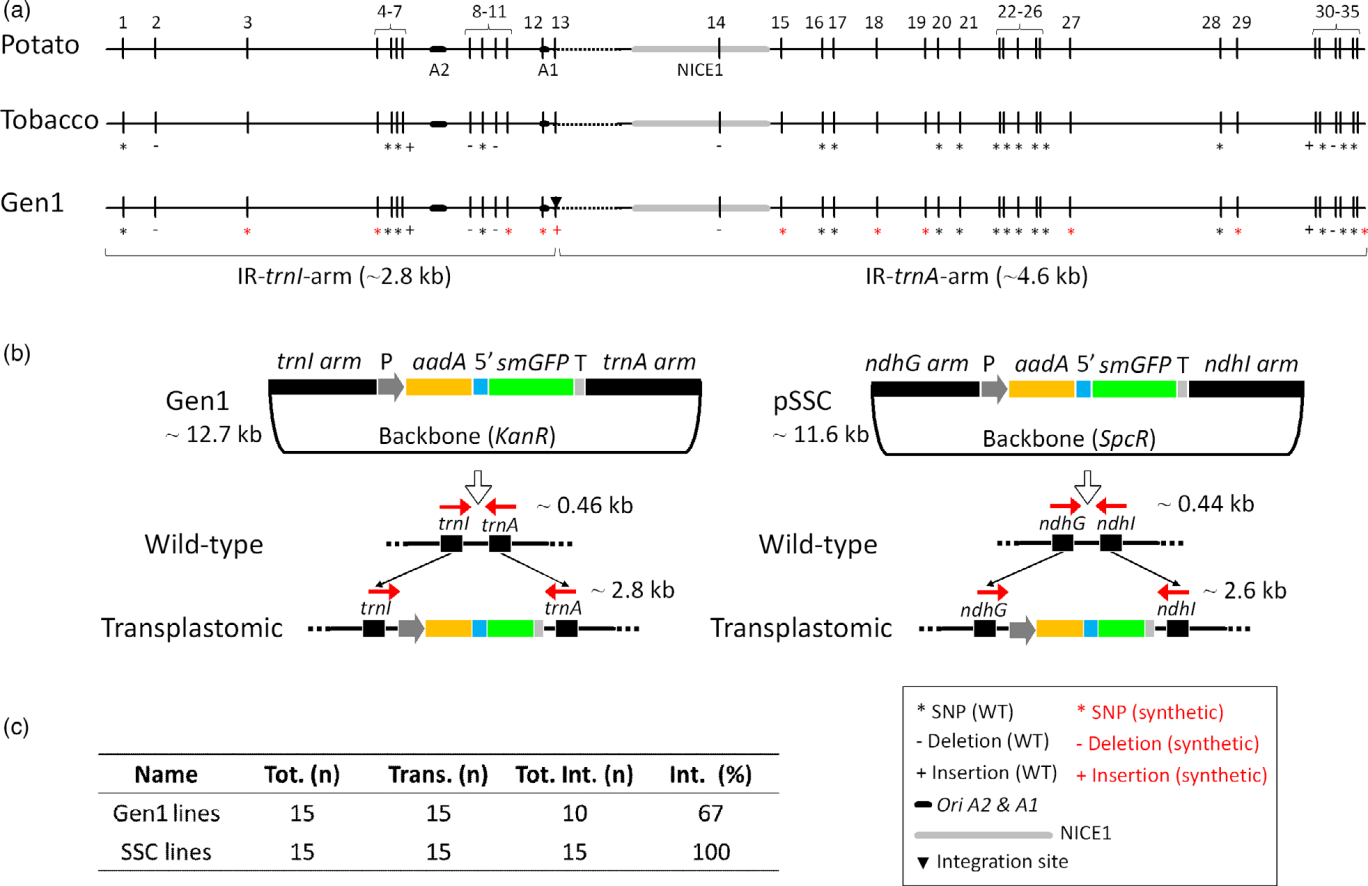
Vector design. (a) Design of *trnI*/*trnA* homologous arms (~2.8 and ~4.6 kb) used in Gen1 vector construction. An alignment of homologous regions in the potato and tobacco plastome along with the two synthetic sequences used in the design of the Gen1 vector are indicated. A total of 35 regions of sequence difference are located within homologous regions. A vertical bar (|) without any symbols indicates sequence identity compared to the potato plastome, whereas an asterisk (*), minus (−), or plus (+) indicates single nucleotide polymorphism (SNP), sequence deletion or addition, respectively. Black symbols indicate wild‐type mutations between potato and tobacco, whereas red symbols indicated synthetic mutations introduced in Gen1 sequences. *Ori*A2 and A1 (black bars) and NICE1 (grey bar) are also indicated. (b) Schematic representation of two DNA constructs, Gen1 (~12.7 kb) and pSSC (~11.6 kb), used in this work and their integration site in the native plastome. IR *trnI/trnA* homologous arms of the Gen1 vector (~2.8 and ~4.6 kb, respectively, including *oriA2*‐*A1* and NICE1) and *ndhG/ndhI* homologous arms of the pSSC (~1.8 and ~5 kb, respectively) are indicated. A selection cassette located between arms is indicated: *Prom‐SD* (P): *rrn* promoter along with a Shine‐Dalgarno sequence (grey); *aadA*: spectinomycin resistance gene (yellow); *5*′*UTR*: 5′ untranslated region (blue); *smGFP*: gene encoding the soluble monomeric green fluorescent protein (green); and *3*′*UTR* (T): 3′ untranslated region (light grey). Backbone vectors containing the kanamycin (*KanR*) or spectinomycin (*SpcR*) resistance gene are indicated in Gen1 and pSSC constructs. Gen1 and pSSC integration into *trnI/trnA* and *ndhG/ndhI* sites of the native plastome along with the location of primers used to check integration (red arrows) and the molecular weight of wild‐type (~0.46 and ~0.44 kb, for *trnI/trnA* and *ndhG/ndhI*, respectively) and transplastomic (~2.8 and ~2.6 kb, for Gen1 and pSSC integrated, respectively) PCR products are indicated. (c) Table indicating the percentage of vector integration in transplastomic lines. Tot. (n): total number of lines analysed; Trans. (n): total number of positive lines for the presence of *aadA* and *smGFP* genes; Tot. int. (n): total number of lines with vector integration; Int. (%): percentage of plants with vector integration.

To understand the necessary architecture for development of a mini‐synplastome for potato plastid engineering, a plastid engineering vector was designed with homology to the most common transgene integration site of tobacco, *trnI*/*trnA* (Daniell *et al*., 2016). In order to encompass the most likely components for episomal plasmid replication from tobacco (NICE1 and *ori A2* and *A1*) (Krishnan and Rao, 2009; Staub and Maliga, 1994), long homology arms (~2.8 and ~4.6 kb) were utilized (Figure 1a). The use of tobacco homology arms for engineering potato plastids was intentional with the goal to prevent direct sequence homology to enable tracking of recombination. It is known that intergenic spacer regions among *Solanaceae* chloroplast genomes are not highly conserved (Daniell *et al*., 2006). It should also be noted that the homologous NICE1 sequence in potato contains an insertion of 102 bp not found in the NICE1 sequence from tobacco, as indicated in Figure 1a (position 14, Figure 1a; Table S1). Further, a series of single nucleotide polymorphisms (SNPs) and a multi‐cloning site were introduced into the tobacco homology arms to provide more markers to identify common sites of recombination and enable alternative cloning strategies (Figure 1a). In total, the synthetic generation 1 (Gen1) vector contained ~7.4 kb of native tobacco plastid sequence flanking the *trnI*/*trnA* integration site with 35 separate SNP and indel mutations (Figure 1a; Table S1). For selection of engineered lines, a common dual selection (*aadA* and *smGFP*) cassette (Kwon *et al*., 2013) was cloned into the *trnI/trnA* site in Gen1. As a control for HR‐mediated chloroplast biotechnology, a vector for potato plastid transformation with homology arms of ~1.8 and ~5 kb targeting the *ndhG/ndhI* integration site within the small single copy region (SSC), and the same dual selection cassette was used (Occhialini *et al*., 2020). It should be noted that the Gen1 construct was designed to recombine with the native plastome along the ~7.4 kb homology arms and integrate the transgene cassette into the commonly used *trnI*/*trnA* integration site on the native plastome. However, we hypothesized that persistence of episomal DNA would be possible due to the selective pressure within the compartment (plastid). This was anticipated to reach a steady state in the plastid if there was constant recombination back‐and‐forth between an episomal plasmid and the native plastome.

### Screening for transgenic lines harbouring plastid‐replicating episomal units

To test our hypothesis, the Gen1 construct was transformed into potato leaf discs using biolistics and selected on spectinomycin. Selection yielded numerous transplastomic shoots. Molecular analysis confirmed that with the Gen1 construct 67% of the transplastomic shoots had the transgene cassette integrated into the *trnI*/*trnA* integration site of the native plastome (Figure 1b,c). Of the remaining shoots, the *KanR* gene could be amplified by PCR in 2 of the 5 Gen1‐containing plants hinting at the presence of an episomally replicating plasmid (Figure S1). The three remaining lines were hypothesized to result from recombination into an undetected site or represented escapes from selection. For the control vector, pSSC, the transgene cassette was correctly integrated into the *ndhG/ndhI* integration site in all transplastomic lines with no episomal plasmids detected (Figure 1b,c; Figure S1). In all transplastomic lines obtained, plants grew vigorously *in vitro* and high GFP fluorescence was observed in chloroplasts (Figure S2).

**Figure 2:**
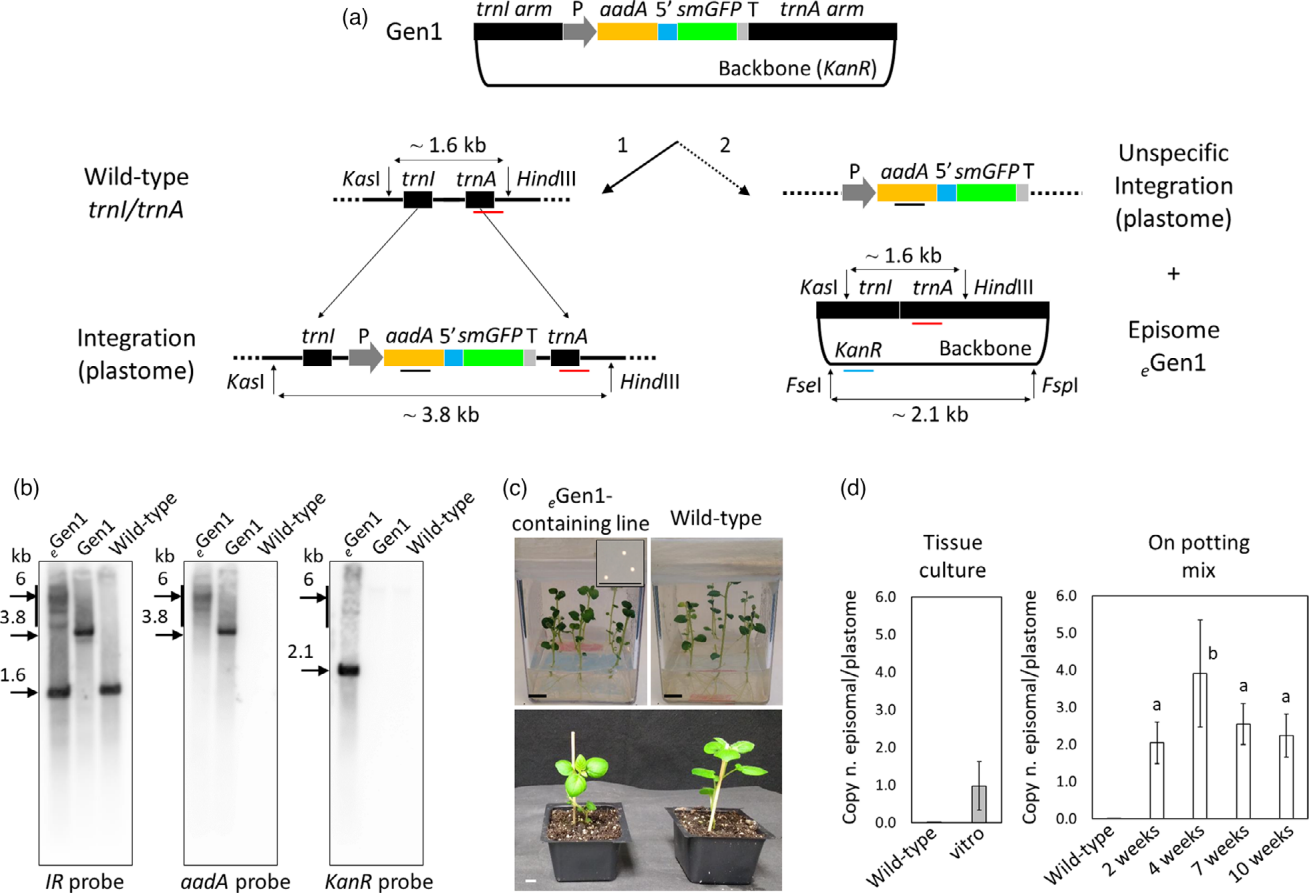
Characterization of *_e_* Gen1‐containing lines. (a) Schematic representation of the possible fates of the Gen1 vector in plants are shown: (1) integration of the transgene cassette into the *trnI/trnA* site of the potato plastome; (2) nonspecific integration of the transgene cassette into an unknown site, while an episome (*_e_* Gen1) is formed from the vector backbone and the remaining homologous sequence. The *rrn* promoter and a Shine‐Dalgarno sequence (P; grey); *aadA* gene (yellow); 5′ untranslated region (5′; blue); *smGFP* gene (green); 3′ untranslated region (T; light grey); the *trnI/trnA* arms; and the backbone vectors containing *KanR* gene are indicated in the figure. The location of *IR*, *aadA*, and *KanR* probes is indicated with red, dark, and blue bars, respectively. The molecular weights of DNA fragments obtained using *KasI*/*Hind*III (for *IR* and *aadA* probes) and *Fse*I/*Fsp*I (for the *KanR* probe) restriction enzymes are also indicated. (b) Southern blot analysis performed using an *IR*, *aadA* or a *KanR* probe and total leaf genomic‐DNA preparations from an *_e_* Gen1‐containing line, a Gen1‐integrating line, and a wild‐type control is shown. Molecular weight of DNA fragments (kb) are indicated in the blots. (c) Wild‐type potato plants along with the *_e_* Gen1‐containing line grown *in vitro* and for 2 weeks in potting mix are shown. Insert shows bacterial colonies transformed with *_e_* Gen1 extracted from leaf tissue (scale bar: 10 mm). (d) Graph summarizing the ratio of copy number of episomal DNA vs the copy number of plastome (copy n. episomal/plastome) in genomic DNA preparations of *_e_* Gen1‐containing plants by qPCR. Wild‐type potato plants were used as negative control. Two graphs representing *in vitro* plants at the second round of tissue culture and plants grown on potting mix without selection (2, 4, 7, and 10 weeks) are shown. Results are expressed as mean ± standard deviation. For plants in tissue culture, 5 biological and 9 technical replicates per biological replicate were used. For plants on potting mix, 3 biological and 4 technical replicates per each biological replicate were used. Means separation was evaluated using ANOVA Tukey HSD (*P* < 0.05). Statistical significance is indicated by different letters.

To confirm that Gen1‐containing plants with undetectable transgene integration and amplification of the *KanR* gene contained the episomally replicating Gen1 backbone, total leaf genomic DNA was analysed by Southern blot and PCR (Figure 2 and Figure S3, respectively). Southern blots performed using two different probes (IR and *aadA*) confirmed no detectable transgene integration into the *trnI/trnA* site of the native plastome in Gen1 episome‐containing plants, as compared to plants with the transgenes from Gen1 integrated into the native plastome (~3.8 kb bands in both *IR* and *aadA* DNA blots). Southern blots using a *KanR* probe similarly displayed the full‐length backbone in the Gen1 episome‐containing plants (Figure 2a,b).

### Characterization of the first generation of episomal units (*_e_* Gen1) extracted‐back from leaf tissue

Episomes contained in chloroplasts of selected transgenic lines were isolated by back‐transformation in *E. coli*. Unsurprisingly, after bacteria transformation using total genomic DNA isolated from plants containing the episomal Gen1 backbone, colonies were recovered that contained plasmids (Figure 2c), indicating that the Gen1 episomal backbone persisted in plastids as replicating extraplastomic DNA. PCR and sequence analysis of entire plasmids extracted from *E. coli* demonstrated the presence of the Gen1 backbone along with full‐length homologous arms devoid of the transgene cassettes (Figure S4). Since earlier molecular analysis (Figure 2; Figure S3) confirmed that the transgene cassette was not present in the expected integration site, it was hypothesized that the transgene cassette recombined in an undetermined location, which is not uncommon in chloroplast engineering (Gray *et al*., 2009) (Figure 2a). The presence of identical profiles of multiple DNA bands at higher molecular weight (>3.8 kb, with a main ~6 kb band) obtained with both *IR* and *aadA* probes further supports this hypothesis (Figure 2b).

Using the sequence variability described earlier, it was possible to track recombination events within the homology arms of Gen1 by comparing the sequence of the initial Gen1 construct with the sequence of the plasmid isolated from whole plant genomic DNA of stable transplastomics (*_e_* Gen1). Sequence analysis of *_e_* Gen1 demonstrated numerous recombination events between the homology arms and the endogenous plastome (Table S1). The homologous arms of *_e_* Gen1 recombined with the native potato plastome replacing 17 of the 35 mutations present in the bombarded version of the vector (M2, M10, M12‐14, M18‐29) (Table S1). Based on the pattern of mutations that were converted from the synthetic version of the homology arms to potato‐specific sequences, it was hypothesized that there were four recombination events. Unlike standard chloroplast biotechnology, in this instance we were concerned with HR of native plastome sequence into the episomal construct, as opposed to HR from a vector into and between the native plastome. In previous work using transformation vectors with homology arms from distinct species, 100% correction of the heterologous sequences was observed (Ruhlman *et al*., 2010).

### The *_e_* Gen1 episome is maintained as extraplastomic DNA throughout multiple plant developmental stages

Despite only ~50% conversion of the homology arms of *_e_* Gen1 to potato‐specific sequence, *_e_* Gen1 was the only plasmid recovered during all 3 rounds of tissue culture and isolated from plants grown on potting mix including tubers. Since the sequence of *_e_* Gen1 was invariant across multiple generations, we hypothesized that the sequence of *_e_* Gen1 was imposing limits to recombination across the other heterologous sites. Further, no mutations were detected in the backbone of the episomal plasmid, suggesting that the backbone was devoid of significant homology with the native plastome. The lack of mutations within the backbone was crucial to understanding how to better design episomally replicating vectors without integration of foreign DNA. Surprisingly, the average copy number of *_e_* Gen1 was ~1 copy per endogenous plastome in plants maintained in tissue culture (Figure 2d) and persisted in 3 successive tissue culture generations (Figure S5). In addition, *_e_* Gen1 could be isolated from all life cycle stages through anthesis on plants grown in pots without selective pressure (Figure 2d; Figure S6). Over time, the average copy number of *_e_* Gen1 per plastome shifted from 2.04 ± 0.56 at 2 weeks to 3.91 ± 1.44 at 4 weeks in pots before stabilizing at ~2.5 copies per plastome throughout the remaining life cycle of the plant (Figure 2d). In fact, in plants grown from tubers harvested from *_e_* Gen1‐containing plants, *_e_* Gen1 could still be recovered with no changes to the sequence (Figure S7). While plants were not sexually reproduced, it is anticipated, based on earlier work with NICE1 in tobacco, that *_e_* Gen1 would be present at minimal to no copy number in potato seed (Staub and Maliga, 1994). While researchers have demonstrated that highly inefficient paternal transmission of plastid DNA is possible (Ruf *et al*., 2007; Svab and Maliga, 2007), it is generally accepted that plastid transformation is an effective method of bioconfinement of GM crops. Based on these findings, we speculate that mini‐synplastome technology would provide a similar high degree of bioconfinement.

### Design of the mini‐synplastome (Gen2) for plastid genetic engineering

**Figure 3:**
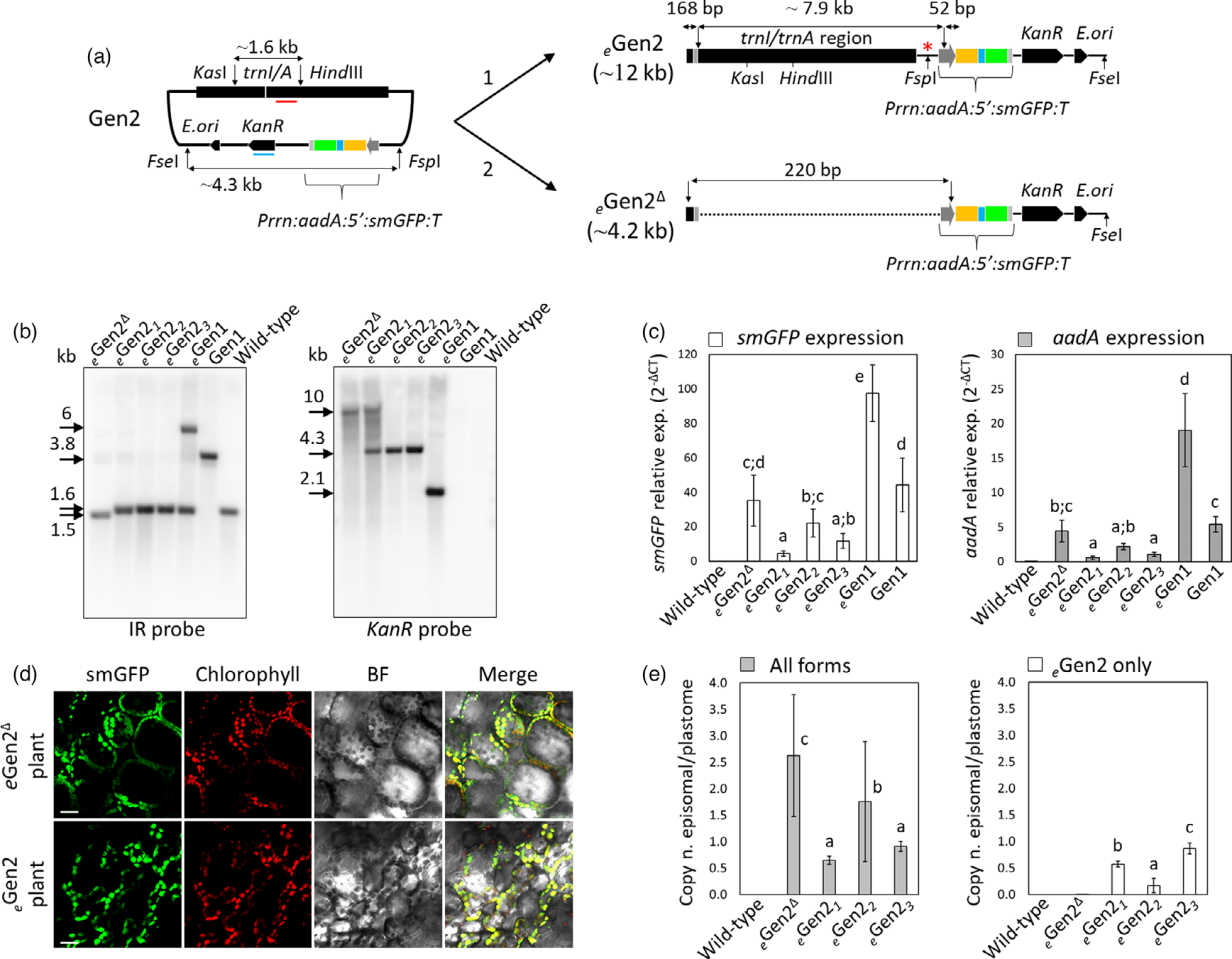
Characterization of synplastomic *_e_* Gen2‐containing lines. (a) Schematic representation of the possible fates of the Gen2 vector in plants are shown: 1) full‐length *_e_* Gen2 and/or *_e_* Gen2^Δ^. For simplicity of sequence comparison, these two plasmids, *_e_* Gen2 and *_e_* Gen2^Δ^, are represented in linear form. In *_e_* Gen2^Δ^, ~7.9 kb was excised by loop‐out recombination reconstituting *Prrn*. Perpendicular black arrows indicate deletion sites, while parallel double arrows indicate molecular weight of fragments. The *trnI/trnA* homologous region and the backbone vector containing the *KanR* gene, an *E. coli* origin of replication (*E*. *ori*), and the dual selection cassette are indicated. The *Prrn* promoter fused to a ribosome binding site (P, deep grey); *aadA* gene (yellow); 5′UTR (5′, blue); *smGFP* gene (green); and the 3′UTR/terminator (T, grey) are indicated in the selection cassette. Restriction enzyme combinations used for Southern blots, *Kas*I/*Hind*III and *Fse*I/*Fsp*I, together with the sizes of predicted DNA fragments are indicated. The *Kas*I/*Hind*III and *Fse*I/*Fsp*I fragments were detected by a ~0.5 kb probe designed on *trnI/trnA* (red bar) or *KanR* (blue bar), respectively. (b) Southern blot analysis performed using either an *IR* or *KanR* probe and leaf total DNA preparations from *_e_* Gen2‐containing lines 1–3 and a line harbouring *_e_* Gen2^Δ^. DNA samples from *_e_* Gen1‐containing and Gen1‐integrating lines along with wild‐type plants were used as a comparison. Molecular weight of DNA fragments (kb) are indicated in the blots. (c) qRT‐PCRs using cDNA preparations from *_e_* Gen2‐containing lines 1–3 and *_e_* Gen2^Δ^ lines. *_e_* Gen1‐containing and Gen1‐integrating lines along with wild‐type controls have been included. Graphs showing the relative expression of *smGFP* (white bars) and *aadA* (grey bars) compared to the internal reference gene *ef1* (*y* axis: $2^{‐\Delta C_{T}}$) are shown. The results are expressed as mean ± SD (standard deviation) of three biological and three technical replicates. (d) Confocal image showing smGFP localization to the chloroplast in both *_e_* Gen2‐ and *_e_* Gen2^Δ^‐containing lines. smGFP (green), chlorophyll (red), bright field (BF), and merged images are shown. Scale bars: 20 µm. (e) Graph summarizing the ratio of episomal plasmid copy number to the copy number of the plastome (copy n. episomal/plastome) in genomic DNA preparations of *_e_* Gen2*‐* and *_e_* Gen2^Δ^‐containing lines determined by qPCR. Wild‐type plants were used as a negative control. PCR analyses were performed to calculate the ratio of copy number for all forms (*_e_* Gen2 and *_e_* Gen2^Δ^; grey bars) and only the full‐length *_e_* Gen2 (white bars). Results are expressed as mean ± standard deviation of five biological and three technical replicates per each biological replicate. In graphs C and E, mean separation was evaluated using ANOVA Tukey HSD (*P* < 0.05), and statistical significance is indicated by different letters.

As discussed previously, sequence analysis of *_e_* Gen1 recovered after subsequent rounds of tissue culture (1–3) and at different plant developmental stages in potted plants indicated no variability in sequence, confirming a stabilized episomal architecture. Thus, the plants from this first design–build–test cycle taught us how to design the mini‐synplastome. However, to achieve the ultimate goal of chloroplast engineering without the integration of foreign DNA, we modified *_e_* Gen1 to express transgenes directly from the episomal construct by inserting the dual selection cassette flanked by the vector backbone instead of the homology arms. This modification led to the design and assembly of the Gen2 version of the construct (Figure 3). It should be noted that the sequence for the ~7.4 kb homology arms flanking the *trnI/trnA* integration site present in Gen1 remained in Gen2, although the transgene cassettes were no longer inserted into the integration site, as described above (Figure 3a). In shuttle vectors previously engineered using NICE1 (Staub and Maliga, 1994) and multiple dinoflagellate *ori* (Min *et al*., 2015a), transgene integration with the endogenous plastome was observed; thus, it was necessary to eliminate these recombination events to meet the requirement of the mini‐synplastome engineering platform. After assembly, Gen2 was transformed into potato leaf discs by biolistics and selected against spectinomycin. In this round, however, the spectinomycin resistance gene, *aadA*, was expressed from the episomal construct, not from a transformed plastome. The spectinomycin‐resistant shoots harboured no cassette integration into the plastome, as determined by Southern blot analysis; however, plants were positive for Gen2 (Figure 3b; Figure S8).

### Synplastomic lines harbour the *_e_* Gen2 plasmid as non‐integrating episomal unit with intact transgene backbone structure

The back‐transformation to *E. coli* of total leaf genomic DNA preparations from Gen2 lines followed by sequence analysis of the entire plasmid recovered (*_e_* Gen2) confirmed the presence of an episomally replicating construct (Figure S9; Table S1). Compared with *_e_* Gen1, the homologous arms of *_e_* Gen2 were comprised entirely of potato‐specific sequences with the exception of M1 and M35 (Table S1). Since M1 and M35 were at the end of the homology arms, the lack of correction of these mutations was expected since the flanking sequence on one side of the mutations was 98 and 7 bp, respectively. In fact, M1 and M35 were the only mutations that were not corrected in both *_e_* Gen1 and *_e_* Gen2. The correction of all other mutations in *_e_* Gen2, as opposed to *_e_* Gen1, indicated that insertion of the transgene cassettes in between the homology arms causes disruption of HR leading to the lack of near complete correction in *_e_* Gen1. Considering that nearly total conversion of the homology arms in *_e_* Gen2 to the native plastome sequence took multiple cycles, it is likely that the initial design of heterologous sequence in Gen1 allowed the resulting episomal constructs to persist in plastids longer than if there was near complete homology.

Whole plasmids recovered from *E. coli* revealed two distinct versions of *_e_* Gen2, full‐length *_e_* Gen2 and *_e_* Gen2^Δ^ (Figure 3a). Full‐length *_e_* Gen2 had complete homology arms, whereas *_e_* Gen2^Δ^ had a recombination event between the *Prrn* promoter sequence on the homology arm with the *Prrn* promoter on the transgene cassette, eliminating ~7.9 kb of sequence (Figure 3a). Sequence similarity between the *Prrn* promoter on the homology arm and transgene cassette were known; however, the homologous flanking regions at the reconstitution sites were only 168 and 33 bp with the total recombination event reconstituting a mere 220 bp of sequence (Figure 3a). Conventional tobacco chloroplast engineering typically uses homologous arms of ~1 kb (much longer for lettuce) (Daniell *et al*., 2019b; Kumari *et al*., 2019; Kwon *et al*., 2016, 2018; Meeker *et al*., 1988; Park *et al*., 2020; Ruhlman *et al*., 2010) although the use of smaller direct repeats of up to 174 bp in length has been used for direct‐repeat mediated excision of marker genes (Iamtham and Day, 2000). In terms of efficiency, even with the goal of introduction of a single SNP into the native plastome, homology arms of ~ 600 bp are recommended (Martin Avila *et al*., 2016). Similarly, as indicated in the generation of NICE1, imperfect direct repeats as small as 16 bp can facilitate effective recombination in plastids (Staub and Maliga, 1994). Evolutionary studies of plastid genomes have revealed similar small inversions indicating some mechanism for recombination of 10–20 bp direct or imperfect direct repeats (Day and Madesis, 2007). Thus, while not unprecedented, the efficiency of this within episome recombination was unexpected.

Southern blots performed on *_e_* Gen2‐containing plants at the first and second vegetative generations of synplastomic lines displayed only the presence of the wild‐type *trnI/trnA* fragment (~1.6 kb bands, Figure 3b and Figure S10) confirming that there was no integration into the native plastome at this site. Sequence analysis performed on the sequence flanking the integration site revealed a truncated *trnI/trnA* fragment resulting from a ~100 bp deletion corresponding to M14 (Figure 1a) that was present in the endogenous plastome in a single *_e_* Gen2^Δ^ ‐containing plant (Table S2). As shown in Figure 2b, in a control *_e_* Gen1‐containing plant, a higher molecular weight band (~ 6 kb) associated with the wild‐type *trnI/trnA* fragment was observed (Figure 3b). As described previously, this fragment was likely the result of an unpredicted HR event between the transgene cassette and plastome, while the backbone of *_e_* Gen1 was still episomally maintained (Figure 2). A plant with integration of the transgene cassette from Gen1, along with wild‐type plants, was used as positive (~3.8 kb band) and negative (~1.6 kb band) controls for cassette integration into the *trnI/trnA* site of the native plastome (Figure 3b; Figure S10). Southern blots probed with *KanR* DNA revealed the presence of the full‐length backbone in *_e_* Gen2‐containing plant lines 1–3 (~4.3 kb Figure 3b and Figure S10). In *_e_* Gen2^Δ^‐containing plant lines and *_e_* Gen2‐containing line 1, a high molecular weight band (≥10 kb) was observed, which indicated remaining uncut *_e_* Gen2 (Figure 3b). In total, these data confirmed that *_e_* Gen2 persisted as a non‐integrating episomal plasmid over two rounds of and that the transgenes were effectively expressed from the episomes. Therefore, we concluded that *_e_* Gen2 was the first mini‐synplastome vector to be developed.

### The *_e_* Gen2 episome confers efficient transgene expression and it is present at high copy number throughout all plant developmental stages

RT‐qPCR performed using cDNA preparations from mini‐synplastomic lines indicated that *_e_* Gen2 conferred high *smGFP* and *aadA* expression in chloroplasts (*_e_* Gen2‐containing lines 1–3 and *_e_* Gen2^Δ^‐containing lines) (Figure 3c; Figure S11). However, compared to a homoplasmic plant line with integration of the Gen1 transgene cassette, *smGFP* expression was reduced by ~ 90%, 50% and 75% in the *_e_* Gen2‐containing lines 1–3. For *aadA* expression a reduction of ~85% to ~60% was observed in the same *_e_* Gen2‐containing lines 1–3. It should be noted that *smGFP* and *aadA* expression was under the control of the same promoter, but different 5′UTRs, leading to higher expression of *smGFP* relative to *aadA* in all lines. The level of transgene expression for *_e_* Gen2^Δ^‐containing lines was not significantly different from the control line with integration of the Gen1 transgene cassette. The Gen1 control line represents the typical HR strategy for chloroplast engineering and the expression of transgenes from *_e_* Gen2^Δ^‐containing lines achieved comparable expression to the current state of the art. Surprisingly, comparison of *smGFP* and *aadA* expression in a representative *_e_* Gen1‐containing line was 120% and 250% higher when compared to a plant line with integration of the Gen1 transgene cassette, but without persistent of the episome (Figure 3c). The extremely high‐level expression of the *_e_* Gen1‐containing line indicated that, for the purposes of heterologous protein production, there may be benefit in using a combined integrating/episomal strategy. The presence of plastid‐localized GFP signal was further confirmed through microscopic analysis of both *_e_* Gen2‐ and *_e_* Gen2^Δ^‐containing lines (Figure 3d).

**Figure 4:**
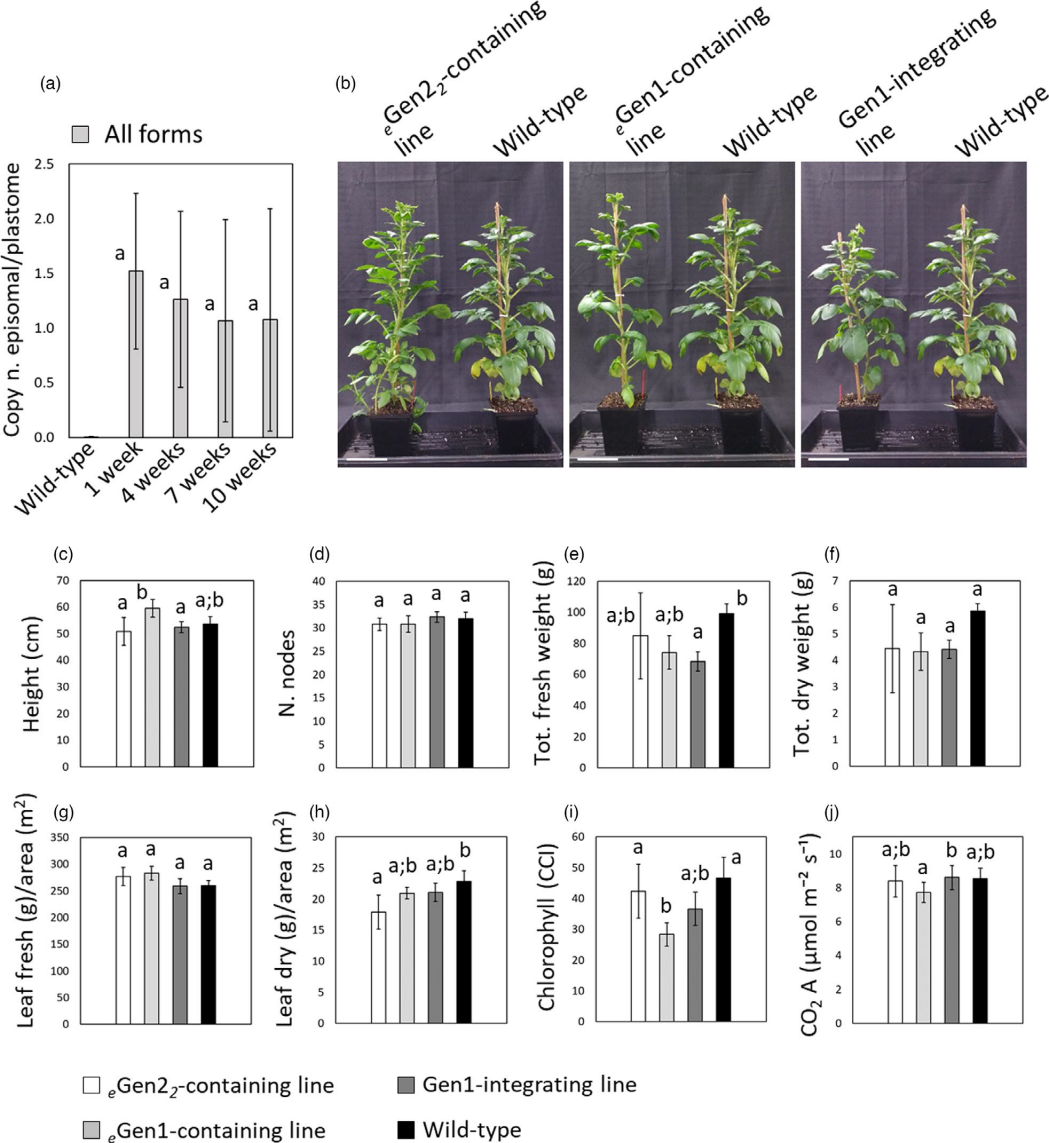
Growth characteristics of synplastomic plants. (a) Graph summarizing the ratio of episomal plasmid copy number vs the copy number of plastome (copy n. episomal/plastome) in genomic DNA preparations of *_e_* Gen2‐containing line 2 at different plant developmental stages (1, 4, 7, and 10 weeks in pots) determined by qPCR. Wild‐type plants were used as negative controls. PCRs were performed to determine the copy number for all plasmid forms (*_e_* Gen2 and *_e_* Gen2^Δ^). The results shown are mean ± standard deviation of five biological replicates (plants 1–5) and three technical replicates per biological replicate. (b) Images showing 10‐week‐old *_e_* Gen2‐containing line 2, an *_e_* Gen1‐containing line, and a Gen1‐integrating transplastomic line compared to wild‐type control plants. Scale bar: 10 cm. Graphs represent various plant characteristics: (c) Plant height; (d) number (N) of nodes; (e) total fresh weight; (f) total dry weight; (g) ratio of leaf fresh weight (g) to foliar area (m^2^); (h) ratio of leaf dry weight (g) to foliar area (m^2^); (i) chlorophyll content index (CCI); and (j) leaf CO2 assimilation (µmol/m^2^/s). The results are expressed as mean ± standard deviation of five plants per each transgenic line and wild‐type control. For all graphs means separation was evaluated using ANOVA Tukey HSD (*P* < 0.05). Statistical significance is indicated by different letters.

In *_e_* Gen2‐containing lines 1–3 and *_e_* Gen2^Δ^‐containing lines, the episomes persisted; however, the copy number and ratio of *_e_* Gen2 to *_e_* Gen2^Δ^ varied by line. In *_e_* Gen2‐containing line 2, it was possible to recover both *_e_* Gen2^Δ^ and *_e_* Gen2, whereas the other lines consisted exclusively of *_e_* Gen2 or *_e_* Gen2^Δ^ (Figure 3e, graph all forms vs *_e_* Gen2 only; Figure S12). Further, the lines containing only *_e_* Gen2 (*_e_* Gen2‐containing lines 1 and 3) were present at lower average copy number compared to the native plastome, ~0.65 and 0.9 copies per plastome, whereas lines containing only *_e_* Gen2^Δ^ or a mix of *_e_* Gen2 and *_e_* Gen2^Δ^ had an average copy number exceeding that of the native plastome, 2.63 ± 1.15 and 1.76 ± 1.13, respectively (Figure 3e, graph all forms). In order to test the stability of *_e_* Gen2 throughout the complete life cycle of potato, a growth experiment using three independent *_e_* Gen2‐containing lines was initiated. For comparison, an *_e_* Gen1‐containing line, an integrating Gen1 line, and wild‐type plants were used as controls. For each line, five plants were used as biological replicates, as it was anticipated that there may be plant to plant variation due to the constant potential for recombination within the homology arms. Recombination leading to the emergence of *_e_* Gen2^Δ^ from *_e_* Gen2 and the persistence of these two forms were investigated at different developmental stages until anthesis (Figure 4; Figure S13). After 2 weeks in tissue culture (without selection) and 1 week in pots, all analysed plants carried only *_e_* Gen2 with the exception of one plant from line 2 which had *_e_* Gen2^Δ^ (Figure S13). After 4 weeks on potting mix, all *_e_* Gen2‐containing line 1 plants and 4 out of 5 *_e_* Gen2‐containing line 3 plants had undetectable levels of either episomal construct, whereas all *_e_* Gen2‐containing line 2 plants maintained episomal constructs (two carrying *_e_* Gen2 and three only *_e_* Gen2^Δ^). At 7 and 10 weeks (anthesis) all *_e_* Gen2‐containing line 2 plants were stably harbouring only *_e_* Gen2^Δ^ (Figure S13). For all *_e_* Gen2‐containing plants where the episomal construct could no longer be detected by PCR of the vector backbone, the transgenes were also not detected, confirming the complete elimination of recombination with the native plastome and restoration of the wild‐type genotype (Figure S13). For all *_e_* Gen2‐containing line 2 plants, episomal constructs could be recovered throughout the entire life cycle through anthesis, with an average of ~1–1.5 copies per plastome maintained from 1–10 weeks on potting mix without selection (Figure 4a). In *_e_* Gen2‐containing line 2 plants, the large standard deviations of copy number of the episomal construct reflect the high variability observed in different plants growing in tissue culture and on potting mix (Figures 3e and 4a).

In order to determine whether there was pleiotropic or genetic load effects that were caused by the persistence of the episomal plasmid, the phenotype of *_e_* Gen2‐containing line 2 plants along with wild‐type and transplastomic (*_e_* Gen1‐containing and integrating Gen1) control plants grown for 10 weeks in pots (Figure 4b) was analysed. Plant height and number of nodes as well as total fresh and dry weight were not significantly different between *_e_* Gen2‐containing line 2 plants and wild‐type control plants (Figure 4c–f). Considering leaf biomass per unit of foliar area, the *_e_* Gen2‐containing line 2 plants accumulated congruent fresh biomass (Figure 4g) but ~20% less dry biomass than wild type (Figure 4h). However, there was no significant difference between *_e_* Gen2‐containing line 2 plants and the transplastomic controls (Figure 4h). No difference was observed in both chlorophyll content index (CCI) and leaf CO_2_ assimilation between *_e_* Gen2‐containing line 2 and wild‐type plants, indicating there was congruent chloroplast function among lines (Figure 4i,j).

### Conclusions and future perspectives of the mini‐synplastome platform

**Figure 5:**
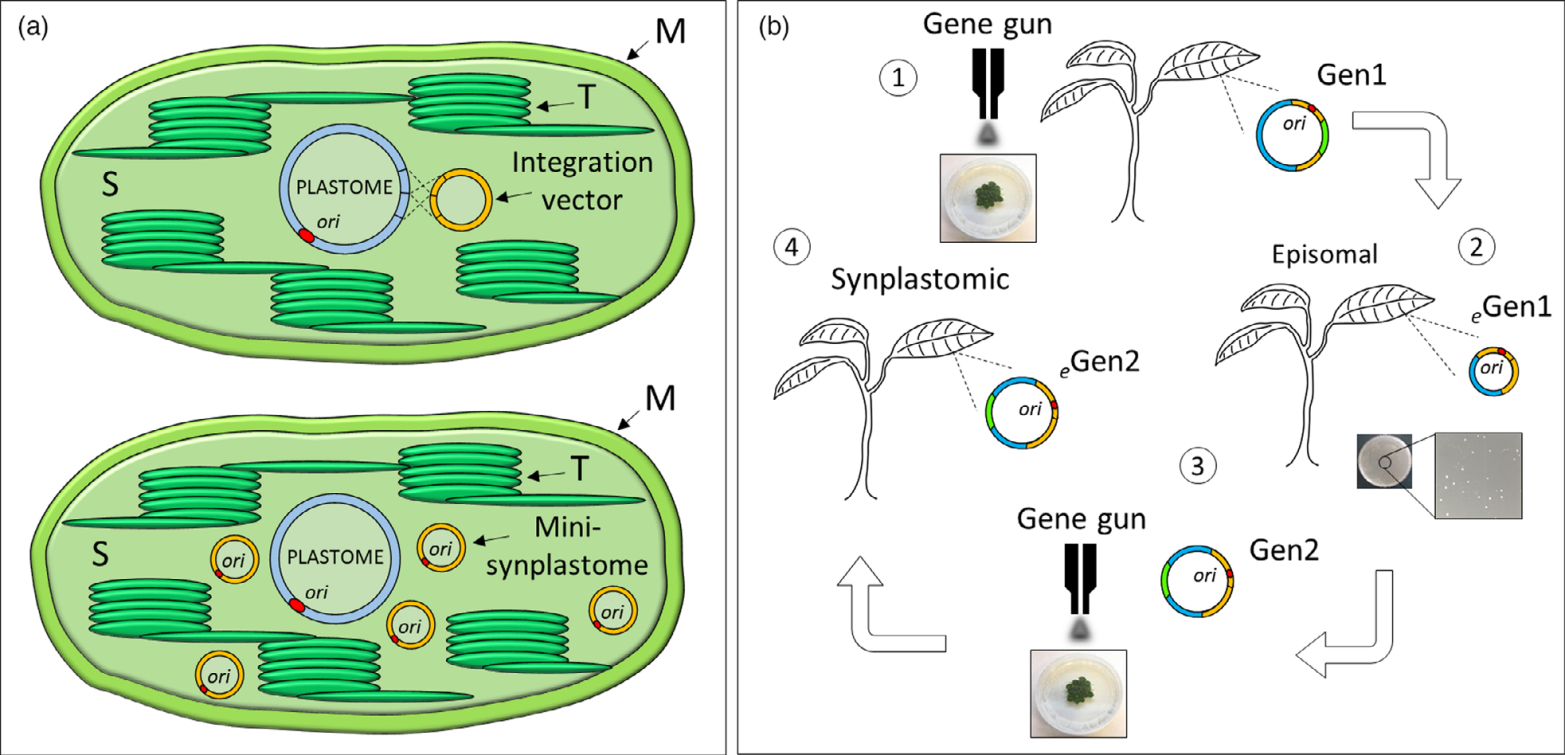
Plastid genetic engineering using the mini‐synplastome. (a) Different methods of chloroplast transformation. The traditional method of chloroplast transformation is based on the utilization of vectors able to integrate into the plastome by homologous recombination (top image). A new method of chloroplast transformation based on the utilization of non‐integrating episomal vectors able to persist as small independent synthetic plastomes, mini‐synplastomes (bottom image). The chloroplast genome (plastome), integration vector, and mini‐synplastomes are indicated in the chloroplast stroma (S). The chloroplast origin of replication *(ori*, in red), thylakoids (T) and chloroplast membranes (M) are also indicated. (b) The design–build–test cycle used to develop the mini‐synplastome platform for chloroplast transformation: (1) Biolistic transformation of leaf tissue using chloroplast‐specific integration vectors (Gen1) and selection for transplastomic lines without vector integration; (2) screening for lines containing the episome, *_e_* Gen1, and isolation of this plasmid by back‐transformation into *E. coli*; (3) based on sequence analysis informed from *_e_* Gen1, Gen1 was used as backbone to synthesize the Gen2 plasmid which was used to transform chloroplasts; (4) synplastomic lines containing the episome *_e_* Gen2 were generated. Homologous arms (yellow), chloroplast *ori* (red), and transgene cassette (green) are indicated in the plasmids.

In this study, we describe a novel platform for plastid genetic engineering that relied on small synthetic genomes, named mini‐synplastomes (Figure 5). While this was not the first attempt to use episomal vectors for plastid engineering, this work has made significant advances over previous attempts. Perhaps the most significant advancement was the persistence of the mini‐synplastome across multiple generations after the removal of selection. Although autonomously replicating chloroplast vectors were maintained in cultured cells (Daniell *et al*., 1990), episomal plastid engineering failed to achieve persistence of the episomal construct in transplastomic plant lines without selection. In fact, even with selection, an episomal plastid engineering construct designed with a dinoflagellate *ori* was lost after multiple generations in tissue culture (Min *et al*., 2015a). The authors hypothesized that the inability to sustain the episome was the result of decreasing copy number throughout the multiple generations. Both the *_e_* Gen1 and *_e_* Gen2 mini‐synplastomes had a stable copy number (~2.0 *_e_* Gen1 and ~1.0 *_e_* Gen2, relative to the plastome copy number) even after >10 weeks in pots without selection. The ability to grow the mini‐synplastomic plants in pots without selection also enabled us to conduct the first phenotypic study of episomal plastid engineered plants (Figure 4). Phenotypic analysis of *_e_* Gen1 and *_e_* Gen2 engineered plants demonstrated that there was no fitness penalty to using the mini‐synplastome strategy for chloroplast engineering, again demonstrating the utility of the approach. Persistence without selection is a key determinant and requirement for any commercial application of episomal plastid engineering.

Another advancement over previous work was the quantitative analysis of transgene expression from episomal constructs in whole plants. Early work showed increase in expression of foreign genes with autonomously replicating chloroplast vectors when compared to vectors without chloroplast ORI in cultured cells (Daniell *et al*., 1990), but this was not demonstrated in transplastomic plant lines. Further work demonstrated that episomal constructs were able to express marker genes without integration (Min *et al*., 2015a; Staub and Maliga, 1995); however, no attempts were made to quantitate gene expression from these episomes for the purposes of heterologous protein production. In the current work, we demonstrated that some *_e_* Gen2 lines were capable of achieving equivalent expression of heterologous genes to the current state‐of‐the‐art HR‐based plastid engineering. In hybrid *_e_* Gen1 lines it was possible to exceed the expression levels of HR‐based plastid engineering (Figure 3c), providing evidence that further improvements to the mini‐synplastome design may represent a more effective strategy for heterologous protein production for chloroplast biotechnology.

Based on the results obtained from this work, mini‐synplastomes provide significant promise as an alternative to traditional HR‐based plastid engineering. For chloroplast biotechnology, the ability to express transgenes without the requirement for integration of foreign DNA into the host genome represents a paradigm shift. With regard to the regulatory landscape, chloroplast biotechnology has long remained in the ‘grey area’ since HR can be achieved without the use of any plant pathogens or pathogenic sequences, thus resulting in exemption from USDA‐APHIS regulation 7 CFR part 340 (Daniell *et al*., 2019b). However, in order for commercialization of chloroplast engineered plants, it is desirable for selection genes to be removed prior to approval. To date, marker‐free transplastomics have been generated through the use of recombinases (Corneille *et al*., 2001; Hajdukiewicz *et al*., 2001), loop‐out recombination (Ruf *et al*., 2007), transient cointegration of two chloroplast vectors (Klaus *et al*., 2004), or direct repeat recombination of transgenes integrated into the large single copy region of tobacco (Iamtham and Day, 2000), soybean (Dufourmantel *et al*., 2007) or the inverted repeat region of lettuce (Daniell *et al*., 2019b, 2020; Kumari *et al*., 2019; Park *et al*., 2020; Singh *et al*., 2021). As demonstrated in this work, it is possible to design integrating episomal constructs, where the desired transgenes are incorporated into the native plastome through HR, while the selection genes are expressed on the episomal construct. In this scenario, expression of the selection gene from a non‐integrating region of the episome will be the driving force to achieve homoplasmy, as the compartment itself is still being selected against the selection agent. Once homoplasmy has been achieved, the selective pressure could be removed, enabling the gradual elimination of the episomal vector, if designed for that outcome.

In the Gen1 design, it was demonstrated that transgenes could be inserted into the native plastome and the episomal backbone could still persist. In addition, we have demonstrated that the design of the mini‐synplastome copy number can be affected by its design and likely the ‘dose’ of mini‐synplastome delivered in the initial transformation event. As such, we hypothesize that a mini‐synplastome could be designed whereby the transgene cassette integrates into the native plastome, while the selection cassette remains on the mini‐synplastome backbone. In this case it would be possible to use selection within the plastid compartment (enabled by expression of the selection gene from the mini‐synplastome backbone) to drive homoplasmy of the native plastome with the transgene insert. Once homoplasmy has been achieved, selection could be removed, which would lead to the gradual elimination of the episome and generation of a marker‐free transplastomic line. Facile methods for marker‐free chloroplast engineering would be advantageous in a germplasm development pipeline.

In this work, engineered episomal constructs were demonstrated to persist throughout the life cycle of potato plants and multiple vegetative generations, regardless of whether or not transgenes were expressed from the episomal construct, or integrated into the native plastome. Earlier work demonstrated that copy number has a direct impact on the expression level of foreign genes integrated into the chloroplast genome; integration of *GFP* into the *trnI/trnA* locus of the inverted repeat region resulted in 25‐fold higher expression than integration into the large single copy region (*rbcL/accD*) (Krichevsky *et al*., 2010). Compared to the current state of the art in plastid engineering (integration of a transgene into the *trnI/trnA* site by HR (Krichevsky *et al*., 2010)), *_e_* Gen1‐containing lines with a persisting episome and unpredicted transgene integration into the plastome had up a 2.5‐fold increase in transgene expression (Figure 3c). One explanation for the increase may be the potential for the transgene cassette to constantly recombine between the episomal construct and the plastome, leading to increased copy number of the transgene cassette. Previous work has shown that inclusion of the chloroplast origin of replication within the chloroplast DNA flanking sequence used for transgene integration (Lugo *et al*., 2004; Nielsen *et al*., 1993) facilitated achieving homoplasmy even after the first round of selection, likely due to increased copy number (Daniell *et al*., 1998; Guda *et al*., 2000). Thus, while not the ultimate goal of this work, the generation of hybrid integrating episomal constructs has provided a path forward for even higher levels of heterologous protein production than previously possible.

While demonstrated here in potato, episomal plastid engineering is not relegated to land plants and represents a strategy that may be utilized for engineering plastids of other species, such as algae, or other organellar genomes, such as mitochondria. In addition, the lack of a requirement for homology arms would extend the portability of the mini‐synplastome strategy between plant species with heterologous plastome sequences, which would not be possible with traditional HR‐based chloroplast engineering. The simplicity of the approach, and the ability to express transgenes at levels similar to HR‐based plastid engineering, validates its use for production of heterologous proteins for use in a variety of fields, such as biopharming, enzyme production, and crop improvement. The prospect to design improved mini‐synplastome versions able to be maintained at higher copy number vs the endogenous plastome will potentially improve heterologous protein accumulation in chloroplasts compared to traditional techniques.

Further refinement of the episomal strategy using chloroplast‐specific *ori* from different organisms, including higher plants and algae (Carrillo and Bogorad, 1988; Chiu and Sears, 1992; Daniell *et al*., 1990; Karas *et al*., 2015; Kunnimalaiyaan and Nielsen, 1997; Meeker *et al*., 1988; Nisbet *et al*., 2004; Waddell *et al*., 1984; Wang *et al*., 1984), and the use of single or multiple *ori* with different activities could enable precise tuning of the copy number of the episomal constructs, adding another layer for control of gene expression in complex pathways. In essence, this work demonstrates a novel technology with significant improvements on the current state of plastid engineering, which should enable synthetic biology in plants.

## Experimental procedures

### Construction of transformation vectors

For construction of Gen1 and pSSC plasmids, synthetic sequences homologous to the IR (*trnI*/*trnA*; 102623–105457 bp and 105458–110067 bp; GenBank: KU199713.1) and SSC (*ndhG*/*ndhI*; 119184–120988 bp and 120989–126029 bp; GenBank: KU199713.1) regions of the tobacco (*Nicotiana tabacum*) plastome were used. These sequences were synthesized by GeneArt (Thermo Fisher Scientific, Waltham, MA) and cloned into the pMK vector. The chloroplast‐specific dual selection cassette (*Prrn‐SD::aadA::5′UTR::mGFP::3′UTR*) of the PLD‐PTD‐GFP plasmid (Kwon *et al*., 2013) was PCR amplified and blunt‐cloned into the *Pme*I site of either IR (*trnI/trnA*) or SSC (*ndhG/ndhI*) synthetic sequences generating the Gen1 and pSSC plasmids. The pair of primers 1 Fw/Rv were used to amplify the selection cassette for Gen1 and pSSC plasmids. For construction of Gen2, the dual selection cassette of Gen1 was amplified using the pair of primers 2 Fw/Rv adding a *Psi*I restriction sites at both 5*′* and 3*′* ends. Gen2 was then constructed by cloning the selection cassette into the *Psi*I site located in the backbone of Gen1.

For sequence analysis of integration, plasmids containing ~1.7 kb plastomic sequence located at the *trnI*/*trnA* integration site of wild‐type potato and lines containing *_e_* Gen1, *_e_* Gen2^Δ^, and *_e_* Gen2 lines 1–3 were constructed by cloning a PCR amplicon obtained using genomic DNA and 3 Fw/Rv primers into *Kas*I/*Hind*III restriction site of the pUC plasmid (Norrander *et al*., 1983). These plasmids were then transformed into *E. coli* and isolated for sequencing. The sequences of primers used in this study are shown in Table S3.

### Plant growth *in vitro* conditions

*Solanum tuberosum* ‘Desirée’ (potato) plants were grown in sterile conditions in Magenta GA7 boxes containing MS Reg media (4.33 g/L Murashige and Skoog (MS) basal salt mixture; 25 g/L sucrose; 100 mg/L myo‐inositol; 170 mg/L sodium phosphate monobasic monohydrate; 440 mg/L calcium chloride dihydrate; 0.9 mg/L thiamine‐HCl; 2 mg/L glycine; 0.5 mg/L nicotinic acid; 0.5 mg/L pyridoxine‐HCl; 1X MS vitamins; 3 g/L phytagel; pH 5.8). Transplastomic lines were grown in selective MS rooting media (4.33 g/L MS basal salt mixture; 1X Gamborg B5 vitamins; 30 g/L sucrose; 200 mg/L spectinomycin; 3 g/L phytagel; pH 5.8). All reagents for tissue culture were purchased from Phyto Technology Laboratories (Lenexa, KS), except spectinomycin dihydrochloride pentahydrate, which was purchased from Millipore (Billerica, MA). The purity of spectinomycin is critical to ensuring efficient selection of transplastomic lines. Both wild‐type and transgenic plants were kept in a controlled environmental room at 16 h of light and 8 h of dark. The temperature was kept at 22–24 °C during all light/dark cycle. Tissue culture/selection/regeneration steps for generation of transplastomic lines were performed in the same controlled environment.

### Production of transplastomic lines

The gene gun PDS‐1000/He delivery system (Bio‐Rad, Hercules, CA) was used to transform chloroplasts (Occhialini *et al*., 2019). Transplastomic plants were obtained from transformed leaf material by applying a tissue culture/selection/regeneration protocol as described previously (Valkov *et al*., 2011). Approximately 6 cm^2^ of leaf tissue collected from one month‐old potato plants grown in sterile conditions was placed in the centre of a Petri dish containing M6M media (4.33 g/L MS basal salt mixture; 1X Gamborg B5 vitamins; 30 g/L sucrose; 18.2 g/L mannitol; 18.2 g/L sorbitol; 0.8 mg/L zeatin riboside (ZR); 2 mg/L 2,4‐dichlorophenoxyacetic acid (2,4‐D); 3 g/L phytagel; pH 5.8). The tissue was kept overnight in the dark at room temperature before transformation. For transformation, 0.3 mg of 0.6 µm gold‐particles were used to bind 1 µg of plasmid following the manufacturer’s protocol (Seashell Technology, La Jolla, CA). Tissue was bombarded at 6 cm‐distance using 1100 psi rupture disks. After two days of incubation in the dark at room temperature, leaf material was cut in small pieces (5 mm^2^) and placed in selective M6 media (4.33 g/L MS basal salt mixture; 1X Gamborg B5 vitamins; 30 g/L sucrose; 0.8 mg/L zeatin riboside (ZR); 2 mg/L 2,4‐dichlorophenoxyacetic acid (2,4‐D); 400 mg/L spectinomycin; 3 g/L phytagel; pH 5.8) at the growth condition described previously. After one‐month incubation, the tissue was transferred to selective Ti media (4.33 g/L MS basal salt mixture; 1X Gamborg B5 vitamins; 16 g/L glucose; 3 mg/L zeatin riboside (ZR); 2 mg/L indole acetic acid (IAA); 1 mg/L gibberellic acid (GA_3_); 400 mg/L spectinomycin; 3 g/L phytagel; pH 5.8). 4–8 weeks later, transplastomic green callus was obtained from transformed leaves. Green callus was transferred to selective DH media (2.16 g/L MS basal salt mixture NH_4_NO_3_^‐^ free; 268 mg/L NH_4_Cl; 1X Nitsch vitamin mixture; 2.5 g/L sucrose; 36.4 g/L mannitol; 100 mg/L casein hydrolysate; 80 mg adenine hemisulphate; 2.5 mg/L ZR; 0.1 mg/L IAA; 400 mg/L spectinomycin; 3 g/L phytagel; pH 5.8) for another month of growth, and after that placed on MON media (4.33 g/L MS basal salt mixture; 1x Gamborg B5 vitamins; 30 g/L sucrose; 0.1 mg/L naphthaleneacetic acid; 5 mg/L ZR; 400 mg/L spectinomycin; 3 g/L phytagel; pH 5.8) for regeneration of shoots. Primary transplastomic shoots were transferred in Magenta boxes containing selective MS rooting media for roots regeneration. For the second and third round of transplastomic plants, the same protocol of selection/regeneration was performed.

### Plant phenotypic analysis

To synchronize plant growth, apical shoots were collected from plants of the same age and then transferred in Magenta boxes containing MS Reg media without selection. After 2 weeks of growth *in vitro*, wild‐type and transgenic plantlets with roots were transferred in 3.8 l pots containing soil Pro‐Mix BK25 (Griffin Greenhouse Supplies, Inc. Tewksbury, MA) and kept growing for 10 weeks until anthesis in a controlled environment (16 and 8 h of light/dark cycle at the temperature of 22–24 °C). At this developmental stage, plant height (cm), number of nodes, foliar and total plant fresh, and dry weight (g) were collected. Before collecting the dry weight data, plant tissue was dried for 1 week at 50 °C. ImageJ 1.41o software (National Institute of Health, Bethesda, MD) was used to calculate the leaf area by image analysis of leaf scans (Occhialini *et al*., 2016).

The LI‐6800 portable photosynthesis system (LI–COR Biosciences, Lincoln, NE) was used to determine the CO_2_ assimilation (A) values per unit of leaf area (µmol/m^2^/s) of both wild‐type and transplastomic plants. The leaf gas exchange measurements were performed at a temperature of 23 °C, 400 µmol mol air⁻¹ of CO_2_, 1000 µmol photons m⁻² s⁻¹ of irradiance 0.8–1.2 kPa of vapour‐pressure deficit (VPD leaf), and a 200 µmol/s of flow rate (Occhialini *et al*., 2020). The portable CCM‐200 plus chlorophyll content meter (OPTI‐SCIENCES Inc., Hudson, NH) was used to determine the leaf CCI. The IBM SPSS software (IBM, New York, NY, USA) was used to perform statistical analysis, and means were compared using ANOVAs with post‐hoc Tukey (*P* < 0.05).

### Total DNA extraction and PCR analysis

For extraction of total genomic DNA from leaves a CTAB‐based procedure was used (Occhialini *et al*., 2020). Approximately 50 mg of leaf tissue frozen in liquid nitrogen was finely ground in an Eppendorf tube. The macerated leaf material was resuspended in 500 μL of CTAB extraction buffer (2% hexadecyltrimethyl ammonium bromide; 1% (w/v) polyvinyl pyrrolidone; 100 mm Tris–HCl; 1.4 m NaCl; 20 mm EDTA; 0.1 mg/ml RNaseA), thoroughly vortexed and incubated for 10 min at room temperature. Samples were further incubated for 30 min at 60 °C, and then the cellular debris was eliminated by centrifugation at 15 000 ***g*** for 5 min. An equal volume of a solution containing chloroform/isoamyl alcohol (24 : 1) was added to the clarified supernatant. The sample was vortexed for 5 s and centrifuged at 4 °C for 1 min at 15 000 ***g***. The upper aqueous phase was transferred in a new tube, and the DNA was precipitated by adding an equal volume of ice‐cold dry isopropanol. The samples were incubated for 30 min on ice and then centrifuged at 4 °C for 30 min at 15 000 ***g***. The DNA precipitated in the tube was washed in 500 μL of ice‐cold 75% (v/v) ethanol. The air‐dried pellet was resuspended in 50–100 μL of sterile H_2_O and quantified using a Nanodrop spectrophotometer.

Two pairs of primers 4 Fw/Rv and 5 Fw/Rv were used to check the integration in the IR (*trnI/trnA*) and SSC (*ndhG/ndhI*) sites of the plastome. The two pair of primers 6 Fw/Rv and 7 Fw/Rv were used to check for the presence of full‐length s*mGFP* (encoding soluble monomeric green fluorescent protein, NCBI ID: AEX93343.1) and *aadA* (encoding the streptomycin 3″‐adenylyltransferase, NCBI ID: AAR14532.1) genes, respectively. The pairs of primers 8 Fw/Rv and 9 Fw/Rv were used to check the presence of *KanR* (encoding aminoglycoside 3′‐phosphotransferase, GenBank: APB62235.1) and *SpcR* (spectinomycin adenyltransferase, GenBank: AAA72848.1) selective genes of the backbone, respectively. The primers 10 Fw/Rv were used to amplify an internal fragment of *rbcL* (*S. tuberosum* ribulose‐1,5‐bisphosphate carboxylase/oxygenase large subunit, NCBI ID: 4099985) used as loading control. The sequences of primers used in this study are shown in Table S3.

### *E. coli* back‐transformation with episomal vectors extracted from leaf tissue

A total volume of 25 µL of chemically competent *E. coli* TOP10 (Thermo Fisher Scientific) were transformed using 500 ng of genomic DNA from episomal lines using the heat‐shock method. Transformed cells were grown in Luria‐Bertani agar media (10 g/L of bacto‐tryptone; 5 g/L of yeast extract; 10 g/L NaCl; 15 g/L bacto agar; pH 7) containing 50 µg/ml kanamycin. Pure preparations of episomal DNA (*_e_* Gen1, *_e_* Gen2 and *_e_* Gen2^Δ^) were extracted from bacterial cells using QIAprep Spin Miniprep Kit (QIAGEN, Valencia, CA). The presence of left and right homologous arms, along with the internal cassette, was tested by PCR using the pair of primers 11 Fw/Rv, 12 Fw/Rv and 13 Fw/Rv, respectively. The sequences of primers used in this study are shown in Table S3. The nucleotide sequence of extra‐plastomic DNA was determined by Sanger DNA sequencing (Massachusetts General Hospital MGH, Center for Computational & Integrative Biology CCIB, DNA Core, Boston, MA).

### Total RNA extraction and qRT‐PCR

Total RNA preparations were obtained from leaf tissue collected from both transgenic lines and wild‐type controls using the Tri‐Reagent (Molecular Research Center, Inc, Cincinnati, OH) according to manufacturer’s protocol. For each RNA extraction, ~50 mg of fresh leaf tissue was used. Thereafter, RNA preparations were subjected to DNase treatment followed by cleaning using the RNA Clean & Concentrator Kit (Zymogen, Irvine, CA) according to manufacturer’s instruction. The Super Script III Reverse Transcriptase (Thermo Fisher Scientific) was used to synthesize the first cDNA strand following the manufacturer’s instruction. PCRs using several cDNA dilutions were performed to test the presence of full‐length sequence of the two transgenes of the cassette, *aadA* (aminoglycoside‐3″‐adenylyltransferase, GenBank: ARK38551.1) and s*mGFP* (soluble monomeric green fluorescent protein, GenBank: AEX93343.1). The *rbcL* (*Solanum tuberosum* ribulose‐1,5‐bisphosphate carboxylase/oxygenase large subunit, NCBI ID: 4099985) and *ef1α* (*Solanum tuberosum* elongation factor 1‐alpha; XM_006343390.2) genes were used as internal reference for plastome and nuclear genome, respectively. The pair of primers 6 Fw/Rv, 7 Fw/Rv, 14 Fw/Rv, and 15 Fw/Rv were used to detect s*mGFP*, *aadA*, *rbcL*, and *ef1α*, respectively (Table S3).

### qPCR for copy number determination

qPCR was performed in a total volume of 15 µL in 1X PowerUp™ SYBR™ Green Master Mix (Thermo Fisher Scientific), using 5 ng of pure genomic DNA and 0.5 µm of both forward and reverse primers. The pairs of primers 16 Fw/Rv and 17 Fw/Rv were used to detect the backbone gene *KanR* of all episomal plasmids (encoding aminoglycoside 3'‐phosphotransferase, GenBank: APB62235.1) and a unique sequence of *_e_* Gen2 backbone, respectively. The plastome internal control *rbcL* (*Solanum tuberosum* plastome, NCBI Gene ID: 4099985) and the nuclear gene *actin PoAc58* (X55749.1) were detected using the pair of primers 14 Fw/Rv and 18 Fw/Rv. For episomal vs plastome copy number determination, 0.1, 1, 20, 40, 60, 80, 100, 120, 140, 160, 180, and 250 pg of purified episomal plasmid (either *_e_* Gen1 or *_e_* Gen2) were used as standards of copy number. Per each standard, the DNA copy number was calculated using the equation: (ng DNA × 6.022 × 10^23^)/(DNA length bp × 1 × 10^9^ × 650). Linear regression graphs plotting Ct values of DNA standards (*Y* axis) vs log10 of copy number of standards (*X* axis) were used to calculate the copy number of both episomal plasmid and plastome in genomic DNA samples. Wild‐type plants and blanks were used as negative controls. Microsoft Excel software was used to process data and for their graphical representation.

qRT‐PCR was also performed in a total volume of 15 µL in 1X PowerUp™ SYBR™ Green Master Mix (Thermo Fisher Scientific), using cDNA diluted 1 to 3 and 0.5 µm of each primer. The pair of primers 19 Fw/Rv and 20 Fw/Rv were used to amplify the *aadA* (aminoglycoside‐3″‐adenylyltransferase, GenBank: ARK38551.1) and s*mGFP* (soluble monomeric green fluorescent protein, GenBank: AEX93343.1) gene of the selection cassette, respectively, whereas 15 Fw/Rv were used to amplify the internal reference gene *ef1α* (*S. tuberosum* elongation factor 1‐alpha; XM_006343390.2). The relative gene expression data were represented using the $2^{‐\Delta C_{T}}$ method.

qPCR and qRT‐PCR primers were designed to amplify a fragment of ~100 bp at a compatible annealing temperature of ~57 °C using the online software Primer3 input v. 0.4.0 (Howard Hughes Medical Institute and by the National Institutes of Health) (Koressaar and Remm, 2007; Untergasser *et al*., 2012). The sequences of primers used in this study are shown in Table S3. qPCR and qRT‐PCR were performed using a QuantStudio™ 6 Flex Real‐Time PCR System (Thermo Fisher Scientific), whereas data were acquired using the QuantStudio™ Real‐Time PCR Software v1.1 (Thermo Fisher Scientific). The data are expressed as mean ± standard deviation (SD) of the indicated biological and technical replicates. The IBM SPSS (IBM, New York, NY, USA) software was used to perform statistical analysis, and means separation was evaluated using ANOVA Tukey HSD (*P* < 0.05).

### Southern blot analysis

The inverted repeat region (IR) of the chloroplast genome of potato (*S. tuberosum*; GenBank: NC_008096.2; from 104457 to 104978 bp) was used to design a DNA probe for detection of Gen1 vector integration. The *KanR* gene sequence from the vector backbone pMK GeneArt (Thermo Fisher Scientific) was used to design the probe to detect extra‐plastomic DNAs (*_e_* Gen1 or *_e_* Gen2). The *aadA* gene (GenBank: ARK38551.1) was used to design to probe to detect the selection cassette. The IR, *KanR*, and *aadA* DNA‐probes labelled with digoxigenin‐(DIG)‐sUTP were synthesized using the PCR DIG Probe Synthesis Kit (Roche, Indianapolis, IN) and the pair of primers 21 Fw/Rv, 22 Fw/Rv and 23 Fw/Rv, respectively. 1 µg of total genomic DNA from leaf tissue was digested using *Kas*I/*Hind*III or *Fsp*I/*Fse*I restriction enzymes, for detection of IR and *aadA* or *KanR* fragments, respectively. DNA samples were separated on 0.9% agarose gel, depurinated, denatured, and then transferred on Hybond‐N+ nylon membrane (GE Healthcare, Life Sciences, Marlborough, MA) (Lin *et al*., 2014). The anti‐digoxigenin‐AP Fab fragments detection kit (Roche) was used for detection of the DIG‐labelled probe signal.

### Confocal microscopy

Healthy leaves from 3–4‐week‐old wild‐type potato plants and transplastomic lines grown in sterile conditions were imaged using an Olympus Fv1000 confocal microscope (Olympus, Center Valley, PA) equipped with both traditional (argon and HeNe) and diodes (405, 440, 473, 559 and 635‐nm) lasers. The green fluorescent protein (smGFP) was excited at 488 nm and detected at 509 nm of wavelength emission. The chlorophyll auto‐fluorescence was excited using at 543 nm and detected at 667 nm of wavelength emission. Digital images were acquired using Olympus FV10‐ASW Viewer software Ver.4.2a (Olympus). Confocal images were processed using ImageJ 1.41o (National Institute of Health).

## Conflict of interest

The authors declare no competing interests.

## Author contributions

AO, AAP, SCL, and CNS designed the strategy; AO, ACP, LL, SAH, AJ, JNB, and CP collected data; AO and ACP analysed data; HD designed the selection cassette and advised on chloroplast transformation; AO, ACP, AAP, HD, CNS, and SCL wrote the article.

## Supporting information


**Figure S1** Screening for putative Gen1 episome‐containing plants.
**Figure S2** Phenotype of transgenic lines and wild‐type controls.
**Figure S3** Characterization of episomal lines containing *_e_* Gen1.
**Figure S4** PCR characterization of *_e_* Gen1 plasmids extracted from leaf tissue of episome‐containing lines.
**Figure S5** Determination of episome/plastome ratio of *_e_* Gen1‐containing lines at the 3^rd^ round of tissue culture.
**Figure S6** Stability of *_e_* Gen1 episome at different plant developmental stages.
**Figure S7** Characterization of *_e_* Gen1‐containing lines originated from tubers.
**Figure S8** Characterization of synplastomic *_e_* Gen2‐containing lines.
**Figure S9** PCR characterization of *_e_* Gen2 extracted from synplastomic plants.
**Figure S10** Southern blot of synplastomic *_e_* Gen2‐containing lines at the second round of growth in pots without selection.
**Figure S11** Transgene expression in *_e_* Gen2‐containing lines.
**Figure S12** PCRs on bacteria colonies transformed with *_e_* Gen2 contained in leaf tissue.
**Figure S13** Stability of *_e_* Gen2 at different plant developmental stages.
**Table S1** Multi‐sequence alignment of homologous regions.
**Table S2** Multi‐sequence alignment of *trnI*/*trnA* region of potato.
**Table S3** Primers used in this study.

**Table S1** Multi‐sequence alignment of homologous regions.
**Table S2** Multi‐sequence alignment of *trnI*/*trnA* region of potato.
**Table S3** Primers used in this study.
