# Supplementary material for: Mini‐synplastomes for plastid genetic engineering
Source: Plant Biotechnol J. 2021 Oct 24;20(2):360–73. doi: 10.1111/pbi.13717 (PMC8753362; doi:10.1111/pbi.13717)
Supplement: Supplementary file 1 — Figure S1 Screening for putative Gen1 episome‐containing plants. Figure S2 Phenotype of transgenic lines and wild‐type controls. Figure S3 Characterization of episomal lines containing e Gen1. Figure S4 PCR characterization of e Gen1 plasmids extracted from leaf tissue of episome‐containing lines. Figure S5 Determination of episome/plastome ratio of e Gen1‐containing lines at the 3rd round of tissue culture. Figure S6 Stability of e Gen1 episome at different plant developmental stages. Figure S7 Characterization of e Gen1‐containing lines originated from tubers. Figure S8 Characterization of synplastomic e Gen2‐containing lines. Figure S9 PCR characterization of e Gen2 extracted from synplastomic plants. Figure S10 Southern blot of synplastomic e Gen2‐containing lines at the second round of growth in pots without selection. Figure S11 Transgene expression in e Gen2‐containing lines. Figure S12 PCRs on bacteria colonies transformed with e Gen2 contained in leaf tissue. Figure S13 Stability of e Gen2 at different plant developmental stages. Table S1 Multi‐sequence alignment of homologous regions. Table S2 Multi‐sequence alignment of trnI/trnA region of potato. Table S3 Primers used in this study. [file PBI-20-360-s001.pdf]

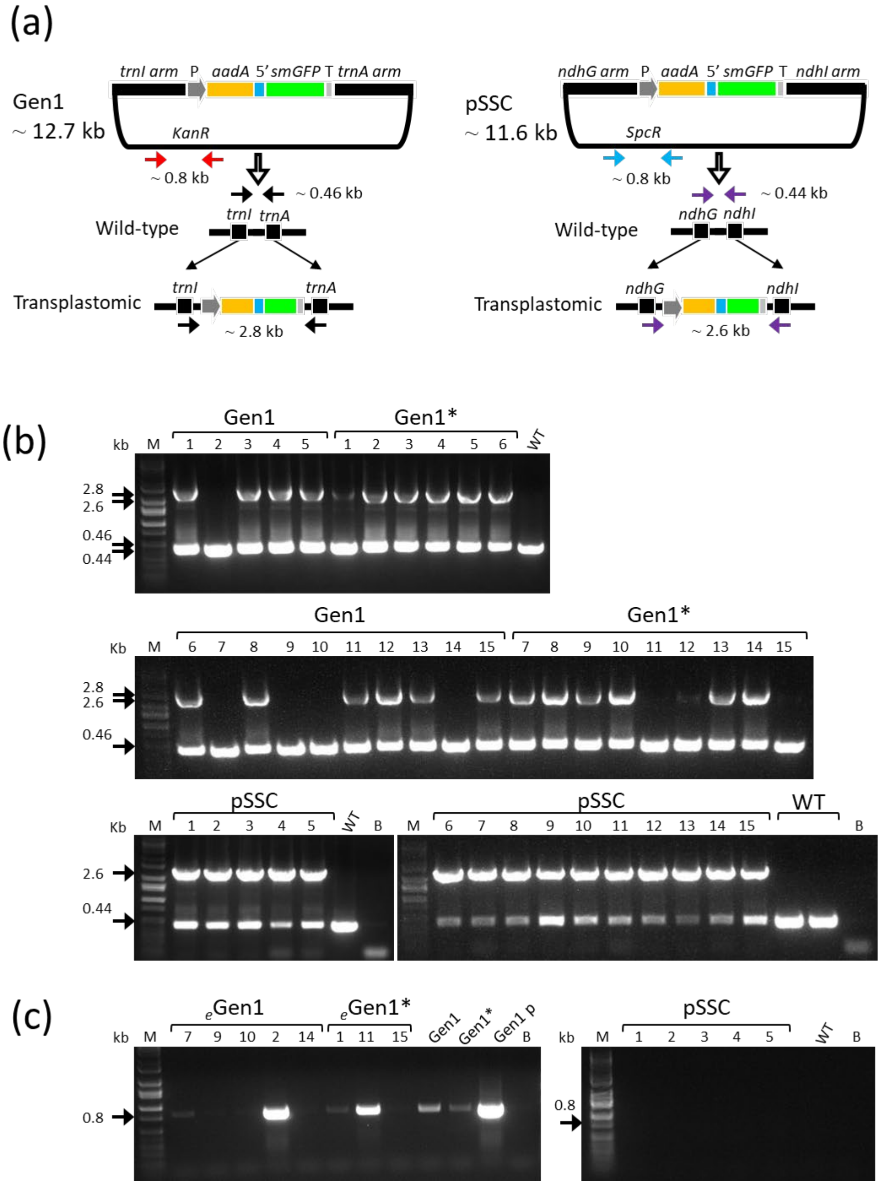

Figure S1: Screening for putative Gen1 episome-containing plants. (a) Schematic representation of Gen1 (~12.7 kb) and pSSC (~11.6 kb) vector integration in the plastome. The *trnI/trnA* and *ndhG/ndhI* homologous arms are indicated in Gen1 and pSCC, respectively. A dual selection cassette is also indicated: Prom-SD (P): rrn promoter along with a Shine-Dalgarno sequence (gray); *aadA*: spectinomycin resistance gene (yellow); 5'UTR: 5' untranslated region (blue); *smGFP*: gene encoding the soluble monomeric green fluorescent protein (green); and 3'UTR (T): 3' untranslated region (light gray). The kanamycin (*KanR*) or spectinomycin (*SpcR*) resistance gene are indicated in Gen1 and pSSC backbones, respectively. Location of primers used to check integration in the *trnI/trnA* (black arrows) and *ndhG/ndhI* (purple arrows) along with primers to check *KanR* (red arrows) and *SpcR* (blue arrows) genes are indicated. **(b)** PCRs to check vector integration in Gen1 and pSSC lines. Gen1-integrating lines originated with another version of the transgene cassette are indicated with asterisk (*). Per each construct, PCR samples from lines 1-15 are shown. DNA bands of 2.8 and 2.6 kb indicate integration of Gen1 and pSSC, respectively. DNA bands of 0.46 and 0.44 kb indicate wild-type (WT) IR and SSC regions of the plastome, respectively. The presence of both transgenic and wild-type bands indicates that all Gen1 and pSSC lines at the first vegetative generation are heteroplasmic. Wild-type (WT) samples, blanks (B) and DNA markers (M; kb) are shown. **(c)** Presence of backbone vector in different transplastomic lines. PCRs using primers for *KanR* (0.8 kb) indicate the presence of the backbone vector in leaf samples of two putative Gen1 lines containing the episome ($_e$Gen1; line 2 and 7). PCRs using primers for *SpcR* confirm the absence of backbone in pSSC-integrating plants. Positive (Gen1 plasmid; p) and negative (Gen1-integrating lines) controls along with wild-type (WT) samples, blanks (B) and DNA markers (M; kb) are shown.

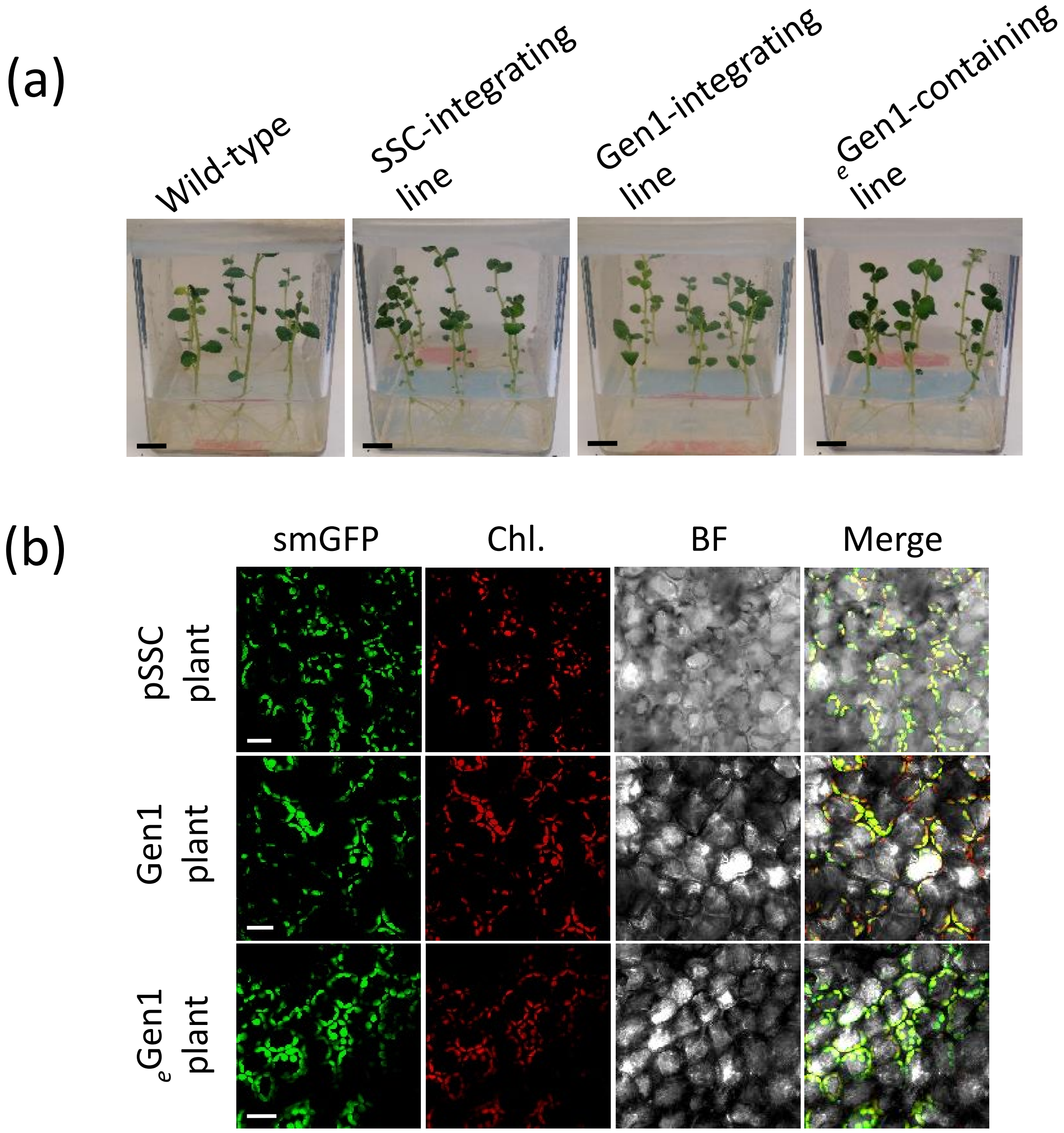

Figure S2: Phenotype of transgenic lines and wild-type controls. (a) Images showing 2-week-old *in vitro* transgenic lines along with wild-type controls. pSSC and Gen1-integrating lines, along with a plant containing $_e$Gen1 are shown. **(b)** Confocal images showing smGFP localization into the chloroplast stroma of leaf mesophyll cells from transgenic lines in A. smGFP (green), chlorophyll (Chl., red), bright-field (BF; gray) and merged images are indicated. Scale bars: 10 mm (a); 20 µm (b).

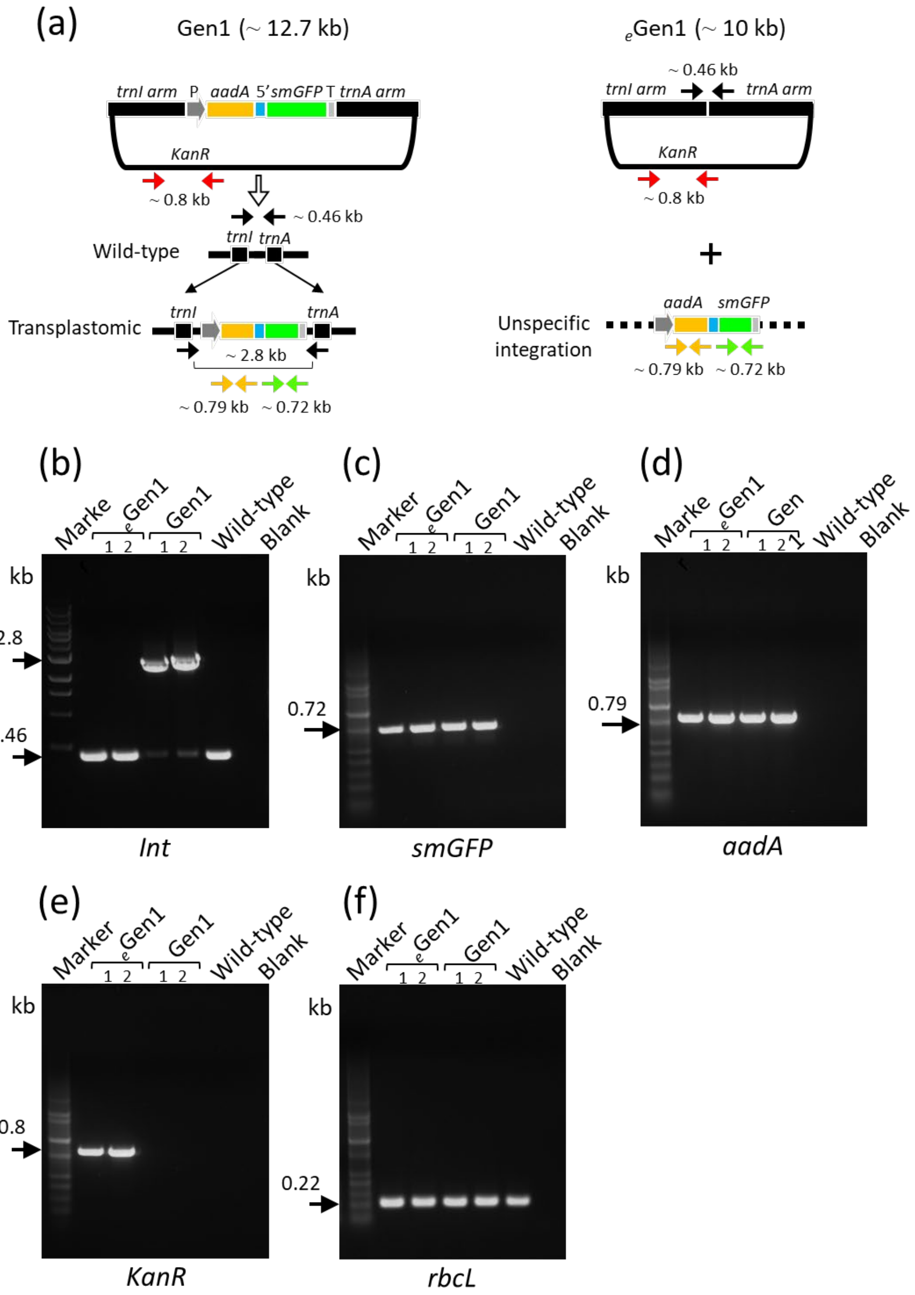

Figure S3: Characterization of episomal lines containing $_e$Gen1. (a) Schematic representation of vector rearrangement in Gen1 (~12.7 kb) integrating and $_e$Gen1 (~10 kb) containing lines. In $_e$Gen1-containing lines the selection cassette is integrated in an unpredicted plastome site. The *trnI/trnA* homologous arms, the kanamycin resistance gene (*KanR*) of the backbone and cassette genetic modules are indicated. The cassette comprises: Prom-SD (P): rrn promoter along with a Shine-Dalgarno sequence (gray); *aadA*: spectinomycin resistance gene (yellow); 5'UTR: 5' untranslated region (blue); *smGFP*: gene encoding the soluble monomeric green fluorescent protein (green); and 3'UTR (T): 3' untranslated region (light gray). Location of primers used to check integration in the *trnI/trnA* region (black arrows), *KanR* (red arrows), *aadA* (yellow arrows) and *smGFP* (green arrows) are indicated. **(b)** PCRs showing vector rearrangement (Int.) in the IR (*trnI/trnA*) region of $_e$Gen1-containing and Gen1-integrating lines, respectively. Two independent lines of both genotypes are indicated (1 and 2). Sample 2 corresponds to the $_e$Gen1-containing line 2 (Figure S1) used in this study. DNA-bands at 2.8 kb indicate transgenes integration into the IR (*trnI/trnA*) site, whereas the presence of 0.46 kb bands indicate wild-type IR regions. **(c-f)** PCRs for s*mGFP* (0.72 kb), *aadA* (0.79 kb), *KanR* (0.8 kb) and *rbcL* fragment (0.22 kb) are also indicated. Wild-type samples and blanks along with DNA molecular markers (kb) are shown.

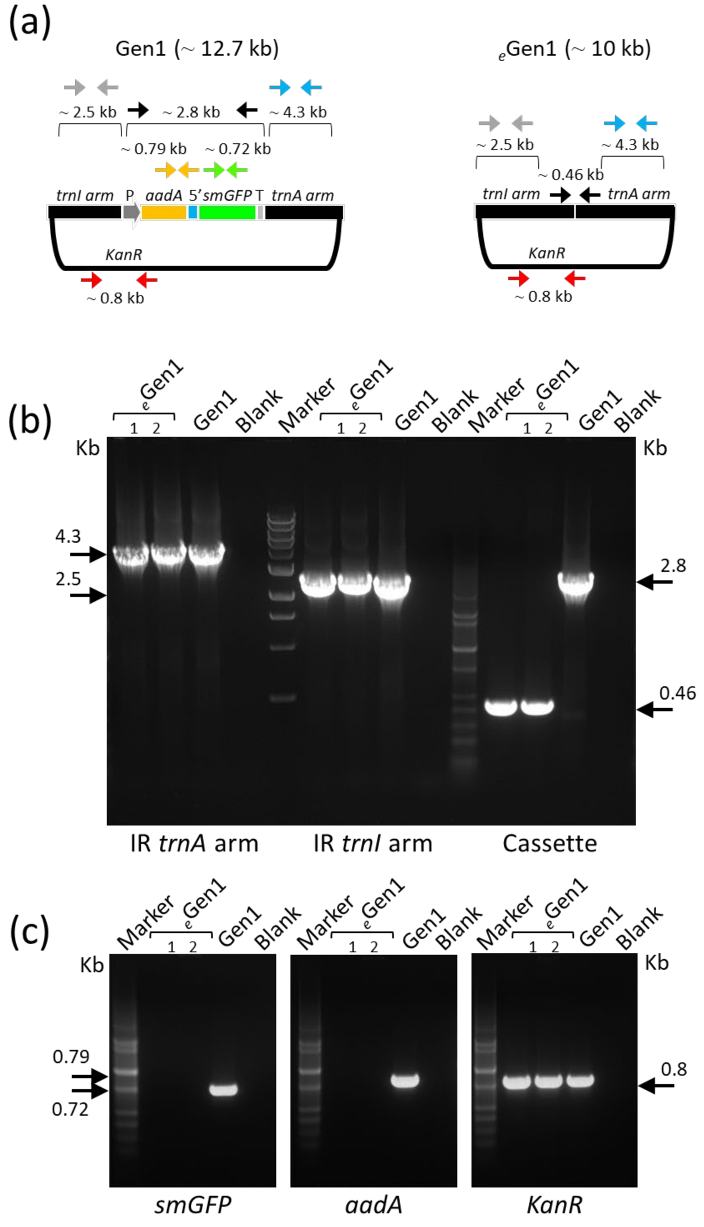

Figure S4: PCR characterization of $_e$Gen1 plasmids extracted from leaf tissue of episome-containing lines. (a) Schematic representation of Gen1 (~12.7 kb) and $_e$Gen1 (~10 kb) plasmids. The *trnI/trnA* homologous arms, the kanamycin resistance gene (*KanR*) of the backbone and genetic modules composing the cassette are indicated. The cassette comprises: Prom-SD (P): rrn promoter along with a Shine-Dalgarno sequence (gray); *aadA*: spectinomycin resistance gene (yellow); 5'UTR: 5' untranslated region (blue); *smGFP*: gene encoding the soluble monomeric green fluorescent protein (green); and 3'UTR (T): 3' untranslated region (light gray). Location of primers used to check the presence of the full-length cassette inserted in between arms (black arrows), *trnI* arm (gray arrows) *trnA* arm (blue arrows), *KanR* (red arrows), *aadA* (yellow arrows) and *smGFP* (green arrows) are indicated. **(b)** PCRs using primers for the *trnA* and *trnI* homologous arms along with primers external of the dual-selection cassette were used to characterize the $_e$Gen1 plasmid extracted by back transformation into *E. coli*. Plasmids extracted from two independent colonies (1 and 2) are indicated. DNA bands of 4.3 and 2.5 kb at the same molecular weight of the positive control, Gen1, indicate the presence of full-length homologous arms in both $_e$Gen1 plasmids. On the contrary, the presence of lower-molecular weight bands of 0.46 kb rather than 2.8 kb (Gen1) indicate removal of the dual selection cassette in $_e$Gen1. **(c)** PCRs using primers for *smGFP* (0.72 kb), *aadA* (0.79 kb) and *KanR* gene (0.8 kb) confirmed the absence of the selection cassette and the presence of the backbone vector in $_e$Gen1. The Gen1, was used as positive control for comparison of the molecular weight of DNA bands. The negative controls (blanks) and DNA molecular markers (kb) are also indicate in the gels. These results have been confirmed by sequencing analysis of the entire $_e$Gen1 plasmid.

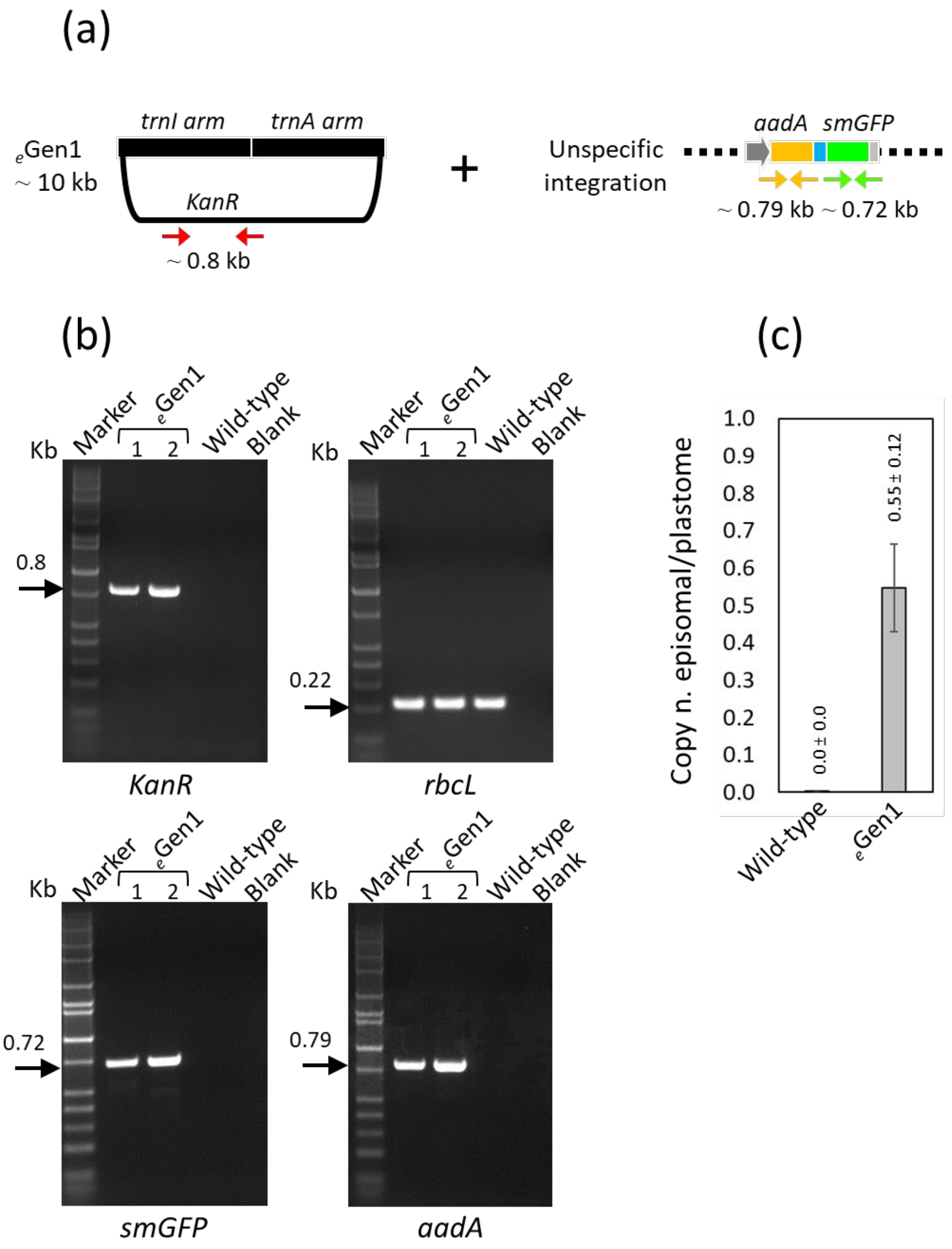

Figure S5: Determination of episome/plastome ratio of $_{e}$Gen1-containing lines at the 3rd round of tissue culture. (a) The vector rearrangement in plants containing $_{e}$Gen1 (~10 kb) leads the formation of the episome and unpredicted cassette integration. The *trnI/trnA* homologous arms, the kanamycin resistance gene (*KanR*) of the backbone and genetic modules of the selection cassette are indicated. The cassette comprises: Prom-SD (P): rrn promoter along with a Shine-Dalgarno sequence (gray); *aadA*: spectinomycin resistance gene (yellow); 5'UTR: 5' untranslated region (blue); *smGFP*: gene encoding the soluble monomeric green fluorescent protein (green); and 3'UTR (T): 3' untranslated region (light gray). Location of primers used to check *KanR* (red arrows), *aadA* (yellow arrows) and *smGFP* (green arrows) are indicated. **(b)** PCRs for *KanR* (0.8 kb), *rbcL* fragment (0.22 kb), s*mGFP* (0.72 kb), and *aadA* (0.79 kb) using DNA samples extracted from two independent $_{e}$Gen1-containing lines (1 and 2) at the third round of tissue culture. The second $_{e}$Gen1-containing line 2 was the line selected for further study (Figure S1). The PCR profiles confirmed that $_{e}$Gen1 was stable at this developmental stage. Wild-type controls, blanks and molecular markers (kb) have been included. **(c)** Graph summarizing the ratio of copy number of episome vs plastome (copy n. episomal/plastome) in genomic DNA preparations of the selected $_{e}$Gen1-containing line determined by qPCR analysis from leaf tissue of transgenic plants and wild-type controls at the third round of tissue culture. Results are expressed as mean ± standard deviation (sd) of a total of 3 biological and 4 technical replicates per each biological replicate.

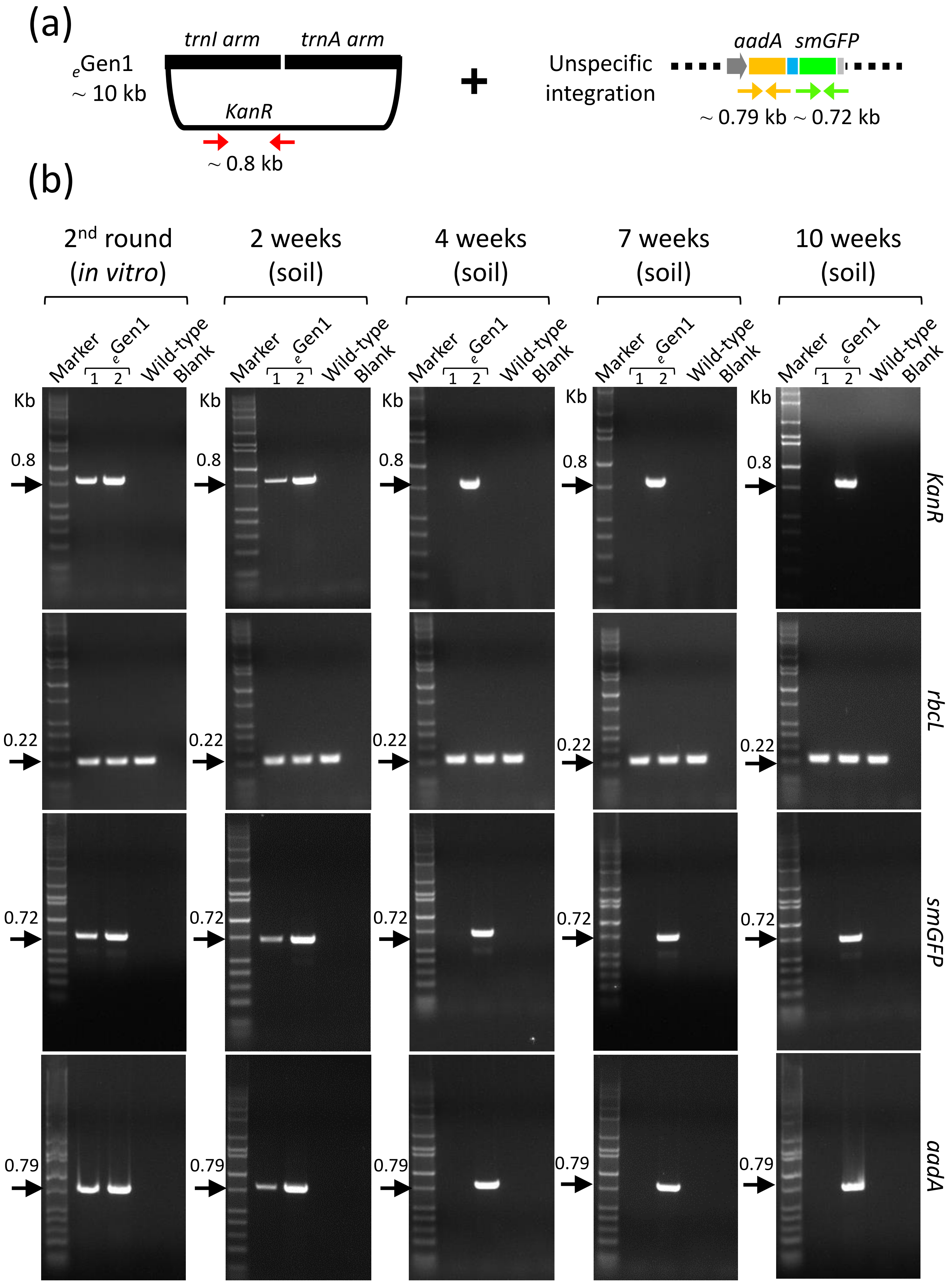

(a)
eGen1
~ 10 kb
trnI arm
trnA arm
KanR
~ 0.8 kb
+
Unspecific integration
aadA
smGFP
~ 0.79 kb
~ 0.72 kb
(b)
2nd round (in vitro)
2 weeks (soil)
4 weeks (soil)
7 weeks (soil)
10 weeks (soil)
Marker
eGen1
1
2
Wild-type
Blank
Kb
0.8
0.22
0.72
0.79
KanR
rbcL
smGFP
aadA

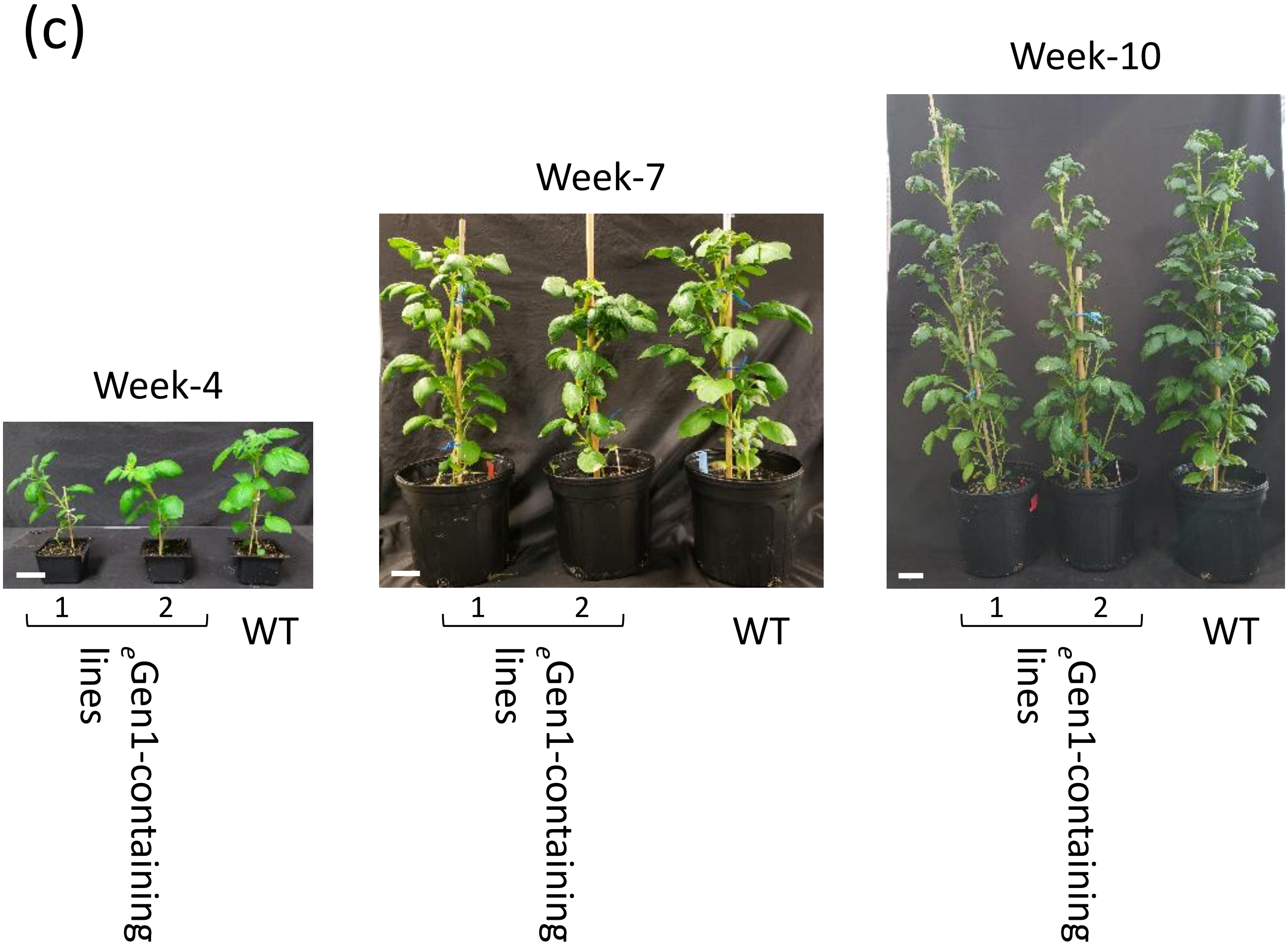

Figure S6: Stability of $_{e}$Gen1 episome at different plant developmental stages. (a) The vector rearrangement in $_{e}$Gen1-containing plants leads to the formation of the episome (~10 kb) and unpredicted cassette integration. The *trnI/trnA* homologous arms, the kanamycin resistance gene (*KanR*) of the backbone and genetic modules of the dual selection cassette are indicated. The cassette comprises: Prom-SD (P): rrn promoter along with a Shine-Dalgarno sequence (gray); *aadA*: spectinomycin resistance gene (yellow); 5’UTR: 5’ untranslated region (blue); *smGFP*: gene encoding the soluble monomeric green fluorescent protein (green); and 3’UTR (T): 3’ untranslated region (light gray). Location of primers used to check *KanR* (red arrows), *aadA* (yellow arrows) and *smGFP* (green arrows) are indicated. **(b)** PCRs for *KanR* (0.8 kb), *rbcL* fragment (0.22 kb), s*mGFP* (0.72 kb), and *aadA* (0.79 kb) using DNA samples extracted from $_{e}$Gen1-containing lines at the indicated developmental stages are shown. The PCR profiles confirmed that $_{e}$Gen1 (Figure S1) is stable throughout all plant developmental stages. Wild-type samples (WT), blanks and molecular markers (kb) have been included. **(c)** Representative plants at the second round of tissue culture (2nd round *in vitro*) grown on potting mix for 4, 7 and 10 weeks (anthesis); $_{e}$Gen1-containing lines along with wild-type control plants (WT). Scale bars = 5 cm.

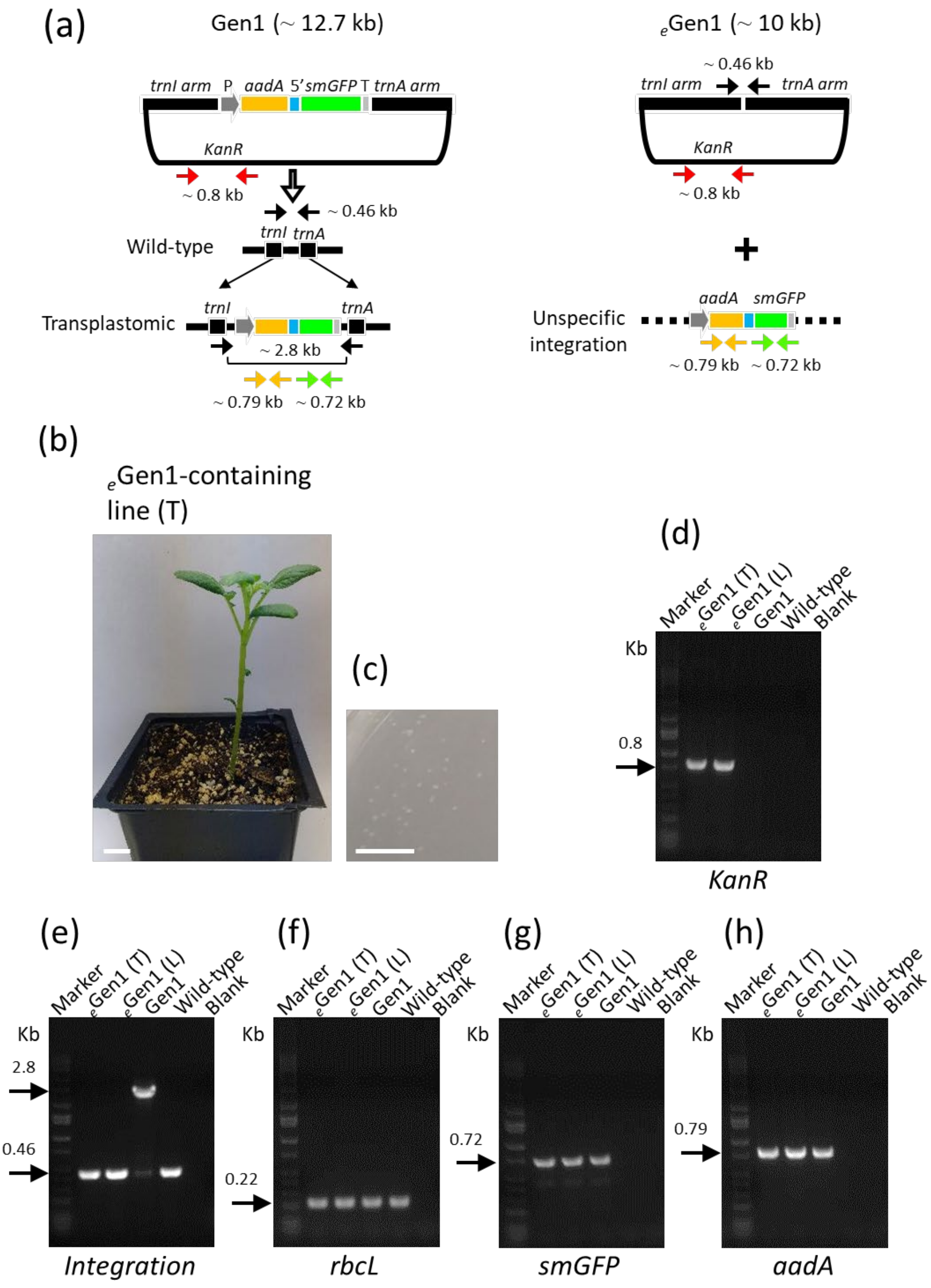

Figure S7: Characterization of $_{e}$Gen1-containing lines originated from tubers.
(a) Schematic representation of vector rearrangement in Gen1 (~12.7 kb) integrating and $_{e}$Gen1 (~10 kb) containing plants originated from tubes. In $_{e}$Gen1-containing lines the selection cassette is integrated in an unpredicted plastome site. The *trnI/trnA* homologous arms, the kanamycin resistance gene (*KanR*) of the backbone and a cassette modules are indicated. The dual selection cassette comprises: Prom-SD (P): rrn promoter along with a Shine-Dalgarno sequence (gray); *aadA*: spectinomycin resistance gene (yellow); 5'UTR: 5' untranslated region (blue); *smGFP*: gene encoding the soluble monomeric green fluorescent protein (green); and 3'UTR (T): 3' untranslated region (light gray). Location of primers used to check integration in the *trnI/trnA* region (black arrows), *KanR* (red arrows), *aadA* (yellow arrows) and *smGFP* (green arrows) are indicated. **(b)** A second generation $_{e}$Gen1-containing plant regenerated from tubers of $_{e}$Gen1-containing line 2 plants grown on potting mix (line 2, Figure S1). **(c)** Bacterial colonies transformed with $_{e}$Gen1 contained in leaf tissue is shown in b. **(d-h)** PCRs for detection of *KanR* (0.8 kb), integration (2.8 or 0.46 kb bands, for integration or not, respectively), *rbcL* fragment (0.22 kb), s*mGFP* (0.72 kb), and *aadA* (0.79 kb) using DNA samples extracted from $_{e}$Gen1-containing lines originated from tubers (T) or from tissue culture (L) are shown. A Gen1-integrating line along with wild-type controls, blanks and molecular markers (kb) are also shown. The PCR results confirmed the presence of $_{e}$Gen1 in plants originating from tubers. Scale bars = 10 mm (a-b).

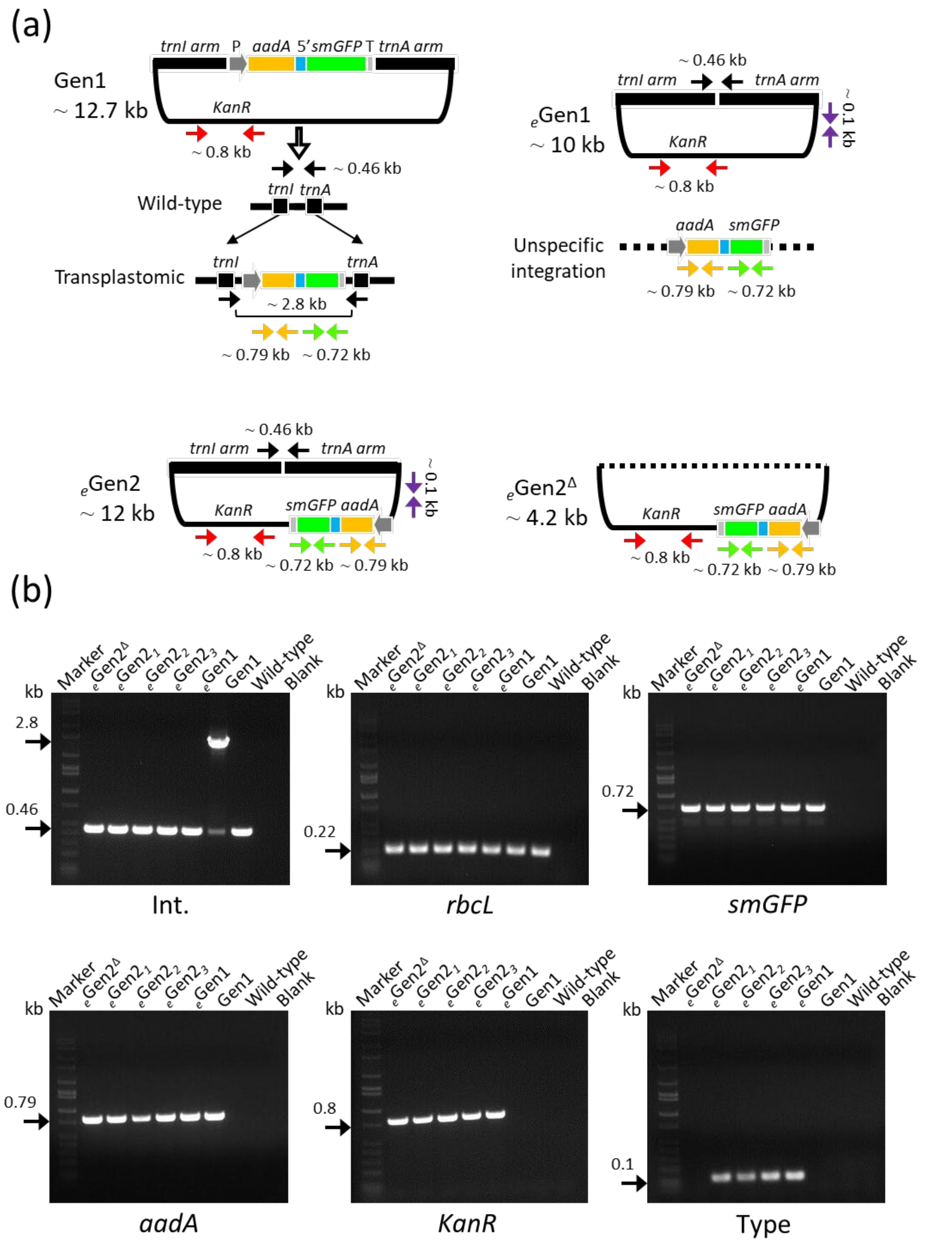

Figure S8: Characterization of synplastomic $_e$Gen2-containing lines. (a) Schematic representation of vector rearrangement in transgenic plants integrating Gen1 (~12.7 kb), along with plants containing either the episome $_e$Gen1 (~10 kb), $_e$Gen2 (~12 kb) or $_e$Gen2$^\Delta$ (~4.2 kb). In $_e$Gen1-containing line the selection cassette is integrated in an unpredicted plastome site, while in $_e$Gen2-containing lines the selection cassette is stably part of the episome backbone. The *trnI/trnA* homologous arms, the kanamycin resistance gene (*KanR*) of the backbone and genetic modules of the selection cassette are indicated. The cassette comprises: Prom-SD (P): rrn promoter along with a Shine-Dalgarno sequence (gray); *aadA*: spectinomycin resistance gene (yellow); 5'UTR: 5' untranslated region (blue); *smGFP*: gene encoding the soluble monomeric green fluorescent protein (green); and 3'UTR (T): 3' untranslated region (light gray). Location of primers used to check integration in the *trnI/trnA* region (black arrows), *KanR* (red arrows), *aadA* (yellow arrows), *smGFP* (green arrows) and an unique region of the full-length $_e$Gen2 episome (backbone type; purple arrows) are indicated. **(b)** PCR analysis of synplastomic $_e$Gen2-containing lines at the first cycle of tissue culture. DNA-bands of 2.8 kb indicate correct integration into the *trnI/trnA* plastome site in a Gen1-integrating plant used as positive control (Int.). Lower-molecular weight bands of 0.46 kb indicate wild-type IR regions of the plastome in all $_e$Gen2-containing lines. PCRs for *rbcL* fragment (0.22 kb), *smGFP* (0.72 kb), *aadA* (0.79 kb), *KanR* (0.8 kb) and backbone type (Type; 0.1 kb) to detect the presence of the full-length $_e$Gen2 are included. $_e$Gen2 ($_e$Gen2$_{1\text{-}3}$) or $_e$Gen2$^\Delta$-containing lines along with the two controls, $_e$Gen1-containing lines and Gen1-integrating lines are shown. Wild-type samples, blanks and molecular markers (kb) are also shown in the gels.

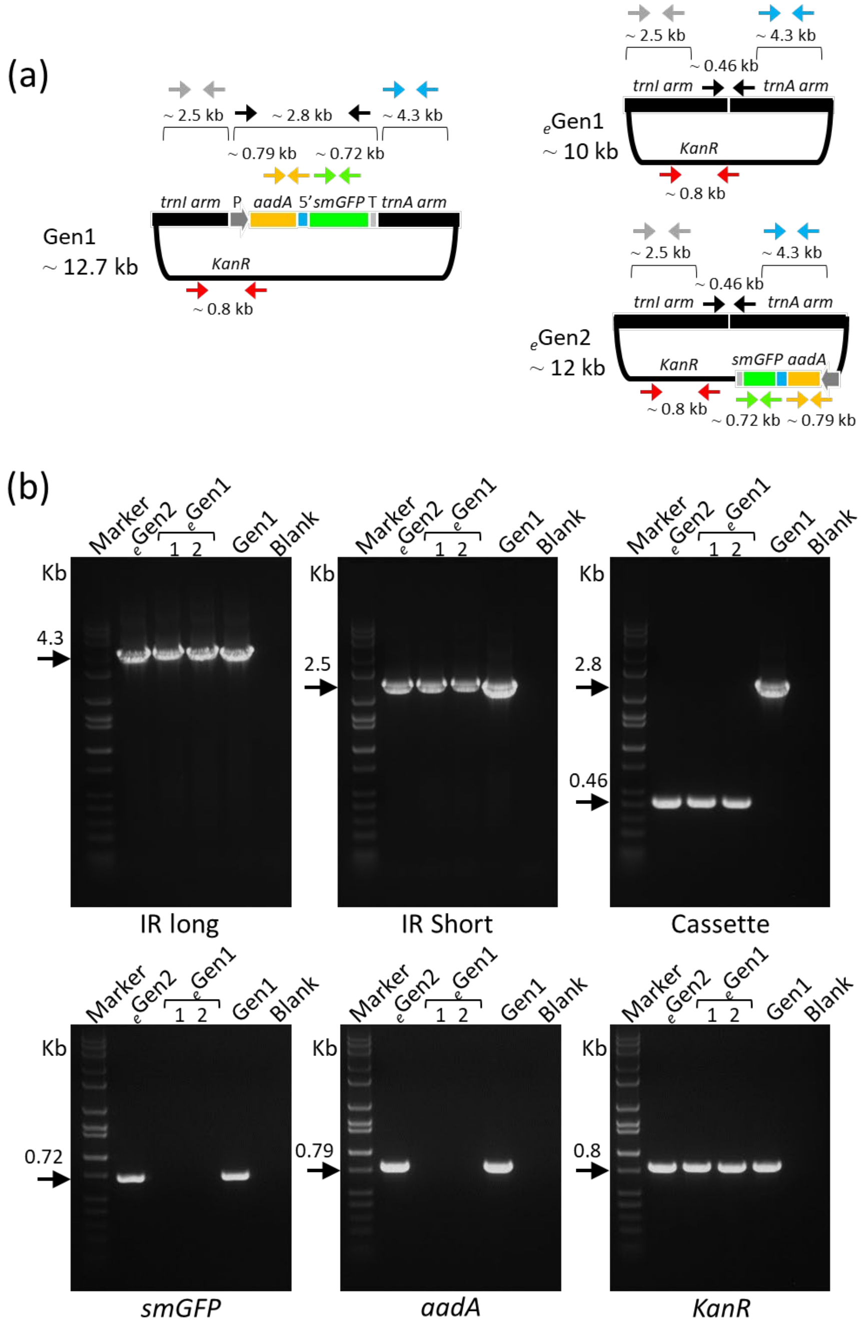

Figure S9: PCR characterization of $_e$Gen2 extracted from synplastomic plants. (a) Schematic representation of Gen1 (~12.7 kb), $_e$Gen1 (~10 kb) and $_e$Gen2 (~12 kb) plasmids. The *trnI/trnA* homologous arms, the kanamycin resistance gene (*KanR*) of the backbone and genetic modules forming the selection cassette are indicated. The cassette comprises: Prom-SD (P): rrn promoter along with a Shine-Dalgarno sequence (gray); *aadA*: spectinomycin resistance gene (yellow); 5'UTR: 5' untranslated region (blue); *smGFP*: gene encoding the soluble monomeric green fluorescent protein (green); and 3'UTR (T): 3' untranslated region (light gray). Location of primers used to check the presence of the full-length cassette inserted in between arms (black arrows), *trnI* arm (gray arrows) *trnA* arm (blue arrows), *KanR* (red arrows), *aadA* (yellow arrows) and *smGFP* (green arrows) are indicated. **(b)** PCRs for IR *trnI/trnA* homologous arms (short and long, respectively) along with the dual-selection cassette were used to characterize $_e$Gen2 extracted from synplastomic lines by back transformation into *E. coli*. PCR bands of 4.3 and 2.5 kb at the same molecular weight of the positive control, Gen1, indicate the presence of homologous arms. The presence of lower-molecular weight bands of 0.46 kb rather than 2.8 kb (Gen1) support correct transgene cassette location in the backbone. PCRs for *smGFP* (0.72 kb), *aadA* (0.79 kb) and *KanR* gene (0.8 kb) confirmed the presence of both the backbone and transgenes. Two bacterial colonies containing $_e$Gen1 and Gen1 were isolated for comparison. The negative controls (blanks) and DNA molecular markers (kb) are also indicate in the gels. These PCR results were confirmed by sequence analysis of the entire $_e$Gen2.

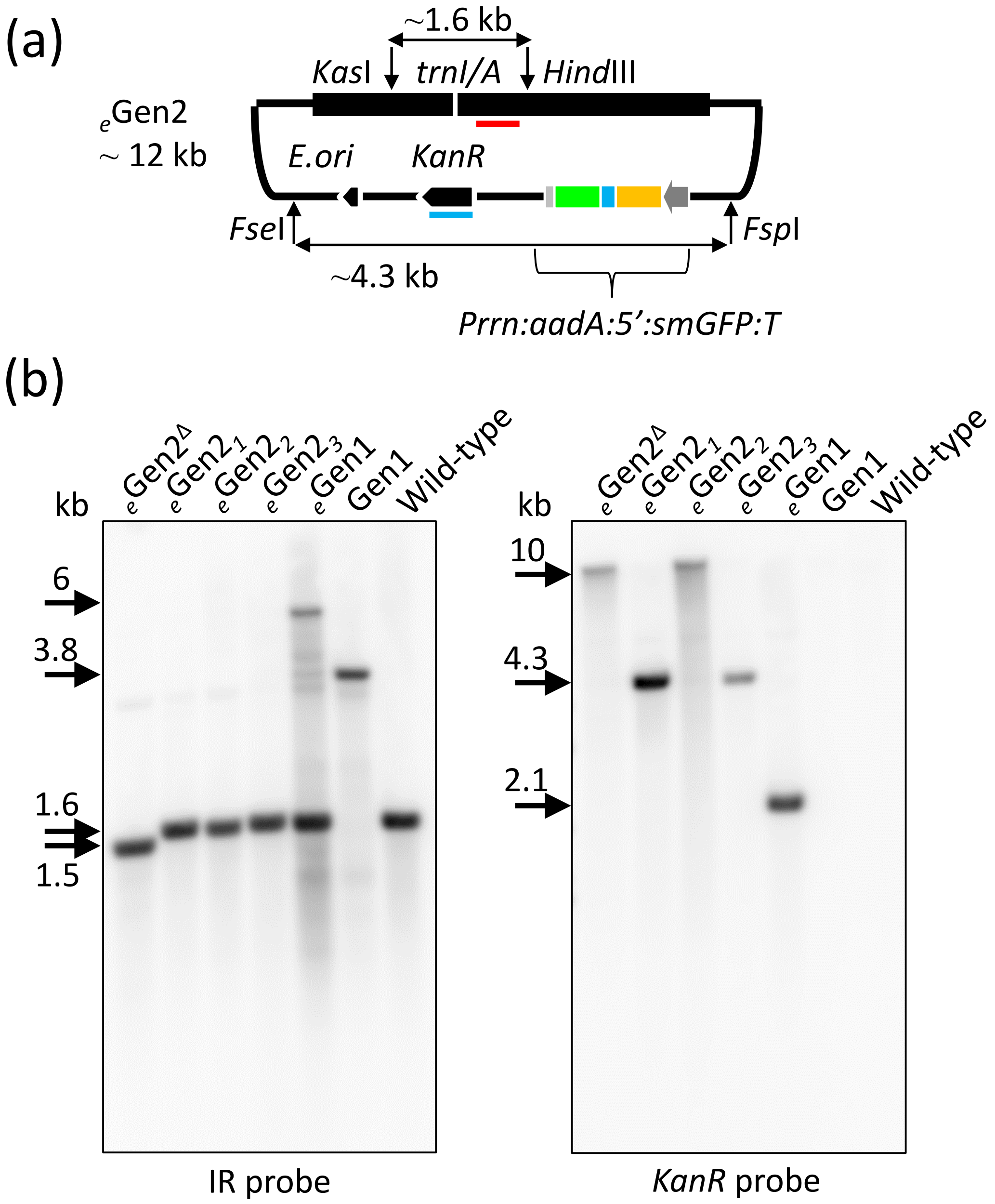

Figure S10: Southern blot profile of synplastomic $_e$Gen2-containing lines at the 2nd round of synplastomic plants. (a) Schematic representation of $_e$Gen2 (~12 kb) plasmids. The *trnI/trnA* homologous arms, the kanamycin resistance gene (*KanR*) of the backbone and genetic modules of the dual selection cassette are indicated. The cassette comprises: Prom-SD (P): rrn promoter along with a Shine-Dalgarno sequence (gray); *aadA*: spectinomycin resistance gene (yellow); 5'UTR: 5' untranslated region (blue); *smGFP*: gene encoding the soluble monomeric green fluorescent protein (green); and 3'UTR (T): 3' untranslated region (light gray). Location of restriction enzymes used for Southern blots, *Kas*I/*Hind*III and *Fse*I/*Fsp*I, and predicted size of DNA fragments are indicated. The *Kas*I/*Hind*III and *Fse*I/*Fsp*I fragments were detected by a ~0.5 kb probe designed on *trnI*/*trnA* (red bar) or *KanR* (blue bar), respectively. **(b)** Southern blot analysis performed using either an *IR* or *KanR* probe and leaf total DNA preparations extracted from $_e$Gen2-containing lines 1-3 and a line harboring $_e$Gen2$^{\Delta}$ at the second round of transplastomic lines. Genomic DNA samples from $_e$Gen1-containing and Gen1-integrating lines along with wild-type plants were used as a comparison. Molecular weight of DNA fragments (kb) are indicated in the blots.

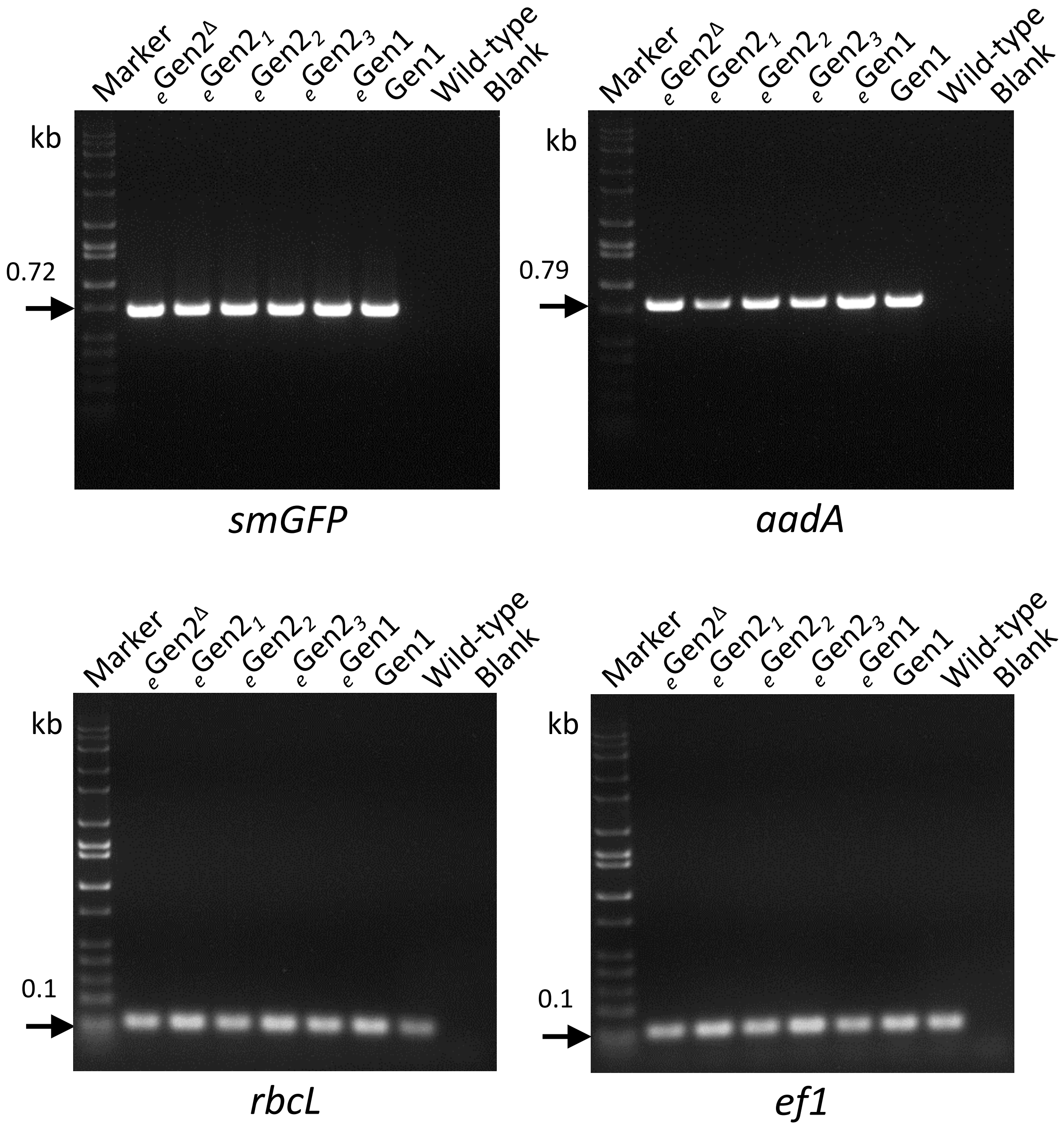

Figure S11: Transgene expression in $_{e}$Gen2-containing lines. RT-PCRs using cDNA preparations from $_{e}$Gen2$_{1\text{-}3}$ and $_{e}$Gen2$^{\Delta}$-containing lines. $_{e}$Gen1-containing and a Gen1-integrating line along with wild-type controls and blanks have been included. A pair of primers specific for s*mGFP* and *aadA* were used to detect the full-length cDNAs (0.72 and 0.79 kb, respectively). PCRs for *rbcL* and *ef1* genes were used as loading controls (0.1 kb DNA bands) for the plastome and nuclear genome. Molecular weight markers (kb) are shown in the gel.

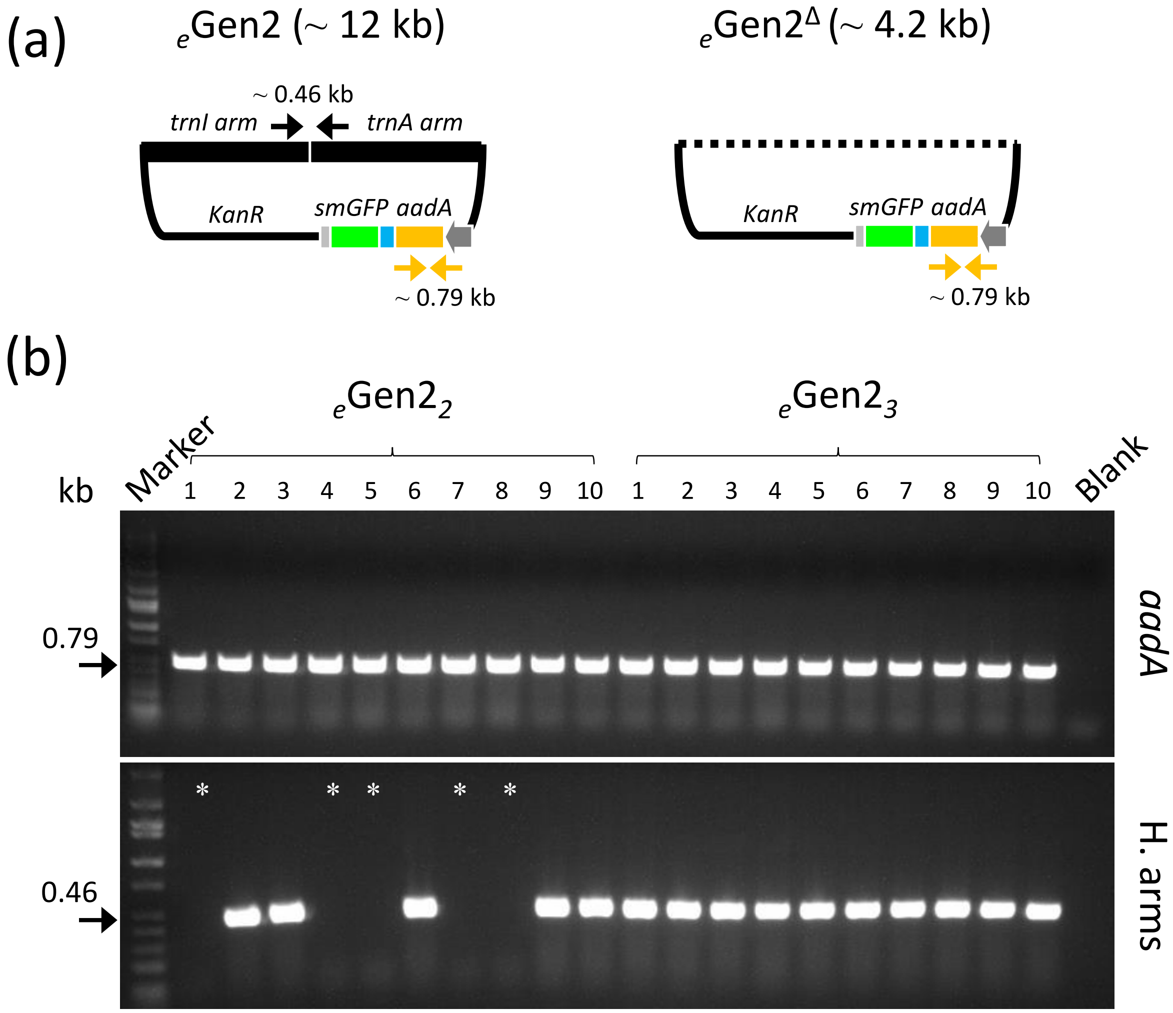

Figure S12: PCRs on bacteria colonies transformed with ${}_{e}$Gen2 contained in leaf tissue. (a) Schematic representation of ${}_{e}$Gen2 episome (~12 kb) and the ${}_{e}$Gen2$^{\Delta}$ short form (~4.2 kb) which contain a large deletion of the *trnI/trnA* homologous region (dotted line). In both ${}_{e}$Gen2 episomes the selection cassette is stably part of the episome backbone. The *trnI/trnA* homologous arms, the kanamycin resistance gene (*KanR*) of the backbone and genetic parts of the selection cassette are indicated. The cassette comprises: Prom-SD (P): rrn promoter along with a Shine-Dalgarno sequence (gray); *aadA*: spectinomycin resistance gene (yellow); 5'UTR: 5' untranslated region (blue); *smGFP*: gene encoding the soluble monomeric green fluorescent protein (green); and 3'UTR (T): 3' untranslated region (light gray). Location of primers used to check the presence of a *trnI/trnA internal* fragment (black arrows) and the *aadA* gene (yellow arrows) are indicated. **(b)** For each ${}_{e}$Gen2-containing line (${}_{e}$Gen2$_{2}$ or ${}_{e}$Gen2$_{3}$), a total of ten *E. coli* colonies (1-10) were tested. The presence of the ${}_{e}$Gen2 backbone in all bacterial samples was confirmed by PCR-positive products for the *aadA* gene (0.79 kb). The presence of the homologous region of full-length ${}_{e}$Gen2 was detected by using primers for an internal region of 0.46 kb (H. arms). Asterisk (*) indicate bacterial samples from ${}_{e}$Gen2-containing line 2, which contained the ${}_{e}$Gen2$^{\Delta}$ form (~50% of total colonies). Blanks and molecular markers (kb) are shown in the gels.

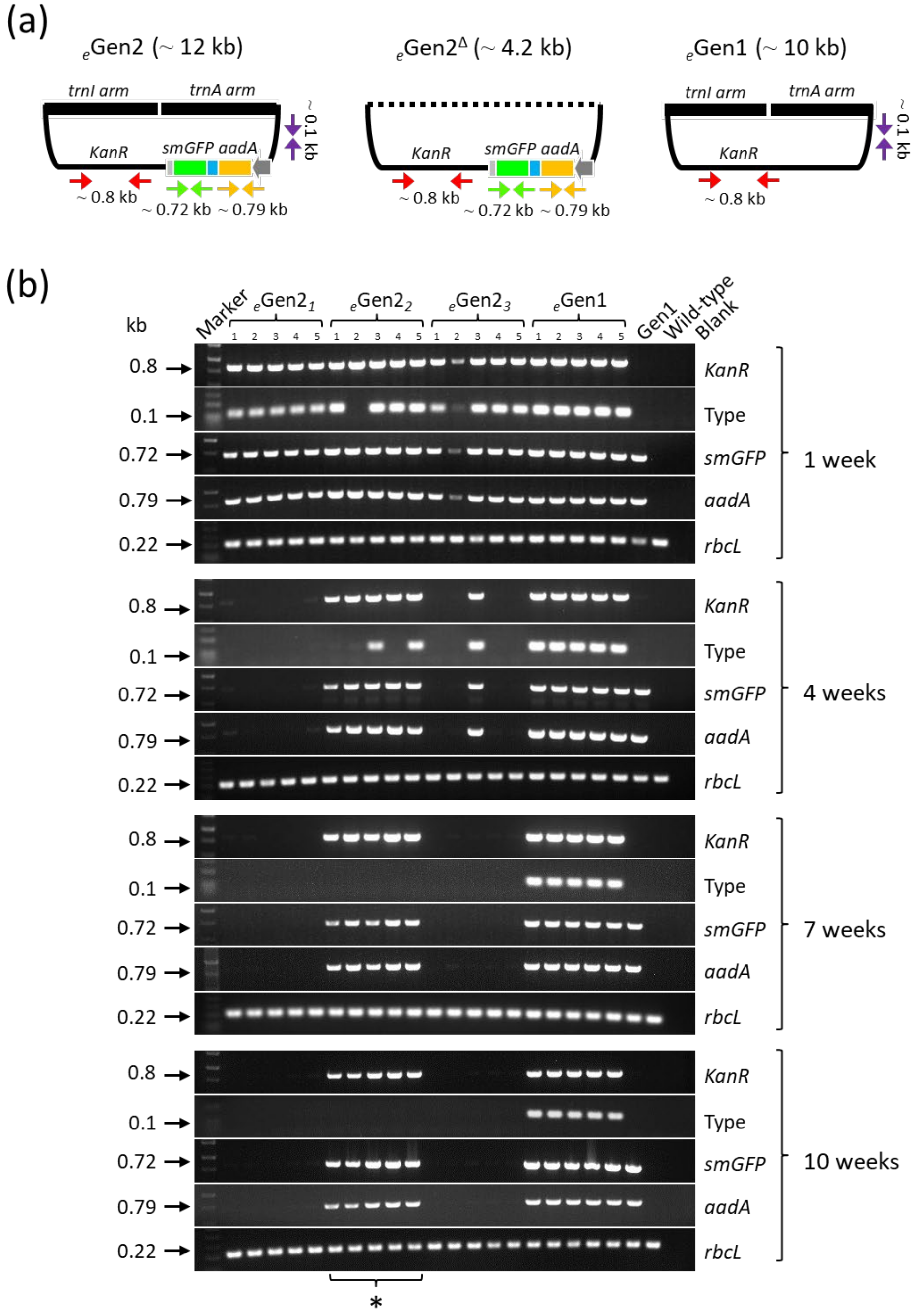

Figure S13: Stability of $_{e}$Gen2 at different plant developmental stages. (a) Schematic representation of $_{e}$Gen2 full-length (~12 kb), $_{e}$Gen2$^{\Delta}$ short-form (~4.2 kb) and $_{e}$Gen1 (~10 kb) episomes. In $_{e}$Gen1 the cassette is removed, while in both types of $_{e}$Gen2 episomes is stably part of the backbone. The *trnI/trnA* homologous arms, the kanamycin resistance gene (*KanR*) of the backbone and genetic modules composing the selection cassette are indicated. The cassette comprises: Prom-SD (P): rrn promoter along with a Shine-Dalgarno sequence (gray); *aadA*: spectinomycin resistance gene (yellow); 5'UTR: 5' untranslated region (blue); *smGFP*: gene encoding the soluble monomeric green fluorescent protein (green); and 3'UTR (T): 3' untranslated region (light gray). Location of primers used to check *KanR* (red arrows), *aadA* (yellow arrows) and *smGFP* (green arrows) genes, along with primers to check an unique region of the full-length $_{e}$Gen2 episome (backbone type; purple arrows) are indicated. **(b)** Molecular characterization of $_{e}$Gen2-containing lines at 1, 4, 7 and 10 (anthesis) weeks on potting mix. $_{e}$Gen2-containing lines ($_{e}$Gen2$_{1\text{-}3}$), along with $_{e}$Gen1-containing and the Gen1-integrating control lines are shown. Five plants (1-5) per each genotype have been used. Wild-type samples, blanks and molecular markers (kb) are also shown in the gels. PCRs for *KanR* (0.8 kb), backbone type (Type; 0.1 kb) to detect the presence of full-length $_{e}$Gen2, s*mGFP* (0.72 kb), *aadA* (0.79 kb) and *rbcL* fragment (0.22 kb) are included. Asterisk (*) indicate the line able to harbor $_{e}$Gen2$^{\Delta}$ until anthesis (10 weeks on potting mix).
