# Supplementary material for: Mini‐synplastomes for plastid genetic engineering
Source: Plant Biotechnol J. 2021 Oct 24;20(2):360–73. doi: 10.1111/pbi.13717 (PMC8753362; doi:10.1111/pbi.13717)
Supplement: Supplementary file 2 — Table S1 Multi‐sequence alignment of homologous regions. Table S2 Multi‐sequence alignment of trnI/trnA region of potato. Table S3 Primers used in this study. [file PBI-20-360-s002.pdf]

Table S1: Multi sequence alignment of homologous regions from: potato plastome (*Solanum tuberosum;* GenBank: DQ386163.2); tobacco plastome (*Nicotiana tabacum;* NCBI Reference Sequence: NC_001879.2); Gen1; $_{e}$Gen1; and $_{e}$Gen2. In the alignment are indicated: tobacco specific mutations (yellow); synthetic mutations (red); *oriA2* and *A1* (deep gray); *NICE* (light gray); the transgene cassette integration site is indicated with I. Multi sequence alignment obtained using Clustal Omega Software (EMBL-EBI).

```
Potato       AGCCCAATGTGAGTTTTTCTAGTTGGATTTGCTCCCCCGCCGTCGTTCAATGAGAATGGA   60
Tobacco      AGCCCAATGTGAGTTTTTCTAGTTGGATTTGCTCCCCCGCCGTCGTTCAATGAGAATGGA   60
Gen1         AGCCCAATGTGAGTTTTTCTAGTTGGATTTGCTCCCCCGCCGTCGTTCAATGAGAATGGA   60
eGen1        AGCCCAATGTGAGTTTTTCTAGTTGGATTTGCTCCCCCGCCGTCGTTCAATGAGAATGGA   60
eGen2        AGCCCAATGTGAGTTTTTCTAGTTGGATTTGCTCCCCCGCCGTCGTTCAATGAGAATGGA   60
             ************************************************************

                                                   1
Potato       TAAGAGGCTCGTGGGATTGACGTGAGGGGGCAGGGATGACTATATTTCTGGGAGCGAACT   120
Tobacco      TAAGAGGCTCGTGGGATTGACGTGAGGGGGCAGGGATGGCTATATTTCTGGGAGCGAACT   120
Gen1         TAAGAGGCTCGTGGGATTGACGTGAGGGGGCAGGGATGGCTATATTTCTGGGAGCGAACT   120
eGen1        TAAGAGGCTCGTGGGATTGACGTGAGGGGGCAGGGATGGCTATATTTCTGGGAGCGAACT   120
eGen2        TAAGAGGCTCGTGGGATTGACGTGAGGGGGCAGGGATGGCTATATTTCTGGGAGCGAACT   120
             **************************************.*********************

Potato       CCGGGCGAATATGAAGCGCATGGATACAAGTTATGCCTTGGAATGAAAGACAATTCCGAA   180
Tobacco      CCGGGCGAATATGAAGCGCATGGATACAAGTTATGCCTTGGAATGAAAGACAATTCCGAA   180
Gen1         CCGGGCGAATATGAAGCGCATGGATACAAGTTATGCCTTGGAATGAAAGACAATTCCGAA   180
eGen1        CCGGGCGAATATGAAGCGCATGGATACAAGTTATGCCTTGGAATGAAAGACAATTCCGAA   180
eGen2        CCGGGCGAATATGAAGCGCATGGATACAAGTTATGCCTTGGAATGAAAGACAATTCCGAA   180
             ************************************************************

Potato       TCCGCTTTGTCTACGAACAAGGAAGCTATAAGTAATGCAACTATGAATCTCATGGAGAGT   240
Tobacco      TCCGCTTTGTCTACGAACAAGGAAGCTATAAGTAATGCAACTATGAATCTCATGGAGAGT   240
Gen1         TCCGCTTTGTCTACGAACAAGGAAGCTATAAGTAATGCAACTATGAATCTCATGGAGAGT   240
eGen1        TCCGCTTTGTCTACGAACAAGGAAGCTATAAGTAATGCAACTATGAATCTCATGGAGAGT   240
eGen2        TCCGCTTTGTCTACGAACAAGGAAGCTATAAGTAATGCAACTATGAATCTCATGGAGAGT   240
             ************************************************************

                                                                       2
Potato       TCGATCCTGGCTCAGGATGAACGCTGGCGGCATGCTTAACACATGCAAGTCGGACGGGAA   300
Tobacco      TCGATCCTGGCTCAGGATGAACGCTGGCGGCATGCTTAACACATGCAAGTCGGACGGG--   298
Gen1         TCGATCCTGGCTCAGGATGAACGCTGGCGGCATGCTTAACACATGCAAGTCGGACGGG--   298
eGen1        TCGATCCTGGCTCAGGATGAACGCTGGCGGCATGCTTAACACATGCAAGTCGGACGGGAA   300
eGen2        TCGATCCTGGCTCAGGATGAACGCTGGCGGCATGCTTAACACATGCAAGTCGGACGGGAA   300
             **********************************************************

Potato       ACACGGGAAACGGTGTTTCCAGTGGCGGACGGGTGAGTAACGCGTAAGAACCTGCCCTTG   360
Tobacco      -------AAGTGGTGTTTCCAGTGGCGGACGGGTGAGTAACGCGTAAGAACCTGCCCTTG   351
Gen1         -------AAGTGGTGTTTCCAGTGGCGGACGGGTGAGTAACGCGTAAGAACCTGCCCTTG   351
eGen1        ACACGGGAAACGGTGTTTCCAGTGGCGGACGGGTGAGTAACGCGTAAGAACCTGCCCTTG   360
eGen2        ACACGGGAAACGGTGTTTCCAGTGGCGGACGGGTGAGTAACGCGTAAGAACCTGCCCTTG   360
                    **. *************************************************

Potato       GGAGGGGAACAACAGCTGGAAACGGCTGCTAATACCCCGTAGGCTGAGGAGCAAAAGGAG   420
Tobacco      GGAGGGGAACAACAGCTGGAAACGGCTGCTAATACCCCGTAGGCTGAGGAGCAAAAGGAG   411
Gen1         GGAGGGGAACAACAGCTGGAAACGGCTGCTAATACCCCGTAGGCTGAGGAGCAAAAGGAG   411
eGen1        GGAGGGGAACAACAGCTGGAAACGGCTGCTAATACCCCGTAGGCTGAGGAGCAAAAGGAG   420
eGen2        GGAGGGGAACAACAGCTGGAAACGGCTGCTAATACCCCGTAGGCTGAGGAGCAAAAGGAG   420
             ************************************************************

Potato       GAATCCGCCCGAGGAGGGGCTCGCGTCTGATTAGCTAGTTGGTGAGGCAATAGCTTACCA   480
Tobacco      GAATCCGCCCGAGGAGGGGCTCGCGTCTGATTAGCTAGTTGGTGAGGCAATAGCTTACCA   471
Gen1         GAATCCGCCCGAGGAGGGGCTCGCGTCTGATTAGCTAGTTGGTGAGGCAATAGCTTACCA   471
```

```
eGen1        GAATCCGCCCGAGGAGGGGCTCGCGTCTGATTAGCTAGTTGGTGAGGCAATAGCTTACCA    480
eGen2        GAATCCGCCCGAGGAGGGGCTCGCGTCTGATTAGCTAGTTGGTGAGGCAATAGCTTACCA    480
             ************************************************************

Potato       AGGCGATGATCAGTAGCTGGTCCGAGAGGATGATCAGCCACACTGGGACTGAGACACGGC    540
Tobacco      AGGCGATGATCAGTAGCTGGTCCGAGAGGATGATCAGCCACACTGGGACTGAGACACGGC    531
Gen1         AGGCGATGATCAGTAGCTGGTCCGAGAGGATGATCAGCCACACTGGGACTGAGACACGGC    531
eGen1        AGGCGATGATCAGTAGCTGGTCCGAGAGGATGATCAGCCACACTGGGACTGAGACACGGC    540
eGen2        AGGCGATGATCAGTAGCTGGTCCGAGAGGATGATCAGCCACACTGGGACTGAGACACGGC    540
             ************************************************************

Potato       CCAGACTCCTACGGGAGGCAGCAGTGGGGAATTTTCCGCAATGGGCGAAAGCCTGACGGA    600
Tobacco      CCAGACTCCTACGGGAGGCAGCAGTGGGGAATTTTCCGCAATGGGCGAAAGCCTGACGGA    591
Gen1         CCAGACTCCTACGGGAGGCAGCAGTGGGGAATTTTCCGCAATGGGCGAAAGCCTGACGGA    591
eGen1        CCAGACTCCTACGGGAGGCAGCAGTGGGGAATTTTCCGCAATGGGCGAAAGCCTGACGGA    600
eGen2        CCAGACTCCTACGGGAGGCAGCAGTGGGGAATTTTCCGCAATGGGCGAAAGCCTGACGGA    600
             ************************************************************

Potato       GCAATGCCGCGTGGAGGTAGAAGGCCCACGGGTCGTGAACTTCTTTTCCCGGAGAAGAAG    660
Tobacco      GCAATGCCGCGTGGAGGTAGAAGGCCCACGGGTCGTGAACTTCTTTTCCCGGAGAAGAAG    651
Gen1         GCAATGCCGCGTGGAGGTAGAAGGCCCACGGGTCGTGAACTTCTTTTCCCGGAGAAGAAG    651
eGen1        GCAATGCCGCGTGGAGGTAGAAGGCCCACGGGTCGTGAACTTCTTTTCCCGGAGAAGAAG    660
eGen2        GCAATGCCGCGTGGAGGTAGAAGGCCCACGGGTCGTGAACTTCTTTTCCCGGAGAAGAAG    660
             ************************************************************

Potato       CAATGACGGTATCTGGGGAATAAGCATCGGCTAACTCTGTGCCAGCAGCCGCGGTAATAC    720
Tobacco      CAATGACGGTATCTGGGGAATAAGCATCGGCTAACTCTGTGCCAGCAGCCGCGGTAATAC    711
Gen1         CAATGACGGTATCTGGGGAATAAGCATCGGCTAACTCTGTGCCAGCAGCCGCGGTAATAC    711
eGen1        CAATGACGGTATCTGGGGAATAAGCATCGGCTAACTCTGTGCCAGCAGCCGCGGTAATAC    720
eGen2        CAATGACGGTATCTGGGGAATAAGCATCGGCTAACTCTGTGCCAGCAGCCGCGGTAATAC    720
             ************************************************************

Potato       AGAGGATGCAAGCGTTATCCGGAATGATTGGGCGTAAAGCGTCTGTAGGTGGCTTTTTAA    780
Tobacco      AGAGGATGCAAGCGTTATCCGGAATGATTGGGCGTAAAGCGTCTGTAGGTGGCTTTTTAA    771
Gen1         AGAGGATGCAAGCGTTATCCGGAATGATTGGGCGTAAAGCGTCTGTAGGTGGCTTTTTAA    771
eGen1        AGAGGATGCAAGCGTTATCCGGAATGATTGGGCGTAAAGCGTCTGTAGGTGGCTTTTTAA    780
eGen2        AGAGGATGCAAGCGTTATCCGGAATGATTGGGCGTAAAGCGTCTGTAGGTGGCTTTTTAA    780
             ************************************************************

Potato       GTCCGCCGTCAAATCCCAGGGCTCAACCCTGGACAGGCGGTGGAAACTACCAAGCTGGAG    840
Tobacco      GTCCGCCGTCAAATCCCAGGGCTCAACCCTGGACAGGCGGTGGAAACTACCAAGCTGGAG    831
Gen1         GTCCGCCGTCAAATCCCAGGGCTCAACCCTGGACAGGCGGTGGAAACTACCAAGCTGGAG    831
eGen1        GTCCGCCGTCAAATCCCAGGGCTCAACCCTGGACAGGCGGTGGAAACTACCAAGCTGGAG    840
eGen2        GTCCGCCGTCAAATCCCAGGGCTCAACCCTGGACAGGCGGTGGAAACTACCAAGCTGGAG    840
             ************************************************************

Potato       TACGGTAGGGGCAGAGGGAATTTCCGGTGGAGCGGTGAAATGCGTAGAGATCGGAAAGAA    900
Tobacco      TACGGTAGGGGCAGAGGGAATTTCCGGTGGAGCGGTGAAATGCGTAGAGATCGGAAAGAA    891
Gen1         TACGGTAGGGGCAGAGGGAATTTCCGGTGGAGCGGTGAAATGCGTAGAGATCGGAAAGAA    891
eGen1        TACGGTAGGGGCAGAGGGAATTTCCGGTGGAGCGGTGAAATGCGTAGAGATCGGAAAGAA    900
eGen2        TACGGTAGGGGCAGAGGGAATTTCCGGTGGAGCGGTGAAATGCGTAGAGATCGGAAAGAA    900
             ************************************************************
                                                          3
Potato       CACCAACGGCGAAAGCACTCTGCTGGGCCGACACTGACACTGAGAGACGAAAGCTAGGGG    960
Tobacco      CACCAACGGCGAAAGCACTCTGCTGGGCCGACACTGACACTGAGAGACGAAAGCTAGGGG    951
Gen1         CACCAACGGCGAAAGCACTCTGCTGGGCCGACACTGACACTGAGACACGAAAGCTAGGGG    951
eGen1        CACCAACGGCGAAAGCACTCTGCTGGGCCGACACTGACACTGAGACACGAAAGCTAGGGG    960
eGen2        CACCAACGGCGAAAGCACTCTGCTGGGCCGACACTGACACTGAGAGACGAAAGCTAGGGG    960
             ********************************************* **************

Potato       AGCGAATGGGATTAGATACCCCAGTAGTCCTAGCCGTAAACGATGGATACTAGGCGCTGT    1020
Tobacco      AGCGAATGGGATTAGATACCCCAGTAGTCCTAGCCGTAAACGATGGATACTAGGCGCTGT    1011
Gen1         AGCGAATGGGATTAGATACCCCAGTAGTCCTAGCCGTAAACGATGGATACTAGGCGCTGT    1011
```

```
eGen1      AGCGAATGGGATTAGATACCCCAGTAGTCCTAGCCGTAAACGATGGATACTAGGCGCTGT   1020
eGen2      AGCGAATGGGATTAGATACCCCAGTAGTCCTAGCCGTAAACGATGGATACTAGGCGCTGT   1020
           ************************************************************

Potato     GCGTATCGACCCGTGCAGTGCTGTAGCTAACGCGTTAAGTATCCCGCCTGGGGAGTACGT   1080
Tobacco    GCGTATCGACCCGTGCAGTGCTGTAGCTAACGCGTTAAGTATCCCGCCTGGGGAGTACGT   1071
Gen1       GCGTATCGACCCGTGCAGTGCTGTAGCTAACGCGTTAAGTATCCCGCCTGGGGAGTACGT   1071
eGen1      GCGTATCGACCCGTGCAGTGCTGTAGCTAACGCGTTAAGTATCCCGCCTGGGGAGTACGT   1080
eGen2      GCGTATCGACCCGTGCAGTGCTGTAGCTAACGCGTTAAGTATCCCGCCTGGGGAGTACGT   1080
           ************************************************************

Potato     TCGCAAGAATGAAACTCAAAGGAATTGACGGGGGCCCGCACAAGCGGTGGAGCATGTGGT   1140
Tobacco    TCGCAAGAATGAAACTCAAAGGAATTGACGGGGGCCCGCACAAGCGGTGGAGCATGTGGT   1131
Gen1       TCGCAAGAATGAAACTCAAAGGAATTGACGGGGGCCCGCACAAGCGGTGGAGCATGTGGT   1131
eGen1      TCGCAAGAATGAAACTCAAAGGAATTGACGGGGGCCCGCACAAGCGGTGGAGCATGTGGT   1140
eGen2      TCGCAAGAATGAAACTCAAAGGAATTGACGGGGGCCCGCACAAGCGGTGGAGCATGTGGT   1140
           ************************************************************

Potato     TTAATTCGATGCAAAGCGAAGAACCTTACCAGGGCTTGACATGCCGCGAATCCTCTTGAA   1200
Tobacco    TTAATTCGATGCAAAGCGAAGAACCTTACCAGGGCTTGACATGCCGCGAATCCTCTTGAA   1191
Gen1       TTAATTCGATGCAAAGCGAAGAACCTTACCAGGGCTTGACATGCCGCGAATCCTCTTGAA   1191
eGen1      TTAATTCGATGCAAAGCGAAGAACCTTACCAGGGCTTGACATGCCGCGAATCCTCTTGAA   1200
eGen2      TTAATTCGATGCAAAGCGAAGAACCTTACCAGGGCTTGACATGCCGCGAATCCTCTTGAA   1200
           ************************************************************

Potato     AGAGAGGGGTGCCTTCGGGAACGCGGACACAGGTGGTGCATGGCTGTCGTCAGCTCGTGC   1260
Tobacco    AGAGAGGGGTGCCTTCGGGAACGCGGACACAGGTGGTGCATGGCTGTCGTCAGCTCGTGC   1251
Gen1       AGAGAGGGGTGCCTTCGGGAACGCGGACACAGGTGGTGCATGGCTGTCGTCAGCTCGTGC   1251
eGen1      AGAGAGGGGTGCCTTCGGGAACGCGGACACAGGTGGTGCATGGCTGTCGTCAGCTCGTGC   1260
eGen2      AGAGAGGGGTGCCTTCGGGAACGCGGACACAGGTGGTGCATGGCTGTCGTCAGCTCGTGC   1260
           ************************************************************

Potato     CGTAAGGTGTTGGGTTAAGTCCCGCAACGAGCGCAACCCTCGTGTTTAGTTGCCATCGTT   1320
Tobacco    CGTAAGGTGTTGGGTTAAGTCCCGCAACGAGCGCAACCCTCGTGTTTAGTTGCCATCGTT   1311
Gen1       CGTAAGGTGTTGGGTTAAGTCCCGCAACGAGCGCAACCCTCGTGTTTAGTTGCCATCGTT   1311
eGen1      CGTAAGGTGTTGGGTTAAGTCCCGCAACGAGCGCAACCCTCGTGTTTAGTTGCCATCGTT   1320
eGen2      CGTAAGGTGTTGGGTTAAGTCCCGCAACGAGCGCAACCCTCGTGTTTAGTTGCCATCGTT   1320
           ************************************************************

Potato     GAGTTTGGAACCCTGAACAGACTGCCGGTGATAAGCCGGAGGAAGGTGAGGATGACGTCA   1380
Tobacco    GAGTTTGGAACCCTGAACAGACTGCCGGTGATAAGCCGGAGGAAGGTGAGGATGACGTCA   1371
Gen1       GAGTTTGGAACCCTGAACAGACTGCCGGTGATAAGCCGGAGGAAGGTGAGGATGACGTCA   1371
eGen1      GAGTTTGGAACCCTGAACAGACTGCCGGTGATAAGCCGGAGGAAGGTGAGGATGACGTCA   1380
eGen2      GAGTTTGGAACCCTGAACAGACTGCCGGTGATAAGCCGGAGGAAGGTGAGGATGACGTCA   1380
           ************************************************************

Potato     AGTCATCATGCCCCTTATGCCCTGGGCGACACACGTGCTACAATGGCCGGGACAAAGGGT   1440
Tobacco    AGTCATCATGCCCCTTATGCCCTGGGCGACACACGTGCTACAATGGCCGGGACAAAGGGT   1431
Gen1       AGTCATCATGCCCCTTATGCCCTGGGCGACACACGTGCTACAATGGCCGGGACAAAGGGT   1431
eGen1      AGTCATCATGCCCCTTATGCCCTGGGCGACACACGTGCTACAATGGCCGGGACAAAGGGT   1440
eGen2      AGTCATCATGCCCCTTATGCCCTGGGCGACACACGTGCTACAATGGCCGGGACAAAGGGT   1440
           ************************************************************

Potato     CGCGATCCCGCGAGGGTGAGCTAACCCCAAAAACCCGTCCTCAGTTCGGATTGCAGGCTG   1500
Tobacco    CGCGATCCCGCGAGGGTGAGCTAACCCCAAAAACCCGTCCTCAGTTCGGATTGCAGGCTG   1491
Gen1       CGCGATCCCGCGAGGGTGAGCTAACCCCAAAAACCCGTCCTCAGTTCGGATTGCAGGCTG   1491
eGen1      CGCGATCCCGCGAGGGTGAGCTAACCCCAAAAACCCGTCCTCAGTTCGGATTGCAGGCTG   1500
eGen2      CGCGATCCCGCGAGGGTGAGCTAACCCCAAAAACCCGTCCTCAGTTCGGATTGCAGGCTG   1500
           ************************************************************

Potato     CAACTCGCCTGCATGAAGCCGGAATCGCTAGTAATCGCCGGTCAGCCATACGGCGGTGAA   1560
Tobacco    CAACTCGCCTGCATGAAGCCGGAATCGCTAGTAATCGCCGGTCAGCCATACGGCGGTGAA   1551
Gen1       CAACTCGCCTGCATGAAGCCGGAATCGCTAGTAATCGCCGGTCAGCCATACGGCGGTGAA   1551
```

```
eGen1      CAACTCGCCTGCATGAAGCCGGAATCGCTAGTAATCGCCGGTCAGCCATACGGCGGTGAA   1560
eGen2      CAACTCGCCTGCATGAAGCCGGAATCGCTAGTAATCGCCGGTCAGCCATACGGCGGTGAA   1560
           ************************************************************

Potato     TTCGTTCCCGGGCCTTGTACACACCGCCCGTCACACTATGGGAGCTGGCCATGCCCGAAG   1620
Tobacco    TTCGTTCCCGGGCCTTGTACACACCGCCCGTCACACTATGGGAGCTGGCCATGCCCGAAG   1611
Gen1       TTCGTTCCCGGGCCTTGTACACACCGCCCGTCACACTATGGGAGCTGGCCATGCCCGAAG   1611
eGen1      TTCGTTCCCGGGCCTTGTACACACCGCCCGTCACACTATGGGAGCTGGCCATGCCCGAAG   1620
eGen2      TTCGTTCCCGGGCCTTGTACACACCGCCCGTCACACTATGGGAGCTGGCCATGCCCGAAG   1620
           ************************************************************

Potato     TCGTTACCTTAACCGCAAGGAGGGGGATGCCGAAGGCAGGGCTAGTGACTGGAGTGAAGT   1680
Tobacco    TCGTTACCTTAACCGCAAGGAGGGGGATGCCGAAGGCAGGGCTAGTGACTGGAGTGAAGT   1671
Gen1       TCGTTACCTTAACCGCAAGGAGGGGGATGCCGAAGGCAGGGCTAGTGACTGGAGTGAAGT   1671
eGen1      TCGTTACCTTAACCGCAAGGAGGGGGATGCCGAAGGCAGGGCTAGTGACTGGAGTGAAGT   1680
eGen2      TCGTTACCTTAACCGCAAGGAGGGGGATGCCGAAGGCAGGGCTAGTGACTGGAGTGAAGT   1680
           ************************************************************

Potato     CGTAACAAGGTAGCCGTACTGGAAGGTGCGGCTGGATCACCTCCTTTTCAGGGAGAGCTA   1740
Tobacco    CGTAACAAGGTAGCCGTACTGGAAGGTGCGGCTGGATCACCTCCTTTTCAGGGAGAGCTA   1731
Gen1       CGTAACAAGGTAGCCGTACTGGAAGGTGCGGCTGGATCACCTCCTTTTCAGGGAGAGCTA   1731
eGen1      CGTAACAAGGTAGCCGTACTGGAAGGTGCGGCTGGATCACCTCCTTTTCAGGGAGAGCTA   1740
eGen2      CGTAACAAGGTAGCCGTACTGGAAGGTGCGGCTGGATCACCTCCTTTTCAGGGAGAGCTA   1740
           ************************************************************

Potato     ATGCTTGTTGGGTATTTTGGTTTGACACTGCTTCACACCCCCAAAAAAAAGAAGGGAGCT   1800
Tobacco    ATGCTTGTTGGGTATTTTGGTTTGACACTGCTTCACACCCCCAAAAAAAAGAAGGGAGCT   1791
Gen1       ATGCTTGTTGGGTATTTTGGTTTGACACTGCTTCACACCCCCAAAAAAAAGAAGGGAGCT   1791
eGen1      ATGCTTGTTGGGTATTTTGGTTTGACACTGCTTCACACCCCCAAAAAAAAGAAGGGAGCT   1800
eGen2      ATGCTTGTTGGGTATTTTGGTTTGACACTGCTTCACACCCCCAAAAAAAAGAAGGGAGCT   1800
           ************************************************************
                                         4
Potato     ACGTCTGAGTTAAACTTGGAGATGGAAGTCTTCTTTCCTTTCTCGACGGTGAAGTAAGAC   1860
Tobacco    ACGTCTGAGTTAAACTTGGAGATGGAAGTCTTCTTTCCTTTCTCGACGGTGAAGTAAGAC   1851
Gen1       ACGTCTGAGTTAAACTTGGAGATGGAAGTCATCTTTCCTTTCTCGACGGTGAAGTAAGAC   1851
eGen1      ACGTCTGAGTTAAACTTGGAGATGGAAGTCATCTTTCCTTTCTCGACGGTGAAGTAAGAC   1860
eGen2      ACGTCTGAGTTAAACTTGGAGATGGAAGTCTTCTTTCCTTTCTCGACGGTGAAGTAAGAC   1860
           ******************************:*****************************
                                                        5
Potato     CAAGCTCATGAGCTTATTATCCTAGGTCGGAACAAGTTGATAGGATCCCCTTTTTTACGT   1920
Tobacco    CAAGCTCATGAGCTTATTATCCTAGGTCGGAACAAGTTGATAGGACCCCCTTTTTTACGT   1911
Gen1       CAAGCTCATGAGCTTATTATCCTAGGTCGGAACAAGTTGATAGGACCCCCTTTTTTACGT   1911
eGen1      CAAGCTCATGAGCTTATTATCCTAGGTCGGAACAAGTTGATAGGACCCCCTTTTTTACGT   1920
eGen2      CAAGCTCATGAGCTTATTATCCTAGGTCGGAACAAGTTGATAGGATCCCCTTTTTTACGT   1920
           ********************************************* **************
              6     7
Potato     CCCTATGTTC-CCCCGTGTGGCGACATGGGGGCGAAAAAAGGAAAGAGAGGGATGGGGTT   1979
Tobacco    CCCCATGTTCCCCCCGTGTGGCGACATGGGGGCGAAAAAAGGAAAGAGAGGGATGGGGTT   1971
Gen1       CCCCATGTTCCCCCCGTGTGGCGACATGGGGGCGAAAAAAGGAAAGAGAGGGATGGGGTT   1971
eGen1      CCCCATGTTCCCCCCGTGTGGCGACATGGGGGCGAAAAAAGGAAAGAGAGGGATGGGGTT   1980
eGen2      CCCTATGTTC-CCCCGTGTGGCGACATGGGGGCGAAAAAAGGAAAGAGAGGGATGGGGTT   1979
           *** ****** *************************************************

Potato     TCTCTCGCTTTTGGCATAGCGGGCCCCCAGTGGGAGGCTCGCACGACGGGCTATTAGCTC   2039
Tobacco    TCTCTCGCTTTTGGCATAGCGGGCCCCCAGTGGGAGGCTCGCACGACGGGCTATTAGCTC   2031
Gen1       TCTCTCGCTTTTGGCATAGCGGGCCCCCAGTGGGAGGCTCGCACGACGGGCTATTAGCTC   2031
eGen1      TCTCTCGCTTTTGGCATAGCGGGCCCCCAGTGGGAGGCTCGCACGACGGGCTATTAGCTC   2040
eGen2      TCTCTCGCTTTTGGCATAGCGGGCCCCCAGTGGGAGGCTCGCACGACGGGCTATTAGCTC   2039
           ************************************************************

Potato     AGTGGTAGAGCGCGCCCCTGATAATTGCGTCGTTGTGCCTGGGCTGTGAGGGCTCTCAGC   2099
Tobacco    AGTGGTAGAGCGCGCCCCTGATAATTGCGTCGTTGTGCCTGGGCTGTGAGGGCTCTCAGC   2091
Gen1       AGTGGTAGAGCGCGCCCCTGATAATTGCGTCGTTGTGCCTGGGCTGTGAGGGCTCTCAGC   2091
```

```
eGen1         AGTGGTAGAGCGCGCCCCTGATAATTGCGTCGTTGTGCCTGGGCTGTGAGGGCTCTCAGC   2100
eGen2         AGTGGTAGAGCGCGCCCCTGATAATTGCGTCGTTGTGCCTGGGCTGTGAGGGCTCTCAGC   2099
              ************************************************************

Potato        CACATGGATAGTTCAATGTGCTCATCGGCGCCTGACCCTGAGATGTGGATCATCCAAGGC   2159
Tobacco       CACATGGATAGTTCAATGTGCTCATCGGCGCCTGACCCTGAGATGTGGATCATCCAAGGC   2151
Gen1          CACATGGATAGTTCAATGTGCTCATCGGCGCCTGACCCTGAGATGTGGATCATCCAAGGC   2151
eGen1         CACATGGATAGTTCAATGTGCTCATCGGCGCCTGACCCTGAGATGTGGATCATCCAAGGC   2160
eGen2         CACATGGATAGTTCAATGTGCTCATCGGCGCCTGACCCTGAGATGTGGATCATCCAAGGC   2159
              ************************************************************

Potato        ACATTAGCATGGCGTACTCCTCCTGTTCGAACCGGGGTTTGAAACCAAACTCCTCCTCAG   2219
Tobacco       ACATTAGCATGGCGTACTCCTCCTGTTCGAACCGGGGTTTGAAACCAAACTCCTCCTCAG   2211
Gen1          ACATTAGCATGGCGTACTCCTCCTGTTCGAACCGGGGTTTGAAACCAAACTCCTCCTCAG   2211
eGen1         ACATTAGCATGGCGTACTCCTCCTGTTCGAACCGGGGTTTGAAACCAAACTCCTCCTCAG   2220
eGen2         ACATTAGCATGGCGTACTCCTCCTGTTCGAACCGGGGTTTGAAACCAAACTCCTCCTCAG   2219
              ************************************************************
                                          oriA2
Potato        GAGGATAGATGGGGCGATTCGGGTGAGATCCAATGTAGATCCAACTTTCGATTCACTCGT   2279
Tobacco       GAGGATAGATGGGGCGATTCGGGTGAGATCCAATGTAGATCCAACTTTCGATTCACTCGT   2271
Gen1          GAGGATAGATGGGGCGATTCGGGTGAGATCCAATGTAGATCCAACTTTCGATTCACTCGT   2271
eGen1         GAGGATAGATGGGGCGATTCGGGTGAGATCCAATGTAGATCCAACTTTCGATTCACTCGT   2280
eGen2         GAGGATAGATGGGGCGATTCGGGTGAGATCCAATGTAGATCCAACTTTCGATTCACTCGT   2279
              ************************************************************

Potato        GGGATCCGGGCGGTCCGGGGGGGACCACCACGGCTCCTCTCTTCTCGAGAATCCATACAT   2339
Tobacco       GGGATCCGGGCGGTCCGGGGGGGACCACCACGGCTCCTCTCTTCTCGAGAATCCATACAT   2331
Gen1          GGGATCCGGGCGGTCCGGGGGGGACCACCACGGCTCCTCTCTTCTCGAGAATCCATACAT   2331
eGen1         GGGATCCGGGCGGTCCGGGGGGGACCACCACGGCTCCTCTCTTCTCGAGAATCCATACAT   2340
eGen2         GGGATCCGGGCGGTCCGGGGGGGACCACCACGGCTCCTCTCTTCTCGAGAATCCATACAT   2339
              ************************************************************
                                                          8
Potato        CCCTTATCAGTGTATGGACAGCTATCTCTCGAGCACAGGTTTAGGTTCGGCCTCAATGGG   2399
Tobacco       CCCTTATCAGTGTATGGACAGCTATCTCTCGAGCACAGGTTTAG---------CAATGGG   2382
Gen1          CCCTTATCAGTGTATGGACAGCTATCTCTCGAGCACAGGTTTAG---------CAATGGG   2382
eGen1         CCCTTATCAGTGTATGGACAGCTATCTCTCGAGCACAGGTTTAG---------CAATGGG   2391
eGen2         CCCTTATCAGTGTATGGACAGCTATCTCTCGAGCACAGGTTTAGGTTCGGCCTCAATGGG   2399
              ********************************************         *******
                                                                9
Potato        AAAATAAAATGGAGCACCTAACAACGCATCTTCACAGACCAAGAACTACGAGATCACCCC   2459
Tobacco       AAAATAAAATGGAGCACCTAACAACGCATCTTCACAGACCAAGAACTACGAGATCGCCCC   2442
Gen1          AAAATAAAATGGAGCACCTAACAACGCATCTTCACAGACCAAGAACTACGAGATCGCCCC   2442
eGen1         AAAATAAAATGGAGCACCTAACAACGCATCTTCACAGACCAAGAACTACGAGATCGCCCC   2451
eGen2         AAAATAAAATGGAGCACCTAACAACGCATCTTCACAGACCAAGAACTACGAGATCACCCC   2459
              *******************************************************.****
                                                                   10
Potato        TTTCATTCTGGGGTGACGGAGGGATCGTACCATTCGAGCCGTTTTTTTCTTGACTCGAAA   2519
Tobacco       TTTCATTCTGGGGTGACGGAGGGATCGTACCATTCGAGCCGTTTTTTTCTTGACTCGA--   2500
Gen1          TTTCATTCTGGGGTGACGGAGGGATCGTACCATTCGAGCCGTTTTTTTCTTGACTCGA--   2500
eGen1         TTTCATTCTGGGGTGACGGAGGGATCGTACCATTCGAGCCGTTTTTTTCTTGACTCGAAA   2511
eGen2         TTTCATTCTGGGGTGACGGAGGGATCGTACCATTCGAGCCGTTTTTTTCTTGACTCGAAA   2519
              **********************************************************
                                                                   11
Potato        TCGAAATGGGAGCAGGTTTGAAAAAGGATCTTAGAGTGTCTAGGGTTGGGCCAGGAGGGT   2579
Tobacco       ----AATGGGAGCAGGTTTGAAAAAGGATCTTAGAGTGTCTAGGGTTGGGCCAGGAGGGT   2556
Gen1          ----AATGGGAGCAGGTTTGAAAAAGGATCTTAGAGTGTCTAGGGTTGGGCCAGGAGGCT   2556
eGen1         TCGAAATGGGAGCAGGTTTGAAAAAGGATCTTAGAGTGTCTAGGGTTGGGCCAGGAGGCT   2571
eGen2         TCGAAATGGGAGCAGGTTTGAAAAAGGATCTTAGAGTGTCTAGGGTTGGGCCAGGAGGGT   2579
                  ****************************************************** *

Potato        CTCTTAACGCCTTCTTTTTTCTTCTCATCGGAGTTATTTCACAAAGACTTGCCAGGGTAA   2639
Tobacco       CTCTTAACGCCTTCTTTTTTCTTCTCATCGGAGTTATTTCACAAAGACTTGCCAGGGTAA   2616
Gen1          CTCTTAACGCCTTCTTTTTTCTTCTCATCGGAGTTATTTCACAAAGACTTGCCAGGGTAA   2616
```

```
eGen1          CTCTTAACGCCTTCTTTTTTCTTCTCATCGGAGTTATTTCACAAAGACTTGCCAGGGTAA    2631
eGen2          CTCTTAACGCCTTCTTTTTTCTTCTCATCGGAGTTATTTCACAAAGACTTGCCAGGGTAA    2639
               ************************************************************

Potato         GGAAGAAGGGGGGAACAAGCACACTTGGAGAGCGCAGTACAACGGAGAGTTGTATGCTGC    2699
Tobacco        GGAAGAAGGGGGGAACAAGCACACTTGGAGAGCGCAGTACAACGGAGAGTTGTATGCTGC    2676
Gen1           GGAAGAAGGGGGGAACAAGCACACTTGGAGAGCGCAGTACAACGGAGAGTTGTATGCTGC    2676
eGen1          GGAAGAAGGGGGGAACAAGCACACTTGGAGAGCGCAGTACAACGGAGAGTTGTATGCTGC    2691
eGen2          GGAAGAAGGGGGGAACAAGCACACTTGGAGAGCGCAGTACAACGGAGAGTTGTATGCTGC    2699
               ************************************************************

Potato         GTTCGGGAAGGATGAATCGCTCCCGAAAAGGAATCTATTGATTCTCTCCCAATTGGTTGG    2759
Tobacco        GTTCGGGAAGGATGAATCGCTCCCGAAAAGGAATCTATTGATTCTCTCCCAATTGGTTGG    2736
Gen1           GTTCGGGAAGGATGAATCGCTCCCGAAAAGGAATCTATTGATTCTCTCCCAATTGGTTGG    2736
eGen1          GTTCGGGAAGGATGAATCGCTCCCGAAAAGGAATCTATTGATTCTCTCCCAATTGGTTGG    2751
eGen2          GTTCGGGAAGGATGAATCGCTCCCGAAAAGGAATCTATTGATTCTCTCCCAATTGGTTGG    2759
               ************************************************************
                                          oriA1  12
Potato         ACCGTAGGTGCGATGATTTACTTCACGGGCGAGGTCTCTGGTTCAAGTCCAGGATGGCCC    2819
Tobacco        ACCGTAGGTGCGATGATTTACTTCACGGGCGAGGTCTCTGGTTCAAGTCCAGGATGGCCC    2796
Gen1           ACCGTAGGTGCGATGATTTACTTCACGGGCGAGCTCTCTGGTTCAAGTCCAGGATGGCCC    2796
eGen1          ACCGTAGGTGCGATGATTTACTTCACGGGCGAGGTCTCTGGTTCAAGTCCAGGATGGCCC    2811
eGen2          ACCGTAGGTGCGATGATTTACTTCACGGGCGAGGTCTCTGGTTCAAGTCCAGGATGGCCC    2819
               ********************************* **************************
                                                    I  13
Potato         AGCTGCGCCAGGGAAAAGAATAGAAGAAGCATCTGACT----------------------    2857
Tobacco        AGCTGCGCCAGGGAAAAGAATAGAAGAAGCATCTGACT----------------------    2834
Gen1           AGCTGCGCCAGGGAAAAGAATAGAAGAAGCATCTGGTTTAAACACTAGTCGACTCTAGAT    2856
eGen1          AGCTGCGCCAGGGAAAAGAATAGAAGAAGCATCTGACT----------------------    2849
eGen2          AGCTGCGCCAGGGAAAAGAATAGAAGAAGCATCTGACT----------------------    2857
               ***********************************. *

Potato         --------------ACTTCATGCATGCTCCACTTGGCTCGGGGGGATATAGCTCAGTTGG    2903
Tobacco        --------------ACTTCATGCATGCTCCACTTGGCTCGGGGGGATATAGCTCAGTTGG    2880
Gen1           TAATTAACGCGCGCGCTTCATGCATGCTCCACTTGGCTCGGGGGGATATAGCTCAGTTGG    2916
eGen1          --------------ACTTCATGCATGCTCCACTTGGCTCGGGGGGATATAGCTCAGTTGG    2895
eGen2          --------------ACTTCATGCATGCTCCACTTGGCTCGGGGGGATATAGCTCAGTTGG    2903
                             .*********************************************

Potato         TAGAGCTCCGCTCTTGCAATTGGGTCGTTGCGATTACGGGTTGGATGTCTAATTGTCCAG    2963
Tobacco        TAGAGCTCCGCTCTTGCAATTGGGTCGTTGCGATTACGGGTTGGATGTCTAATTGTCCAG    2940
Gen1           TAGAGCTCCGCTCTTGCAATTGGGTCGTTGCGATTACGGGTTGGATGTCTAATTGTCCAG    2976
eGen1          TAGAGCTCCGCTCTTGCAATTGGGTCGTTGCGATTACGGGTTGGATGTCTAATTGTCCAG    2955
eGen2          TAGAGCTCCGCTCTTGCAATTGGGTCGTTGCGATTACGGGTTGGATGTCTAATTGTCCAG    2963
               ************************************************************
                NICE
Potato         GCGGTAATGATAGTATCTTGTACCTGAACCGGTGGCTCACTTTTTCTAAGTAATGGGGAA    3023
Tobacco        GCGGTAATGATAGTATCTTGTACCTGAACCGGTGGCTCACTTTTTCTAAGTAATGGGGAA    3000
Gen1           GCGGTAATGATAGTATCTTGTACCTGAACCGGTGGCTCACTTTTTCTAAGTAATGGGGAA    3036
eGen1          GCGGTAATGATAGTATCTTGTACCTGAACCGGTGGCTCACTTTTTCTAAGTAATGGGGAA    3015
eGen2          GCGGTAATGATAGTATCTTGTACCTGAACCGGTGGCTCACTTTTTCTAAGTAATGGGGAA    3023
               ************************************************************

Potato         GAGGACCGAAACGTGCCACTGAAAGACTCTACTGAGACAAAGATGGGCTGTCAAGAACGT    3083
Tobacco        GAGGACCGAAACGTGCCACTGAAAGACTCTACTGAGACAAAGATGGGCTGTCAAGAACGT    3060
Gen1           GAGGACCGAAACGTGCCACTGAAAGACTCTACTGAGACAAAGATGGGCTGTCAAGAACGT    3096
eGen1          GAGGACCGAAACGTGCCACTGAAAGACTCTACTGAGACAAAGATGGGCTGTCAAGAACGT    3075
eGen2          GAGGACCGAAACGTGCCACTGAAAGACTCTACTGAGACAAAGATGGGCTGTCAAGAACGT    3083
               ************************************************************

Potato         AGAGGAGGTAGGATGGGCAGTTGGTCAGATCTAGTATGGATCGTACATGGACGGTAGTTG    3143
Tobacco        AGAGGAGGTAGGATGGGCAGTTGGTCAGATCTAGTATGGATCGTACATGGACGGTAGTTG    3120
Gen1           AGAGGAGGTAGGATGGGCAGTTGGTCAGATCTAGTATGGATCGTACATGGACGGTAGTTG    3156
```

```
eGen1           AGAGGAGGTAGGATGGGCAGTTGGTCAGATCTAGTATGGATCGTACATGGACGGTAGTTG   3135
eGen2           AGAGGAGGTAGGATGGGCAGTTGGTCAGATCTAGTATGGATCGTACATGGACGGTAGTTG   3143
                ************************************************************

Potato          GAGTCGGCGGCTCTCCCAGGGTTCCCTCATCTGAGATCTCTGGGGAAGAGGATCAAGTTG   3203
Tobacco         GAGTCGGCGGCTCTCCCAGGGTTCCCTCATCTGAGATCTCTGGGGAAGAGGATCAAGTTG   3180
Gen1            GAGTCGGCGGCTCTCCCAGGGTTCCCTCATCTGAGATCTCTGGGGAAGAGGATCAAGTTG   3216
eGen1           GAGTCGGCGGCTCTCCCAGGGTTCCCTCATCTGAGATCTCTGGGGAAGAGGATCAAGTTG   3195
eGen2           GAGTCGGCGGCTCTCCCAGGGTTCCCTCATCTGAGATCTCTGGGGAAGAGGATCAAGTTG   3203
                ************************************************************

Potato          GCCCTTGCGAACAGCTTGATGCACTATCTCCCTTCAACCCTTTGAGCGAAATGCGGCAAA   3263
Tobacco         GCCCTTGCGAACAGCTTGATGCACTATCTCCCTTCAACCCTTTGAGCGAAATGCGGCAAA   3240
Gen1            GCCCTTGCGAACAGCTTGATGCACTATCTCCCTTCAACCCTTTGAGCGAAATGCGGCAAA   3276
eGen1           GCCCTTGCGAACAGCTTGATGCACTATCTCCCTTCAACCCTTTGAGCGAAATGCGGCAAA   3255
eGen2           GCCCTTGCGAACAGCTTGATGCACTATCTCCCTTCAACCCTTTGAGCGAAATGCGGCAAA   3263
                ************************************************************

Potato          AGAAAAGGAAGGAAAATCCATGGACCGACCCCATCATCTCCACCCCGTAGGAACTACGAG   3323
Tobacco         AGAAAAGGAAGGAAAATCCATGGACCGACCCCATCATCTCCACCCCGTAGGAACTACGAG   3300
Gen1            AGAAAAGGAAGGAAAATCCATGGACCGACCCCATCATCTCCACCCCGTAGGAACTACGAG   3336
eGen1           AGAAAAGGAAGGAAAATCCATGGACCGACCCCATCATCTCCACCCCGTAGGAACTACGAG   3315
eGen2           AGAAAAGGAAGGAAAATCCATGGACCGACCCCATCATCTCCACCCCGTAGGAACTACGAG   3323
                ************************************************************

Potato          ATCACCCCAAGGACGCCTTCGGCATCCAGGGGTCACGGACCGACCATAGAACCCTGTTCA   3383
Tobacco         ATCACCCCAAGGACGCCTTCGGCATCCAGGGGTCACGGACCGACCATAGAACCCTGTTCA   3360
Gen1            ATCACCCCAAGGACGCCTTCGGCATCCAGGGGTCACGGACCGACCATAGAACCCTGTTCA   3396
eGen1           ATCACCCCAAGGACGCCTTCGGCATCCAGGGGTCACGGACCGACCATAGAACCCTGTTCA   3375
eGen2           ATCACCCCAAGGACGCCTTCGGCATCCAGGGGTCACGGACCGACCATAGAACCCTGTTCA   3383
                ************************************************************

Potato          ATAAGTGGAACGCATTAGCTGTCCGCTCTCAGGTTGGGCAGTCAGGGTCGGAGAAGGGCA   3443
Tobacco         ATAAGTGGAACGCATTAGCTGTCCGCTCTCAGGTTGGGCAGTCAGGGTCGGAGAAGGGCA   3420
Gen1            ATAAGTGGAACGCATTAGCTGTCCGCTCTCAGGTTGGGCAGTCAGGGTCGGAGAAGGGCA   3456
eGen1           ATAAGTGGAACGCATTAGCTGTCCGCTCTCAGGTTGGGCAGTCAGGGTCGGAGAAGGGCA   3435
eGen2           ATAAGTGGAACGCATTAGCTGTCCGCTCTCAGGTTGGGCAGTCAGGGTCGGAGAAGGGCA   3443
                ************************************************************
                            14
Potato          ATGACTCATTCTTAAAACCAGCGTTCTTAAGACCAAAGAGTCGGGCGGAAGGGGGGGAAA   3503
Tobacco         ATGACTCATTCT------------------------------------------------   3432
Gen1            ATGACTCATTCT------------------------------------------------   3468
eGen1           ATGACTCATTCTTAAAACCAGCGTTCTTAAGACCAAAGAGTCGGGCGGAAGGGGGGGAAA   3495
eGen2           ATGACTCATTCTTAAAACCAGCGTTCTTAAGACCAAAGAGTCGGGCGGAAGGGGGGGAAA   3503
                ************

Potato          GCCCTCCGTTCCTGGTTCTCCTGTAGTTGGATCCTCCGGAACCACAAGAATCCTTAGTTA   3563
Tobacco         ------------------------------------------------------TAGTTA   3438
Gen1            ------------------------------------------------------TAGTTA   3474
eGen1           GCCCTCCGTTCCTGGTTCTCCTGTAGTTGGATCCTCCGGAACCACAAGAATCCTTAGTTA   3555
eGen2           GCCCTCCGTTCCTGGTTCTCCTGTAGTTGGATCCTCCGGAACCACAAGAATCCTTAGTTA   3563
                                                                      ******

Potato          GAATGGGATTCCAACTCAGCACCTTTTGAGTGAGATTTTGAGAAGAGTTGCTCTTTGGAG   3623
Tobacco         GAATGGGATTCCAACTCAGCACCTTTTGAGTGAGATTTTGAGAAGAGTTGCTCTTTGGAG   3498
Gen1            GAATGGGATTCCAACTCAGCACCTTTTGAGTGAGATTTTGAGAAGAGTTGCTCTTTGGAG   3534
eGen1           GAATGGGATTCCAACTCAGCACCTTTTGAGTGAGATTTTGAGAAGAGTTGCTCTTTGGAG   3615
eGen2           GAATGGGATTCCAACTCAGCACCTTTTGAGTGAGATTTTGAGAAGAGTTGCTCTTTGGAG   3623
                ************************************************************

Potato          AGCACAGTACGATGAAAGTTGTAAGCTGTGTTCGGGGGGGAGTTATTGTCTATCGTTGGC   3683
Tobacco         AGCACAGTACGATGAAAGTTGTAAGCTGTGTTCGGGGGGGAGTTATTGTCTATCGTTGGC   3558
Gen1            AGCACAGTACGATGAAAGTTGTAAGCTGTGTTCGGGGGGGAGTTATTGTCTATCGTTGGC   3594
```

```
eGen1      AGCACAGTACGATGAAAGTTGTAAGCTGTGTTCGGGGGGGAGTTATTGTCTATCGTTGGC   3675
eGen2      AGCACAGTACGATGAAAGTTGTAAGCTGTGTTCGGGGGGGAGTTATTGTCTATCGTTGGC   3683
           ************************************************************

Potato     CTCTATGGTAGAATCAGTCGGGGGACCTGAGAGGCGGTGGTTTACCCTGCGGCGGATGTC   3743
Tobacco    CTCTATGGTAGAATCAGTCGGGGGACCTGAGAGGCGGTGGTTTACCCTGCGGCGGATGTC   3618
Gen1       CTCTATGGTAGAATCAGTCGGGGGACCTGAGAGGCGGTGGTTTACCCTGCGGCGGATGTC   3654
eGen1      CTCTATGGTAGAATCAGTCGGGGGACCTGAGAGGCGGTGGTTTACCCTGCGGCGGATGTC   3735
eGen2      CTCTATGGTAGAATCAGTCGGGGGACCTGAGAGGCGGTGGTTTACCCTGCGGCGGATGTC   3743
           ************************************************************

Potato     AGCGGTTCGAGTCCGCTTATCTCCAACTCGTGAACTTAGCCGATACAAAGCTTTATGATA   3803
Tobacco    AGCGGTTCGAGTCCGCTTATCTCCAACTCGTGAACTTAGCCGATACAAAGCTTTATGATA   3678
Gen1       AGCGGTTCGAGTCCGCTTATCTCCAACTCGTGAACTTAGCCGATACAAAGCTTTATGATA   3714
eGen1      AGCGGTTCGAGTCCGCTTATCTCCAACTCGTGAACTTAGCCGATACAAAGCTTTATGATA   3795
eGen2      AGCGGTTCGAGTCCGCTTATCTCCAACTCGTGAACTTAGCCGATACAAAGCTTTATGATA   3803
           ************************************************************

Potato     GCACCCAATTTTTCCGATTCGGCGGTTCGATCTATGATTTATCATTCATGGACGTTGATA   3863
Tobacco    GCACCCAATTTTTCCGATTCGGCGGTTCGATCTATGATTTATCATTCATGGACGTTGATA   3738
Gen1       GCACCCAATTTTTCCGATTCGGCGGTTCGATCTATGATTTATCATTCATGGACGTTGATA   3774
eGen1      GCACCCAATTTTTCCGATTCGGCGGTTCGATCTATGATTTATCATTCATGGACGTTGATA   3855
eGen2      GCACCCAATTTTTCCGATTCGGCGGTTCGATCTATGATTTATCATTCATGGACGTTGATA   3863
           ************************************************************

Potato     AGATCCATCCATTTAGCAGCACCTTAGGATGGCATAGCCTTAAAAGTGAAGGGCGAGGTT   3923
Tobacco    AGATCCATCCATTTAGCAGCACCTTAGGATGGCATAGCCTTAAAAGTGAAGGGCGAGGTT   3798
Gen1       AGATCCATCCATTTAGCAGCACCTTAGGATGGCATAGCCTTAAAAGTGAAGGGCGAGGTT   3834
eGen1      AGATCCATCCATTTAGCAGCACCTTAGGATGGCATAGCCTTAAAAGTGAAGGGCGAGGTT   3915
eGen2      AGATCCATCCATTTAGCAGCACCTTAGGATGGCATAGCCTTAAAAGTGAAGGGCGAGGTT   3923
           ************************************************************
                                                       15
Potato     CAAACGAGGAAAGGCTTACGGTGGATACCTAGGCACCCAGAGACGAGGAAGGGCGTAGTA   3983
Tobacco    CAAACGAGGAAAGGCTTACGGTGGATACCTAGGCACCCAGAGACGAGGAAGGGCGTAGTA   3858
Gen1       CAAACGAGGAAAGGCTTACGGTGGATACCTAGGCACCCAGAGACTAGGAAGGGCGTAGTA   3894
eGen1      CAAACGAGGAAAGGCTTACGGTGGATACCTAGGCACCCAGAGACTAGGAAGGGCGTAGTA   3975
eGen2      CAAACGAGGAAAGGCTTACGGTGGATACCTAGGCACCCAGAGACGAGGAAGGGCGTAGTA   3983
           ******************************************** ***************

Potato     ATCGACGAAATGCTTCGGGGAGTTGAAAATAAGCATAGATCCGGAGATTCCCGAATAGGG   4043
Tobacco    ATCGACGAAATGCTTCGGGGAGTTGAAAATAAGCATAGATCCGGAGATTCCCGAATAGGG   3918
Gen1       ATCGACGAAATGCTTCGGGGAGTTGAAAATAAGCATAGATCCGGAGATTCCCGAATAGGG   3954
eGen1      ATCGACGAAATGCTTCGGGGAGTTGAAAATAAGCATAGATCCGGAGATTCCCGAATAGGG   4035
eGen2      ATCGACGAAATGCTTCGGGGAGTTGAAAATAAGCATAGATCCGGAGATTCCCGAATAGGG   4043
           ************************************************************

Potato     CAACCTTTCGAACTGCTGCTGAATCCATGGGCAGGCAAGAGACAACCTGGCGAACTGAAA   4103
Tobacco    CAACCTTTCGAACTGCTGCTGAATCCATGGGCAGGCAAGAGACAACCTGGCGAACTGAAA   3978
Gen1       CAACCTTTCGAACTGCTGCTGAATCCATGGGCAGGCAAGAGACAACCTGGCGAACTGAAA   4014
eGen1      CAACCTTTCGAACTGCTGCTGAATCCATGGGCAGGCAAGAGACAACCTGGCGAACTGAAA   4095
eGen2      CAACCTTTCGAACTGCTGCTGAATCCATGGGCAGGCAAGAGACAACCTGGCGAACTGAAA   4103
           ************************************************************

Potato     CATCTTAGTAGCCAGAGGAAAAGAAAGCAAAAGCGATTCCCGTAGTAGCGGCGAGCGAAA   4163
Tobacco    CATCTTAGTAGCCAGAGGAAAAGAAAGCAAAAGCGATTCCCGTAGTAGCGGCGAGCGAAA   4038
Gen1       CATCTTAGTAGCCAGAGGAAAAGAAAGCAAAAGCGATTCCCGTAGTAGCGGCGAGCGAAA   4074
eGen1      CATCTTAGTAGCCAGAGGAAAAGAAAGCAAAAGCGATTCCCGTAGTAGCGGCGAGCGAAA   4155
eGen2      CATCTTAGTAGCCAGAGGAAAAGAAAGCAAAAGCGATTCCCGTAGTAGCGGCGAGCGAAA   4163
           ************************************************************

Potato     TGGGAGCAGCCTAAACCGTGAAAACGGGGTTGTGGGAGAGCAATACAAGCGTCGTGCTGC   4223
Tobacco    TGGGAGCAGCCTAAACCGTGAAAACGGGGTTGTGGGAGAGCAATACAAGCGTCGTGCTGC   4098
Gen1       TGGGAGCAGCCTAAACCGTGAAAACGGGGTTGTGGGAGAGCAATACAAGCGTCGTGCTGC   4134
```

```
eGen1      TGGGAGCAGCCTAAACCGTGAAAACGGGGTTGTGGGAGAGCAATACAAGCGTCGTGCTGC  4215
eGen2      TGGGAGCAGCCTAAACCGTGAAAACGGGGTTGTGGGAGAGCAATACAAGCGTCGTGCTGC  4223
           ************************************************************

                         16
Potato     TAGGCGAAGCAGCCTGAATGCTGCACCCTAGATGGCGAAAGTCCAGTAGCCGAAAGCATC  4283
Tobacco    TAGGCGAAGCAGCCCGAATGCTGCACCCTAGATGGCGAAAGTCCAGTAGCCGAAAGCATC  4158
Gen1       TAGGCGAAGCAGCCCGAATGCTGCACCCTAGATGGCGAAAGTCCAGTAGCCGAAAGCATC  4194
eGen1      TAGGCGAAGCAGCCCGAATGCTGCACCCTAGATGGCGAAAGTCCAGTAGCCGAAAGCATC  4275
eGen2      TAGGCGAAGCAGCCTGAATGCTGCACCCTAGATGGCGAAAGTCCAGTAGCCGAAAGCATC  4283
           ************** *********************************************

                    17
Potato     ACTAGCTTACGCTCTGACCCGAGTAGCATGGGGCACGTGGAATCCCGTGTGAATCAGCAA  4343
Tobacco    ACTAGCTTATGCTCTGACCCGAGTAGCATGGGGCACGTGGAATCCCGTGTGAATCAGCAA  4218
Gen1       ACTAGCTTATGCTCTGACCCGAGTAGCATGGGGCACGTGGAATCCCGTGTGAATCAGCAA  4254
eGen1      ACTAGCTTATGCTCTGACCCGAGTAGCATGGGGCACGTGGAATCCCGTGTGAATCAGCAA  4335
eGen2      ACTAGCTTACGCTCTGACCCGAGTAGCATGGGGCACGTGGAATCCCGTGTGAATCAGCAA  4343
           ********* **************************************************

Potato     GGACCACCTTGCAAGGCTAAATACTCCTGGGTGACCGATAGCGAAGTAGTACCGTGAGGG  4403
Tobacco    GGACCACCTTGCAAGGCTAAATACTCCTGGGTGACCGATAGCGAAGTAGTACCGTGAGGG  4278
Gen1       GGACCACCTTGCAAGGCTAAATACTCCTGGGTGACCGATAGCGAAGTAGTACCGTGAGGG  4314
eGen1      GGACCACCTTGCAAGGCTAAATACTCCTGGGTGACCGATAGCGAAGTAGTACCGTGAGGG  4395
eGen2      GGACCACCTTGCAAGGCTAAATACTCCTGGGTGACCGATAGCGAAGTAGTACCGTGAGGG  4403
           ************************************************************

Potato     AAGGGTGAAAAGAACCCCCATCGGGGAGTGAAATAGAACATGAAACCGTAAGCTCCCAAG  4463
Tobacco    AAGGGTGAAAAGAACCCCCATCGGGGAGTGAAATAGAACATGAAACCGTAAGCTCCCAAG  4338
Gen1       AAGGGTGAAAAGAACCCCCATCGGGGAGTGAAATAGAACATGAAACCGTAAGCTCCCAAG  4374
eGen1      AAGGGTGAAAAGAACCCCCATCGGGGAGTGAAATAGAACATGAAACCGTAAGCTCCCAAG  4455
eGen2      AAGGGTGAAAAGAACCCCCATCGGGGAGTGAAATAGAACATGAAACCGTAAGCTCCCAAG  4463
           ************************************************************

Potato     CAGTGGGAGGAGCCAGGGCTCTGACCGCGTGCCTGTTGAAGAATGAGCCGGCGACTCATA  4523
Tobacco    CAGTGGGAGGAGCCAGGGCTCTGACCGCGTGCCTGTTGAAGAATGAGCCGGCGACTCATA  4398
Gen1       CAGTGGGAGGAGCCAGGGCTCTGACCGCGTGCCTGTTGAAGAATGAGCCGGCGACTCATA  4434
eGen1      CAGTGGGAGGAGCCAGGGCTCTGACCGCGTGCCTGTTGAAGAATGAGCCGGCGACTCATA  4515
eGen2      CAGTGGGAGGAGCCAGGGCTCTGACCGCGTGCCTGTTGAAGAATGAGCCGGCGACTCATA  4523
           ************************************************************

                                                              18
Potato     GGCAGTGGCTTGGTTAAGGGAACCCACCGGAGCCGTAGCGAAAGCGAGTCTTCATAGGGC  4583
Tobacco    GGCAGTGGCTTGGTTAAGGGAACCCACCGGAGCCGTAGCGAAAGCGAGTCTTCATAGGGC  4458
Gen1       GGCAGTGGCTTGGTTAAGGGAACCCACCGGAGCCGTAGCGAAAGCGAGTCATCATAGGGC  4494
eGen1      GGCAGTGGCTTGGTTAAGGGAACCCACCGGAGCCGTAGCGAAAGCGAGTCTTCATAGGGC  4575
eGen2      GGCAGTGGCTTGGTTAAGGGAACCCACCGGAGCCGTAGCGAAAGCGAGTCTTCATAGGGC  4583
           **************************************************:*********

Potato     AATTGTCACTGCTTATGGACCCGAACCTGGGTGATCTATCCATGACCAGGATGAAGCTTG  4643
Tobacco    AATTGTCACTGCTTATGGACCCGAACCTGGGTGATCTATCCATGACCAGGATGAAGCTTG  4518
Gen1       AATTGTCACTGCTTATGGACCCGAACCTGGGTGATCTATCCATGACCAGGATGAAGCTTG  4554
eGen1      AATTGTCACTGCTTATGGACCCGAACCTGGGTGATCTATCCATGACCAGGATGAAGCTTG  4635
eGen2      AATTGTCACTGCTTATGGACCCGAACCTGGGTGATCTATCCATGACCAGGATGAAGCTTG  4643
           ************************************************************

Potato     GGTGAAACTAAGTGGAGGTCCGAACCGACTGATGTTGAAGAATCAGCGGATGAGTTGTGG  4703
Tobacco    GGTGAAACTAAGTGGAGGTCCGAACCGACTGATGTTGAAGAATCAGCGGATGAGTTGTGG  4578
Gen1       GGTGAAACTAAGTGGAGGTCCGAACCGACTGATGTTGAAGAATCAGCGGATGAGTTGTGG  4614
eGen1      GGTGAAACTAAGTGGAGGTCCGAACCGACTGATGTTGAAGAATCAGCGGATGAGTTGTGG  4695
eGen2      GGTGAAACTAAGTGGAGGTCCGAACCGACTGATGTTGAAGAATCAGCGGATGAGTTGTGG  4703
           ************************************************************

Potato     TTAGGGGTGAAATGCCACTCGAACCCAGAGCTAGCTGGTTCTCCCCGAAATGCGTTGAGG  4763
Tobacco    TTAGGGGTGAAATGCCACTCGAACCCAGAGCTAGCTGGTTCTCCCCGAAATGCGTTGAGG  4638
Gen1       TTAGGGGTGAAATGCCACTCGAACCCAGAGCTAGCTGGTTCTCCCCGAAATGCGTTGAGG  4674
```

```
eGen1       TTAGGGGTGAAATGCCACTCGAACCCAGAGCTAGCTGGTTCTCCCCGAAATGCGTTGAGG   4755
eGen2       TTAGGGGTGAAATGCCACTCGAACCCAGAGCTAGCTGGTTCTCCCCGAAATGCGTTGAGG   4763
            ************************************************************

Potato      CGCAGCAGTTGACTGGACATCTAGGGGTAAAGCACTGTTTCGGTGCGGGCCGCGAGAGCG   4823
Tobacco     CGCAGCAGTTGACTGGACATCTAGGGGTAAAGCACTGTTTCGGTGCGGGCCGCGAGAGCG   4698
Gen1        CGCAGCAGTTGACTGGACATCTAGGGGTAAAGCACTGTTTCGGTGCGGGCCGCGAGAGCG   4734
eGen1       CGCAGCAGTTGACTGGACATCTAGGGGTAAAGCACTGTTTCGGTGCGGGCCGCGAGAGCG   4815
eGen2       CGCAGCAGTTGACTGGACATCTAGGGGTAAAGCACTGTTTCGGTGCGGGCCGCGAGAGCG   4823
            ************************************************************

Potato      GTACCAAATCGAGGCAAACTCTGAATACTAGATATGACCTCAAAATAACAGGGGTCAAGG   4883
Tobacco     GTACCAAATCGAGGCAAACTCTGAATACTAGATATGACCTCAAAATAACAGGGGTCAAGG   4758
Gen1        GTACCAAATCGAGGCAAACTCTGAATACTAGATATGACCTCAAAATAACAGGGGTCAAGG   4794
eGen1       GTACCAAATCGAGGCAAACTCTGAATACTAGATATGACCTCAAAATAACAGGGGTCAAGG   4875
eGen2       GTACCAAATCGAGGCAAACTCTGAATACTAGATATGACCTCAAAATAACAGGGGTCAAGG   4883
            ************************************************************
                       19
Potato      TCGGCTAGTGAGACGATGGGGGATAAGCTTCATCGTCGAGAGGGAAACAGCCCGGATCAC   4943
Tobacco     TCGGCTAGTGAGACGATGGGGGATAAGCTTCATCGTCGAGAGGGAAACAGCCCGGATCAC   4818
Gen1        TCGGCTAGTGACACGATGGGGGATAAGCTTCATCGTCGAGAGGGAAACAGCCCGGATCAC   4854
eGen1       TCGGCTAGTGAGACGATGGGGGATAAGCTTCATCGTCGAGAGGGAAACAGCCCGGATCAC   4935
eGen2       TCGGCTAGTGAGACGATGGGGGATAAGCTTCATCGTCGAGAGGGAAACAGCCCGGATCAC   4943
            *********** ************************************************
                                20
Potato      CAGCTAAGGCCCCTAAATGACCGCTCAGTGATAAAGGAGGTAGGGGTGCAGAGACAGCCA   5003
Tobacco     CAGCTAAGGCCCCTAAATGATCGCTCAGTGATAAAGGAGGTAGGGGTGCAGAGACAGCCA   4878
Gen1        CAGCTAAGGCCCCTAAATGATCGCTCAGTGATAAAGGAGGTAGGGGTGCAGAGACAGCCA   4914
eGen1       CAGCTAAGGCCCCTAAATGACCGCTCAGTGATAAAGGAGGTAGGGGTGCAGAGACAGCCA   4995
eGen2       CAGCTAAGGCCCCTAAATGACCGCTCAGTGATAAAGGAGGTAGGGGTGCAGAGACAGCCA   5003
            ******************** ***************************************

Potato      GGAGGTTTGCCTAGAAGCAGCCACCCTTGAAAGAGTGCGTAATAGCTCACTGATCGAGCG   5063
Tobacco     GGAGGTTTGCCTAGAAGCAGCCACCCTTGAAAGAGTGCGTAATAGCTCACTGATCGAGCG   4938
Gen1        GGAGGTTTGCCTAGAAGCAGCCACCCTTGAAAGAGTGCGTAATAGCTCACTGATCGAGCG   4974
eGen1       GGAGGTTTGCCTAGAAGCAGCCACCCTTGAAAGAGTGCGTAATAGCTCACTGATCGAGCG   5055
eGen2       GGAGGTTTGCCTAGAAGCAGCCACCCTTGAAAGAGTGCGTAATAGCTCACTGATCGAGCG   5063
            ************************************************************
                                            21
Potato      CTCTTGCGCCGAAGATGAACGGGGCTAAGCGGTCTGCCGAAGCTGTGGGATGTAAAAATA   5123
Tobacco     CTCTTGCGCCGAAGATGAACGGGGCTAAGCGATCTGCCGAAGCTGTGGGATGTAAAAATA   4998
Gen1        CTCTTGCGCCGAAGATGAACGGGGCTAAGCGATCTGCCGAAGCTGTGGGATGTAAAAATA   5034
eGen1       CTCTTGCGCCGAAGATGAACGGGGCTAAGCGGTCTGCCGAAGCTGTGGGATGTAAAAATA   5115
eGen2       CTCTTGCGCCGAAGATGAACGGGGCTAAGCGGTCTGCCGAAGCTGTGGGATGTAAAAATA   5123
            *******************************.****************************

Potato      CATCGGTAGGGGAGCGTTCCGCCTTAGAGAGAAGCCTCCGCGCGAGCGGTGGTGGACGAA   5183
Tobacco     CATCGGTAGGGGAGCGTTCCGCCTTAGAGAGAAGCCTCCGCGCGAGCGGTGGTGGACGAA   5058
Gen1        CATCGGTAGGGGAGCGTTCCGCCTTAGAGAGAAGCCTCCGCGCGAGCGGTGGTGGACGAA   5094
eGen1       CATCGGTAGGGGAGCGTTCCGCCTTAGAGAGAAGCCTCCGCGCGAGCGGTGGTGGACGAA   5175
eGen2       CATCGGTAGGGGAGCGTTCCGCCTTAGAGAGAAGCCTCCGCGCGAGCGGTGGTGGACGAA   5183
            ************************************************************

Potato      GCGGAAGCGAGAATGTCGGCTTGAGTAACGCAAACATTGGTGAGAATCCAATGCCCCGAA   5243
Tobacco     GCGGAAGCGAGAATGTCGGCTTGAGTAACGCAAACATTGGTGAGAATCCAATGCCCCGAA   5118
Gen1        GCGGAAGCGAGAATGTCGGCTTGAGTAACGCAAACATTGGTGAGAATCCAATGCCCCGAA   5154
eGen1       GCGGAAGCGAGAATGTCGGCTTGAGTAACGCAAACATTGGTGAGAATCCAATGCCCCGAA   5235
eGen2       GCGGAAGCGAGAATGTCGGCTTGAGTAACGCAAACATTGGTGAGAATCCAATGCCCCGAA   5243
            ************************************************************

Potato      AACCTAAGGGTTCCTCCGCAAGGTTCGTCCACGGAGGGTGAGTCAGGGCCTAAGATCAGG   5303
Tobacco     AACCTAAGGGTTCCTCCGCAAGGTTCGTCCACGGAGGGTGAGTCAGGGCCTAAGATCAGG   5178
Gen1        AACCTAAGGGTTCCTCCGCAAGGTTCGTCCACGGAGGGTGAGTCAGGGCCTAAGATCAGG   5214
```

```
eGen1          AACCTAAGGGTTCCTCCGCAAGGTTCGTCCACGGAGGGTGAGTCAGGGCCTAAGATCAGG   5295
eGen2          AACCTAAGGGTTCCTCCGCAAGGTTCGTCCACGGAGGGTGAGTCAGGGCCTAAGATCAGG   5303
               ************************************************************

                                                           22         23
Potato         CCGAAAGGCGTAGTCGATGGACAACAGGTGAATATTCCTGTACTACCCCTTGTTAGTCCC   5363
Tobacco        CCGAAAGGCGTAGTCGATGGACAACAGGTGAATATTCCTGTACTGCCCCTTGTTGGTCCC   5238
Gen1           CCGAAAGGCGTAGTCGATGGACAACAGGTGAATATTCCTGTACTGCCCCTTGTTGGTCCC   5274
eGen1          CCGAAAGGCGTAGTCGATGGACAACAGGTGAATATTCCTGTACTACCCCTTGTTAGTCCC   5355
eGen2          CCGAAAGGCGTAGTCGATGGACAACAGGTGAATATTCCTGTACTACCCCTTGTTAGTCCC   5363
               ********************************************.*********.*****

Potato         GAGGGACGGAGGAGGCTAGGTTAGCCGAAAGATGGTTATCGGTTCAAGAACGTAAGGTGT   5423
Tobacco        GAGGGACGGAGGAGGCTAGGTTAGCCGAAAGATGGTTATCGGTTCAAGAACGTAAGGTGT   5298
Gen1           GAGGGACGGAGGAGGCTAGGTTAGCCGAAAGATGGTTATCGGTTCAAGAACGTAAGGTGT   5334
eGen1          GAGGGACGGAGGAGGCTAGGTTAGCCGAAAGATGGTTATCGGTTCAAGAACGTAAGGTGT   5415
eGen2          GAGGGACGGAGGAGGCTAGGTTAGCCGAAAGATGGTTATCGGTTCAAGAACGTAAGGTGT   5423
               ************************************************************
                        24
Potato         CCCTGCTTTTTCAGGGTAAGAAGGGGTAGAGAAAATGCCTCGAGCCAATGTTCGAATACC   5483
Tobacco        CCCTGCTTTGTCAGGGTAAGAAGGGGTAGAGAAAATGCCTCGAGCCAATGTTCGAATACC   5358
Gen1           CCCTGCTTTGTCAGGGTAAGAAGGGGTAGAGAAAATGCCTCGAGCCAATGTTCGAATACC   5394
eGen1          CCCTGCTTTTTCAGGGTAAGAAGGGGTAGAGAAAATGCCTCGAGCCAATGTTCGAATACC   5475
eGen2          CCCTGCTTTTTCAGGGTAAGAAGGGGTAGAGAAAATGCCTCGAGCCAATGTTCGAATACC   5483
               ********* **************************************************
                                                                       25 26
Potato         AGGCGCTACGGCGCTGAAGTAACCCATGCCATACTCCCAGGAAAAGCTCGAACGACGTTA   5543
Tobacco        AGGCGCTACGGCGCTGAAGTAACCCATGCCATACTCCCAGGAAAAGCTCGAACGACTTTG   5418
Gen1           AGGCGCTACGGCGCTGAAGTAACCCATGCCATACTCCCAGGAAAAGCTCGAACGACTTTG   5454
eGen1          AGGCGCTACGGCGCTGAAGTAACCCATGCCATACTCCCAGGAAAAGCTCGAACGACGTTA   5535
eGen2          AGGCGCTACGGCGCTGAAGTAACCCATGCCATACTCCCAGGAAAAGCTCGAACGACGTTA   5543
               ******************************************************** **.

Potato         AGCAAGAGGGTACCTGTACCCGAAACCGACACAGGTGGGTAGGTAGAGAATACCTAGGGG   5603
Tobacco        AGCAAGAGGGTACCTGTACCCGAAACCGACACAGGTGGGTAGGTAGAGAATACCTAGGGG   5478
Gen1           AGCAAGAGGGTACCTGTACCCGAAACCGACACAGGTGGGTAGGTAGAGAATACCTAGGGG   5514
eGen1          AGCAAGAGGGTACCTGTACCCGAAACCGACACAGGTGGGTAGGTAGAGAATACCTAGGGG   5595
eGen2          AGCAAGAGGGTACCTGTACCCGAAACCGACACAGGTGGGTAGGTAGAGAATACCTAGGGG   5603
               ************************************************************

Potato         CGCGAGACAACTCTCTCTAAGGAACTCGGCAAAATAGCCCCGTAACTTCGGGAGAAGGGG   5663
Tobacco        CGCGAGACAACTCTCTCTAAGGAACTCGGCAAAATAGCCCCGTAACTTCGGGAGAAGGGG   5538
Gen1           CGCGAGACAACTCTCTCTAAGGAACTCGGCAAAATAGCCCCGTAACTTCGGGAGAAGGGG   5574
eGen1          CGCGAGACAACTCTCTCTAAGGAACTCGGCAAAATAGCCCCGTAACTTCGGGAGAAGGGG   5655
eGen2          CGCGAGACAACTCTCTCTAAGGAACTCGGCAAAATAGCCCCGTAACTTCGGGAGAAGGGG   5663
               ************************************************************

Potato         TGCCTCCTCACAAAGGGGGTCGCAGTGACCAGGCCCGGGCGACTGTTTACCAAAAACACA   5723
Tobacco        TGCCTCCTCACAAAGGGGGTCGCAGTGACCAGGCCCGGGCGACTGTTTACCAAAAACACA   5598
Gen1           TGCCTCCTCACAAAGGGGGTCGCAGTGACCAGGCCCGGGCGACTGTTTACCAAAAACACA   5634
eGen1          TGCCTCCTCACAAAGGGGGTCGCAGTGACCAGGCCCGGGCGACTGTTTACCAAAAACACA   5715
eGen2          TGCCTCCTCACAAAGGGGGTCGCAGTGACCAGGCCCGGGCGACTGTTTACCAAAAACACA   5723
               ************************************************************
                27
Potato         GGTCTCCGCAAAGTCGTAAGACCATGTATGGGGGCTGACGCCTGCCCAGTGCCGGAAGGT   5783
Tobacco        GGTCTCCGCAAAGTCGTAAGACCATGTATGGGGGCTGACGCCTGCCCAGTGCCGGAAGGT   5658
Gen1           GCTCTCCGCAAAGTCGTAAGACCATGTATGGGGGCTGACGCCTGCCCAGTGCCGGAAGGT   5694
eGen1          GGTCTCCGCAAAGTCGTAAGACCATGTATGGGGGCTGACGCCTGCCCAGTGCCGGAAGGT   5775
eGen2          GGTCTCCGCAAAGTCGTAAGACCATGTATGGGGGCTGACGCCTGCCCAGTGCCGGAAGGT   5783
               * **********************************************************

Potato         CAAGGAAGTTGGTGACCTGATGACAGGGGAGCCGGCGACCGAAGCCCCGGTGAACGGCGG   5843
Tobacco        CAAGGAAGTTGGTGACCTGATGACAGGGGAGCCGGCGACCGAAGCCCCGGTGAACGGCGG   5718
Gen1           CAAGGAAGTTGGTGACCTGATGACAGGGGAGCCGGCGACCGAAGCCCCGGTGAACGGCGG   5754
```

```
eGen1      CAAGGAAGTTGGTGACCTGATGACAGGGGAGCCGGCGACCGAAGCCCCGGTGAACGGCGG   5835
eGen2      CAAGGAAGTTGGTGACCTGATGACAGGGGAGCCGGCGACCGAAGCCCCGGTGAACGGCGG   5843
           ************************************************************

Potato     CCGTAACTATAACGGTCCTAAGGTAGCGAAATTCCTTGTCGGGTAAGTTCCGACCCGCAC   5903
Tobacco    CCGTAACTATAACGGTCCTAAGGTAGCGAAATTCCTTGTCGGGTAAGTTCCGACCCGCAC   5778
Gen1       CCGTAACTATAACGGTCCTAAGGTAGCGAAATTCCTTGTCGGGTAAGTTCCGACCCGCAC   5814
eGen1      CCGTAACTATAACGGTCCTAAGGTAGCGAAATTCCTTGTCGGGTAAGTTCCGACCCGCAC   5895
eGen2      CCGTAACTATAACGGTCCTAAGGTAGCGAAATTCCTTGTCGGGTAAGTTCCGACCCGCAC   5903
           ************************************************************

Potato     GAAAGGCGTAACGATCTGGGCACTGTCTCGGAGAGAGGCTCGGTGAAATAGACATGTCTG   5963
Tobacco    GAAAGGCGTAACGATCTGGGCACTGTCTCGGAGAGAGGCTCGGTGAAATAGACATGTCTG   5838
Gen1       GAAAGGCGTAACGATCTGGGCACTGTCTCGGAGAGAGGCTCGGTGAAATAGACATGTCTG   5874
eGen1      GAAAGGCGTAACGATCTGGGCACTGTCTCGGAGAGAGGCTCGGTGAAATAGACATGTCTG   5955
eGen2      GAAAGGCGTAACGATCTGGGCACTGTCTCGGAGAGAGGCTCGGTGAAATAGACATGTCTG   5963
           ************************************************************

Potato     TGAAGATGCGGACTACCTGCACCTGGACAGAAAGACCCTATGAAGCTTCACTGTTCCCTG   6023
Tobacco    TGAAGATGCGGACTACCTGCACCTGGACAGAAAGACCCTATGAAGCTTCACTGTTCCCTG   5898
Gen1       TGAAGATGCGGACTACCTGCACCTGGACAGAAAGACCCTATGAAGCTTCACTGTTCCCTG   5934
eGen1      TGAAGATGCGGACTACCTGCACCTGGACAGAAAGACCCTATGAAGCTTCACTGTTCCCTG   6015
eGen2      TGAAGATGCGGACTACCTGCACCTGGACAGAAAGACCCTATGAAGCTTCACTGTTCCCTG   6023
           ************************************************************

Potato     GGATTGGCTTTGGGCCTTTCCTGCGCAGCTTAGGTGGAAGGCGAAGAAGGCCTCCTTCCG   6083
Tobacco    GGATTGGCTTTGGGCCTTTCCTGCGCAGCTTAGGTGGAAGGCGAAGAAGGCCTCCTTCCG   5958
Gen1       GGATTGGCTTTGGGCCTTTCCTGCGCAGCTTAGGTGGAAGGCGAAGAAGGCCTCCTTCCG   5994
eGen1      GGATTGGCTTTGGGCCTTTCCTGCGCAGCTTAGGTGGAAGGCGAAGAAGGCCTCCTTCCG   6075
eGen2      GGATTGGCTTTGGGCCTTTCCTGCGCAGCTTAGGTGGAAGGCGAAGAAGGCCTCCTTCCG   6083
           ************************************************************

Potato     GGGGGGCCCGAGCCATCAGTGAGATACCACTCTGGAAGGGCTAGAATTCTAACCTTGTGT   6143
Tobacco    GGGGGGCCCGAGCCATCAGTGAGATACCACTCTGGAAGGGCTAGAATTCTAACCTTGTGT   6018
Gen1       GGGGGGCCCGAGCCATCAGTGAGATACCACTCTGGAAGGGCTAGAATTCTAACCTTGTGT   6054
eGen1      GGGGGGCCCGAGCCATCAGTGAGATACCACTCTGGAAGGGCTAGAATTCTAACCTTGTGT   6135
eGen2      GGGGGGCCCGAGCCATCAGTGAGATACCACTCTGGAAGGGCTAGAATTCTAACCTTGTGT   6143
           ************************************************************

Potato     CAGGACCTACGGGCCAAGGGACAGTCTCAGGTAGACAGTTTCTATGGGGCGTAGGCCTCC   6203
Tobacco    CAGGACCTACGGGCCAAGGGACAGTCTCAGGTAGACAGTTTCTATGGGGCGTAGGCCTCC   6078
Gen1       CAGGACCTACGGGCCAAGGGACAGTCTCAGGTAGACAGTTTCTATGGGGCGTAGGCCTCC   6114
eGen1      CAGGACCTACGGGCCAAGGGACAGTCTCAGGTAGACAGTTTCTATGGGGCGTAGGCCTCC   6195
eGen2      CAGGACCTACGGGCCAAGGGACAGTCTCAGGTAGACAGTTTCTATGGGGCGTAGGCCTCC   6203
           ************************************************************

Potato     CAAAAGGTAACGGAGGCGTGCAAAGGTTTCCTCGGGCCGGACGGAGATTGGCCCTCGAGT   6263
Tobacco    CAAAAGGTAACGGAGGCGTGCAAAGGTTTCCTCGGGCCGGACGGAGATTGGCCCTCGAGT   6138
Gen1       CAAAAGGTAACGGAGGCGTGCAAAGGTTTCCTCGGGCCGGACGGAGATTGGCCCTCGAGT   6174
eGen1      CAAAAGGTAACGGAGGCGTGCAAAGGTTTCCTCGGGCCGGACGGAGATTGGCCCTCGAGT   6255
eGen2      CAAAAGGTAACGGAGGCGTGCAAAGGTTTCCTCGGGCCGGACGGAGATTGGCCCTCGAGT   6263
           ************************************************************

Potato     GCAAAGGCAGAAGGGAGCTTGACTGCAAGACCCACCCGTCGAGCAGGGACGAAAGTCGGC   6323
Tobacco    GCAAAGGCAGAAGGGAGCTTGACTGCAAGACCCACCCGTCGAGCAGGGACGAAAGTCGGC   6198
Gen1       GCAAAGGCAGAAGGGAGCTTGACTGCAAGACCCACCCGTCGAGCAGGGACGAAAGTCGGC   6234
eGen1      GCAAAGGCAGAAGGGAGCTTGACTGCAAGACCCACCCGTCGAGCAGGGACGAAAGTCGGC   6315
eGen2      GCAAAGGCAGAAGGGAGCTTGACTGCAAGACCCACCCGTCGAGCAGGGACGAAAGTCGGC   6323
           ************************************************************

Potato     CTTAGTGATCCGACGGTGCCGAGTGGAAGGGCCGTCGCTCAACGGATAAAAGTTACTCTA   6383
Tobacco    CTTAGTGATCCGACGGTGCCGAGTGGAAGGGCCGTCGCTCAACGGATAAAAGTTACTCTA   6258
Gen1       CTTAGTGATCCGACGGTGCCGAGTGGAAGGGCCGTCGCTCAACGGATAAAAGTTACTCTA   6294
```

```
eGen1          CTTAGTGATCCGACGGTGCCGAGTGGAAGGGCCGTCGCTCAACGGATAAAAGTTACTCTA    6375
eGen2          CTTAGTGATCCGACGGTGCCGAGTGGAAGGGCCGTCGCTCAACGGATAAAAGTTACTCTA    6383
               ************************************************************

Potato         GGGATAACAGGCTGATCTTCCCCAAGAGCTCACATCGACGGGAAGGTTTGGCACCTCGAT    6443
Tobacco        GGGATAACAGGCTGATCTTCCCCAAGAGCTCACATCGACGGGAAGGTTTGGCACCTCGAT    6318
Gen1           GGGATAACAGGCTGATCTTCCCCAAGAGCTCACATCGACGGGAAGGTTTGGCACCTCGAT    6354
eGen1          GGGATAACAGGCTGATCTTCCCCAAGAGCTCACATCGACGGGAAGGTTTGGCACCTCGAT    6435
eGen2          GGGATAACAGGCTGATCTTCCCCAAGAGCTCACATCGACGGGAAGGTTTGGCACCTCGAT    6443
               ************************************************************

Potato         GTCGGCTCTTCGCCACCTGGGGCTGTAGTATGTTCCAAGGGTTGGGCTGTTCGCCCATTA    6503
Tobacco        GTCGGCTCTTCGCCACCTGGGGCTGTAGTATGTTCCAAGGGTTGGGCTGTTCGCCCATTA    6378
Gen1           GTCGGCTCTTCGCCACCTGGGGCTGTAGTATGTTCCAAGGGTTGGGCTGTTCGCCCATTA    6414
eGen1          GTCGGCTCTTCGCCACCTGGGGCTGTAGTATGTTCCAAGGGTTGGGCTGTTCGCCCATTA    6495
eGen2          GTCGGCTCTTCGCCACCTGGGGCTGTAGTATGTTCCAAGGGTTGGGCTGTTCGCCCATTA    6503
               ************************************************************

Potato         AAGCGGTACGTGAGCTGGGTTCAGAACGTCGTGAGACAGTTCGGTCCATATCCGGTGTGG    6563
Tobacco        AAGCGGTACGTGAGCTGGGTTCAGAACGTCGTGAGACAGTTCGGTCCATATCCGGTGTGG    6438
Gen1           AAGCGGTACGTGAGCTGGGTTCAGAACGTCGTGAGACAGTTCGGTCCATATCCGGTGTGG    6474
eGen1          AAGCGGTACGTGAGCTGGGTTCAGAACGTCGTGAGACAGTTCGGTCCATATCCGGTGTGG    6555
eGen2          AAGCGGTACGTGAGCTGGGTTCAGAACGTCGTGAGACAGTTCGGTCCATATCCGGTGTGG    6563
               ************************************************************

Potato         GCGTTAGAGCATTGAGAGGACCTTTCCCTAGTACGAGAGGACCGGGAAGGACGCACCTCT    6623
Tobacco        GCGTTAGAGCATTGAGAGGACCTTTCCCTAGTACGAGAGGACCGGGAAGGACGCACCTCT    6498
Gen1           GCGTTAGAGCATTGAGAGGACCTTTCCCTAGTACGAGAGGACCGGGAAGGACGCACCTCT    6534
eGen1          GCGTTAGAGCATTGAGAGGACCTTTCCCTAGTACGAGAGGACCGGGAAGGACGCACCTCT    6615
eGen2          GCGTTAGAGCATTGAGAGGACCTTTCCCTAGTACGAGAGGACCGGGAAGGACGCACCTCT    6623
               ************************************************************

Potato         GGTGTACCAGTTATCGTGCCCACGGTAAACGCTGGGTAGCCAAGTGCGGAGCGGATAACT    6683
Tobacco        GGTGTACCAGTTATCGTGCCCACGGTAAACGCTGGGTAGCCAAGTGCGGAGCGGATAACT    6558
Gen1           GGTGTACCAGTTATCGTGCCCACGGTAAACGCTGGGTAGCCAAGTGCGGAGCGGATAACT    6594
eGen1          GGTGTACCAGTTATCGTGCCCACGGTAAACGCTGGGTAGCCAAGTGCGGAGCGGATAACT    6675
eGen2          GGTGTACCAGTTATCGTGCCCACGGTAAACGCTGGGTAGCCAAGTGCGGAGCGGATAACT    6683
               ************************************************************

Potato         GCTGAAAGCATCTAAGTAGTAAGCCCACCCCAAGATGAGTGCTCTCCTATTCCGACTTCC    6743
Tobacco        GCTGAAAGCATCTAAGTAGTAAGCCCACCCCAAGATGAGTGCTCTCCTATTCCGACTTCC    6618
Gen1           GCTGAAAGCATCTAAGTAGTAAGCCCACCCCAAGATGAGTGCTCTCCTATTCCGACTTCC    6654
eGen1          GCTGAAAGCATCTAAGTAGTAAGCCCACCCCAAGATGAGTGCTCTCCTATTCCGACTTCC    6735
eGen2          GCTGAAAGCATCTAAGTAGTAAGCCCACCCCAAGATGAGTGCTCTCCTATTCCGACTTCC    6743
               ************************************************************
                      28
Potato         CCAGAGCTTCCGGTAGCACAGCCGAGACAGCGACGGGTTCTCTGCCCCTGCGGGGATGGA    6803
Tobacco        CCAGAGCCTCCGGTAGCACAGCCGAGACAGCGACGGGTTCTCTGCCCCTGCGGGGATGGA    6678
Gen1           CCAGAGCCTCCGGTAGCACAGCCGAGACAGCGACGGGTTCTCTGCCCCTGCGGGGATGGA    6714
eGen1          CCAGAGCTTCCGGTAGCACAGCCGAGACAGCGACGGGTTCTCTGCCCCTGCGGGGATGGA    6795
eGen2          CCAGAGCTTCCGGTAGCACAGCCGAGACAGCGACGGGTTCTCTGCCCCTGCGGGGATGGA    6803
               ******* ****************************************************
                                                             29
Potato         GCGACAGAAGTTTTTTTGAGAATTCAAGAGAAGGTCACGGCGAGACGAGCCGTTTATCAT    6863
Tobacco        GCGACAGAAGTTTTTTTGAGAATTCAAGAGAAGGTCACGGCGAGACGAGCCGTTTATCAT    6738
Gen1           GCGACAGAAGTTTTTTTGAGAATTCAAGAGAAGGTCACGGCGAGAGGAGCCGTTTATCAT    6774
eGen1          GCGACAGAAGTTTTTTTGAGAATTCAAGAGAAGGTCACGGCGAGACGAGCCGTTTATCAT    6855
eGen2          GCGACAGAAGTTTTTTTGAGAATTCAAGAGAAGGTCACGGCGAGACGAGCCGTTTATCAT    6863
               ********************************************* **************

Potato         TACGATAGGTGTCAAGTGGAAGTGCAGTGATGTATGCAGCTGAGGCATCCTAACAGACCG    6923
Tobacco        TACGATAGGTGTCAAGTGGAAGTGCAGTGATGTATGCAGCTGAGGCATCCTAACAGACCG    6798
Gen1           TACGATAGGTGTCAAGTGGAAGTGCAGTGATGTATGCAGCTGAGGCATCCTAACAGACCG    6834
```

```
eGen1      TACGATAGGTGTCAAGTGGAAGTGCAGTGATGTATGCAGCTGAGGCATCCTAACAGACCG   6915
eGen2      TACGATAGGTGTCAAGTGGAAGTGCAGTGATGTATGCAGCTGAGGCATCCTAACAGACCG   6923
           ************************************************************

Potato     GTAGACTTGAACCTTGTTCCTACATGACCTGATCAATTCGATCAGGCACTCGCCATCTAT   6983
Tobacco    GTAGACTTGAACCTTGTTCCTACATGACCTGATCAATTCGATCAGGCACTCGCCATCTAT   6858
Gen1       GTAGACTTGAACCTTGTTCCTACATGACCTGATCAATTCGATCAGGCACTCGCCATCTAT   6894
eGen1      GTAGACTTGAACCTTGTTCCTACATGACCTGATCAATTCGATCAGGCACTCGCCATCTAT   6975
eGen2      GTAGACTTGAACCTTGTTCCTACATGACCTGATCAATTCGATCAGGCACTCGCCATCTAT   6983
           ************************************************************

Potato     TTTCATTGTTCAAATCTTTGACAACACGAAAAAAACCATTGTTCAACTCTTTGACAACATG   7043
Tobacco    TTTCATTGTTCAAATCTTTGACAACACGAAAAAAACCATTGTTCAACTCTTTGACAACATG   6918
Gen1       TTTCATTGTTCAAATCTTTGACAACACGAAAAAAACCATTGTTCAACTCTTTGACAACATG   6954
eGen1      TTTCATTGTTCAAATCTTTGACAACACGAAAAAAACCATTGTTCAACTCTTTGACAACATG   7035
eGen2      TTTCATTGTTCAAATCTTTGACAACACGAAAAAAACCATTGTTCAACTCTTTGACAACATG   7043
           ************************************************************

Potato     AAAAAACCAAAAGCTCTGCCCTCCCTCTCTATCTATCCAAGGGATGGAAGGGCAGAGGCC   7103
Tobacco    AAAAAACCAAAAGCTCTGCCCTCCCTCTCTATCTATCCAAGGGATGGAAGGGCAGAGGCC   6978
Gen1       AAAAAACCAAAAGCTCTGCCCTCCCTCTCTATCTATCCAAGGGATGGAAGGGCAGAGGCC   7014
eGen1      AAAAAACCAAAAGCTCTGCCCTCCCTCTCTATCTATCCAAGGGATGGAAGGGCAGAGGCC   7095
eGen2      AAAAAACCAAAAGCTCTGCCCTCCCTCTCTATCTATCCAAGGGATGGAAGGGCAGAGGCC   7103
           ************************************************************

Potato     TTTGGTGTCCCCTCCAGTCAAGAATTGGGGCCTCACAATCACTAGCCAATATGCTTTTCT   7163
Tobacco    TTTGGTGTCCCCTCCAGTCAAGAATTGGGGCCTCACAATCACTAGCCAATATGCTTTTCT   7038
Gen1       TTTGGTGTCCCCTCCAGTCAAGAATTGGGGCCTCACAATCACTAGCCAATATGCTTTTCT   7074
eGen1      TTTGGTGTCCCCTCCAGTCAAGAATTGGGGCCTCACAATCACTAGCCAATATGCTTTTCT   7155
eGen2      TTTGGTGTCCCCTCCAGTCAAGAATTGGGGCCTCACAATCACTAGCCAATATGCTTTTCT   7163
           ************************************************************

Potato     CTCATGCCTTTCTTCGTTCATGGTTCGATATTCTGGTGTCCTAGGCGTAGAGGAACCACA   7223
Tobacco    CTCATGCCTTTCTTCGTTCATGGTTCGATATTCTGGTGTCCTAGGCGTAGAGGAACCACA   7098
Gen1       CTCATGCCTTTCTTCGTTCATGGTTCGATATTCTGGTGTCCTAGGCGTAGAGGAACCACA   7134
eGen1      CTCATGCCTTTCTTCGTTCATGGTTCGATATTCTGGTGTCCTAGGCGTAGAGGAACCACA   7215
eGen2      CTCATGCCTTTCTTCGTTCATGGTTCGATATTCTGGTGTCCTAGGCGTAGAGGAACCACA   7223
           ************************************************************

Potato     CCAATCCATCCCGAACTTGGTGGTTAAACTCTACTGCGGTGACGATACTGTAGGGGAGGT   7283
Tobacco    CCAATCCATCCCGAACTTGGTGGTTAAACTCTACTGCGGTGACGATACTGTAGGGGAGGT   7158
Gen1       CCAATCCATCCCGAACTTGGTGGTTAAACTCTACTGCGGTGACGATACTGTAGGGGAGGT   7194
eGen1      CCAATCCATCCCGAACTTGGTGGTTAAACTCTACTGCGGTGACGATACTGTAGGGGAGGT   7275
eGen2      CCAATCCATCCCGAACTTGGTGGTTAAACTCTACTGCGGTGACGATACTGTAGGGGAGGT   7283
           ************************************************************

Potato     CCTGCGGAAAAAATAGCTCGACGCCAGGATGATAAAAAGCTTAACACCTCTCATTCTTATT   7343
Tobacco    CCTGCGGAAAAAATAGCTCGACGCCAGGATGATAAAAAGCTTAACACCTCTCATTCTTATT   7218
Gen1       CCTGCGGAAAAAATAGCTCGACGCCAGGATGATAAAAAGCTTAACACCTCTCATTCTTATT   7254
eGen1      CCTGCGGAAAAAATAGCTCGACGCCAGGATGATAAAAAGCTTAACACCTCTCATTCTTATT   7335
eGen2      CCTGCGGAAAAAATAGCTCGACGCCAGGATGATAAAAAGCTTAACACCTCTCATTCTTATT   7343
           ************************************************************
                                       30            31
Potato     ACTTTTTCAATATGAAAACGAAAAAAA--AAAATGAAAAATAAAAAGGTCGTTTTATTCA   7401
Tobacco    ACTTTTTCAATATGAAAACGAAAAAAAAAAAAATGAAAAATCAAAAGGTCGTTTTATTCA   7278
Gen1       ACTTTTTCAATATGAAAACGAAAAAAAAAAAAATGAAAAATCAAAAGGTCGTTTTATTCA   7314
eGen1      ACTTTTTCAATATGAAAACGAAAAAAAAAAAAATGAAAAATCAAAAGGTCGTTTTATTCA   7395
eGen2      ACTTTTTCAATATGAAAACGAAAAAAA--AAAATGAAAAATAAAAAGGTCGTTTTATTCA   7401
           ***************************  ************.******************

Potato     AAACCCCAATTGTGACATCCCTTCTCTCCCACTTCACACCTCGGAACGCACCCTTCTTAT   7461
Tobacco    AAACCCCAATTGTGACATCCCTTCTCTCCCACTTCACACCTCGGAACGCACCCTTCTTAT   7338
Gen1       AAACCCCAATTGTGACATCCCTTCTCTCCCACTTCACACCTCGGAACGCACCCTTCTTAT   7374
```

```
eGen1       AAACCCCAATTGTGACATCCCTTCTCTCCCACTTCACACCTCGGAACGCACCCTTCTTAT   7455
eGen2       AAACCCCAATTGTGACATCCCTTCTCTCCCACTTCACACCTCGGAACGCACCCTTCTTAT   7461
            ************************************************************

            32                    33
Potato      AGACTTATAGAGATAAACGCGCTTTCACATCTTCTTAACCCGAAATGGCTGGGGAGAGGA   7521
Tobacco     --------AGAGATAAACGCGCCTTCACATCTTCTTAACCCGAAATGGCTGGGGAGAGGA   7390
Gen1        --------AGAGATAAACGCGCCTTCACATCTTCTTAACCCGAAATGGCTGGGGAGAGGA   7426
eGen1       --------AGAGATAAACGCGCCTTCACATCTTCTTAACCCGAAATGGCTGGGGAGAGGA   7507
eGen2       AGACTTATAGAGATAAACGCGCTTTCACATCTTCTTAACCCGAAATGGCTGGGGAGAGGA   7521
                    ************** *************************************

                                       34                  35
Potato      AAGGTTCCTTTTTTTGAGGGTACTCCCCGGAACAGATCCAGTGGAGACGGGGTGG        7576
Tobacco     AAGGTTCCTTTTTTTGAGGGTACTCCCGGGAACAGATCCAGTGGAGACGGGGTGG        7445
Gen1        AAGGTTCCTTTTTTTGAGGGTACTCCCGGGAACAGATCCAGTGGAGAGGGGGTGG        7481
eGen1       AAGGTTCCTTTTTTTGAGGGTACTCCCGGGAACAGATCCAGTGGAGAGGGGGTGG        7562
eGen2       AAGGTTCCTTTTTTTGAGGGTACTCCCCGGAACAGATCCAGTGGAGAGGGGGTGG        7576
            *************************** ******************* *******
```

Table S2: Multi sequence alignment of *trnI*/*trnA* region of potato plastome (*Solanum tuberosum;* GenBank: DQ386163.2) and Gen1 plasmid, along with the homologous region from the endogenous plastome of $_e$Gen1, $_e$Gen2$^{\Delta}$ and $_e$Gen2$_{1-3}$-containing lines. Mutation 8-14 are indicated whereas sequence specific of the Gen1 plasmids are indicated in bolt. Multi sequence alignment obtained using Clustal Omega Software (EMBL-EBI).

```
Potato          GCGCCTGACCCTGAGATGTGGATCATCCAAGGCACATTAGCATGGCGTACTCCTCCTGTT    60
Gen1            GCGCCTGACCCTGAGATGTGGATCATCCAAGGCACATTAGCATGGCGTACTCCTCCTGTT    60
eGen1           GCGCCTGACCCTGAGATGTGGATCATCCAAGGCACATTAGCATGGCGTACTCCTCCTGTT    60
eGen2Δ          GCGCCTGACCCTGAGATGTGGATCATCCAAGGCACATTAGCATGGCGTACTCCTCCTGTT    60
eGen21          GCGCCTGACCCTGAGATGTGGATCATCCAAGGCACATTAGCATGGCGTACTCCTCCTGTT    60
eGen22          GCGCCTGACCCTGAGATGTGGATCATCCAAGGCACATTAGCATGGCGTACTCCTCCTGTT    60
eGen23          GCGCCTGACCCTGAGATGTGGATCATCCAAGGCACATTAGCATGGCGTACTCCTCCTGTT    60
                ************************************************************

Potato          CGAACCGGGGTTTGAAACCAAACTCCTCCTCAGGAGGATAGATGGGGCGATTCGGGTGAG    120
Gen1            CGAACCGGGGTTTGAAACCAAACTCCTCCTCAGGAGGATAGATGGGGCGATTCGGGTGAG    120
eGen1           CGAACCGGGGTTTGAAACCAAACTCCTCCTCAGGAGGATAGATGGGGCGATTCGGGTGAG    120
eGen2Δ          CGAACCGGGGTTTGAAACCAAACTCCTCCTCAGGAGGATAGATGGGGCGATTCGGGTGAG    120
eGen21          CGAACCGGGGTTTGAAACCAAACTCCTCCTCAGGAGGATAGATGGGGCGATTCGGGTGAG    120
eGen22          CGAACCGGGGTTTGAAACCAAACTCCTCCTCAGGAGGATAGATGGGGCGATTCGGGTGAG    120
eGen23          CGAACCGGGGTTTGAAACCAAACTCCTCCTCAGGAGGATAGATGGGGCGATTCGGGTGAG    120
                ************************************************************

Potato          ATCCAATGTAGATCCAACTTTCGATTCACTCGTGGGATCCGGGCGGTCCGGGGGGGACCA    180
Gen1            ATCCAATGTAGATCCAACTTTCGATTCACTCGTGGGATCCGGGCGGTCCGGGGGGGACCA    180
eGen1           ATCCAATGTAGATCCAACTTTCGATTCACTCGTGGGATCCGGGCGGTCCGGGGGGGACCA    180
eGen2Δ          ATCCAATGTAGATCCAACTTTCGATTCACTCGTGGGATCCGGGCGGTCCGGGGGGGACCA    180
eGen21          ATCCAATGTAGATCCAACTTTCGATTCACTCGTGGGATCCGGGCGGTCCGGGGGGGACCA    180
eGen22          ATCCAATGTAGATCCAACTTTCGATTCACTCGTGGGATCCGGGCGGTCCGGGGGGGACCA    180
eGen23          ATCCAATGTAGATCCAACTTTCGATTCACTCGTGGGATCCGGGCGGTCCGGGGGGGACCA    180
                ************************************************************

Potato          CCACGGCTCCTCTCTTCTCGAGAATCCATACATCCCTTATCAGTGTATGGACAGCTATCT    240
Gen1            CCACGGCTCCTCTCTTCTCGAGAATCCATACATCCCTTATCAGTGTATGGACAGCTATCT    240
eGen1           CCACGGCTCCTCTCTTCTCGAGAATCCATACATCCCTTATCAGTGTATGGACAGCTATCT    240
eGen2Δ          CCACGGCTCCTCTCTTCTCGAGAATCCATACATCCCTTATCAGTGTATGGACAGCTATCT    240
eGen21          CCACGGCTCCTCTCTTCTCGAGAATCCATACATCCCTTATCAGTGTATGGACAGCTATCT    240
eGen22          CCACGGCTCCTCTCTTCTCGAGAATCCATACATCCCTTATCAGTGTATGGACAGCTATCT    240
eGen23          CCACGGCTCCTCTCTTCTCGAGAATCCATACATCCCTTATCAGTGTATGGACAGCTATCT    240
                ************************************************************
                                 8
Potato          CTCGAGCACAGGTTTAGGTTCGGCCTCAATGGGAAAATAAAATGGAGCACCTAACAACGC    300
Gen1            CTCGAGCACAGGTTTAGC---------AATGGGAAAATAAAATGGAGCACCTAACAACGC    291
eGen1           CTCGAGCACAGGTTTAGC---------AATGGGAAAATAAAATGGAGCACCTAACAACGC    291
eGen2Δ          CTCGAGCACAGGTTTAGC---------AATGGGAAAATAAAATGGAGCACCTAACAACGC    291
eGen21          CTCGAGCACAGGTTTAGGTTCGGCCTCAATGGGAAAATAAAATGGAGCACCTAACAACGC    300
eGen22          CTCGAGCACAGGTTTAGC---------AATGGGAAAATAAAATGGAGCACCTAACAACGC    291
eGen23          CTCGAGCACAGGTTTAGGTTCGGCCTCAATGGGAAAATAAAATGGAGCACCTAACAACGC    300
                *****************          *********************************
                                            9
Potato          ATCTTCACAGACCAAGAACTACGAGATCACCCCTTTCATTCTGGGGTGACGGAGGGATCG    360
Gen1            ATCTTCACAGACCAAGAACTACGAGATCGCCCCTTTCATTCTGGGGTGACGGAGGGATCG    351
eGen1           ATCTTCACAGACCAAGAACTACGAGATCGCCCCTTTCATTCTGGGGTGACGGAGGGATCG    351
eGen2Δ          ATCTTCACAGACCAAGAACTACGAGATCGCCCCTTTCATTCTGGGGTGACGGAGGGATCG    351
eGen21          ATCTTCACAGACCAAGAACTACGAGATCACCCCTTTCATTCTGGGGTGACGGAGGGATCG    360
eGen22          ATCTTCACAGACCAAGAACTACGAGATCGCCCCTTTCATTCTGGGGTGACGGAGGGATCG    351
eGen23          ATCTTCACAGACCAAGAACTACGAGATCACCCCTTTCATTCTGGGGTGACGGAGGGATCG    360
                **************************** *******************************
```

```
                                        10
Potato     TACCATTCGAGCCGTTTTTTTCTTGACTCGAAATCGAAATGGGAGCAGGTTTGAAAAAGG   420
Gen1       TACCATTCGAGCCGTTTTTTTCTTGACTCGAA------ATGGGAGCAGGTTTGAAAAAGG   405
eGen1      TACCATTCGAGCCGTTTTTTTCTTGACTCGAAATCGAAATGGGAGCAGGTTTGAAAAAGG   411
eGen2Δ     TACCATTCGAGCCGTTTTTTTCTTGACTCGAA------ATGGGAGCAGGTTTGAAAAAGG   405
eGen21     TACCATTCGAGCCGTTTTTTTCTTGACTCGAAATCGAAATGGGAGCAGGTTTGAAAAAGG   420
eGen22     TACCATTCGAGCCGTTTTTTTCTTGACTCGAA------ATGGGAGCAGGTTTGAAAAAGG   405
eGen23     TACCATTCGAGCCGTTTTTTTCTTGACTCGAAATCGAAATGGGAGCAGGTTTGAAAAAGG   420
           ********************************      **********************
                                       11
Potato     ATCTTAGAGTGTCTAGGGTTGGGCCAGGAGGGTCTCTTAACGCCTTCTTTTTTCTTCTCA   480
Gen1       ATCTTAGAGTGTCTAGGGTTGGGCCAGGAGGCTCTCTTAACGCCTTCTTTTTTCTTCTCA   465
eGen1      ATCTTAGAGTGTCTAGGGTTGGGCCAGGAGGCTCTCTTAACGCCTTCTTTTTTCTTCTCA   471
eGen2Δ     ATCTTAGAGTGTCTAGGGTTGGGCCAGGAGGCTCTCTTAACGCCTTCTTTTTTCTTCTCA   465
eGen21     ATCTTAGAGTGTCTAGGGTTGGGCCAGGAGGGTCTCTTAACGCCTTCTTTTTTCTTCTCA   480
eGen22     ATCTTAGAGTGTCTAGGGTTGGGCCAGGAGGCTCTCTTAACGCCTTCTTTTTTCTTCTCA   465
eGen23     ATCTTAGAGTGTCTAGGGTTGGGCCAGGAGGGTCTCTTAACGCCTTCTTTTTTCTTCTCA   480
           ******************************* ****************************

Potato     TCGGAGTTATTTCACAAAGACTTGCCAGGGTAAGGAAGAAGGGGGGAACAAGCACACTTG   540
Gen1       TCGGAGTTATTTCACAAAGACTTGCCAGGGTAAGGAAGAAGGGGGGAACAAGCACACTTG   525
eGen1      TCGGAGTTATTTCACAAAGACTTGCCAGGGTAAGGAAGAAGGGGGGAACAAGCACACTTG   531
eGen2Δ     TCGGAGTTATTTCACAAAGACTTGCCAGGGTAAGGAAGAAGGGGGGAACAAGCACACTTG   525
eGen21     TCGGAGTTATTTCACAAAGACTTGCCAGGGTAAGGAAGAAGGGGGGAACAAGCACACTTG   540
eGen22     TCGGAGTTATTTCACAAAGACTTGCCAGGGTAAGGAAGAAGGGGGGAACAAGCACACTTG   525
eGen23     TCGGAGTTATTTCACAAAGACTTGCCAGGGTAAGGAAGAAGGGGGGAACAAGCACACTTG   540
           ************************************************************

Potato     GAGAGCGCAGTACAACGGAGAGTTGTATGCTGCGTTCGGGAAGGATGAATCGCTCCCGAA   600
Gen1       GAGAGCGCAGTACAACGGAGAGTTGTATGCTGCGTTCGGGAAGGATGAATCGCTCCCGAA   585
eGen1      GAGAGCGCAGTACAACGGAGAGTTGTATGCTGCGTTCGGGAAGGATGAATCGCTCCCGAA   591
eGen2Δ     GAGAGCGCAGTACAACGGAGAGTTGTATGCTGCGTTCGGGAAGGATGAATCGCTCCCGAA   585
eGen21     GAGAGCGCAGTACAACGGAGAGTTGTATGCTGCGTTCGGGAAGGATGAATCGCTCCCGAA   600
eGen22     GAGAGCGCAGTACAACGGAGAGTTGTATGCTGCGTTCGGGAAGGATGAATCGCTCCCGAA   585
eGen23     GAGAGCGCAGTACAACGGAGAGTTGTATGCTGCGTTCGGGAAGGATGAATCGCTCCCGAA   600
           ************************************************************

Potato     AAGGAATCTATTGATTCTCTCCCAATTGGTTGGACCGTAGGTGCGATGATTTACTTCACG   660
Gen1       AAGGAATCTATTGATTCTCTCCCAATTGGTTGGACCGTAGGTGCGATGATTTACTTCACG   645
eGen1      AAGGAATCTATTGATTCTCTCCCAATTGGTTGGACCGTAGGTGCGATGATTTACTTCACG   651
eGen2Δ     AAGGAATCTATTGATTCTCTCCCAATTGGTTGGACCGTAGGTGCGATGATTTACTTCACG   645
eGen21     AAGGAATCTATTGATTCTCTCCCAATTGGTTGGACCGTAGGTGCGATGATTTACTTCACG   660
eGen22     AAGGAATCTATTGATTCTCTCCCAATTGGTTGGACCGTAGGTGCGATGATTTACTTCACG   645
eGen23     AAGGAATCTATTGATTCTCTCCCAATTGGTTGGACCGTAGGTGCGATGATTTACTTCACG   660
           ************************************************************
                12
Potato     GGCGAGGTCTCTGGTTCAAGTCCAGGATGGCCCAGCTGCGCCAGGGAAAAGAATAGAAGA   720
Gen1       GGCGAGCTCTCTGGTTCAAGTCCAGGATGGCCCAGCTGCGCCAGGGAAAAGAATAGAAGA   705
eGen1      GGCGAGGTCTCTGGTTCAAGTCCAGGATGGCCCAGCTGCGCCAGGGAAAAGAATAGAAGA   711
eGen2Δ     GGCGAGGTCTCTGGTTCAAGTCCAGGATGGCCCAGCTGCGCCAGGGAAAAGAATAGAAGA   705
eGen21     GGCGAGGTCTCTGGTTCAAGTCCAGGATGGCCCAGCTGCGCCAGGGAAAAGAATAGAAGA   720
eGen22     GGCGAGGTCTCTGGTTCAAGTCCAGGATGGCCCAGCTGCGCCAGGGAAAAGAATAGAAGA   705
eGen23     GGCGAGGTCTCTGGTTCAAGTCCAGGATGGCCCAGCTGCGCCAGGGAAAAGAATAGAAGA   720
           ****** *****************************************************
                 13
Potato     AGCATCTGAC------------------------------------TACTTCATGCATGC   744
Gen1       AGCATCTGGTTTAAACACTAGTCGACTCTAGATTAATTAACGCGCGCGCTTCATGCATGC   765
eGen1      AGCATCTGAC------------------------------------TACTTCATGCATGC   735
eGen2Δ     AGCATCTGAC------------------------------------TACTTCATGCATGC   729
eGen21     AGCATCTGAC------------------------------------TACTTCATGCATGC   744
eGen22     AGCATCTGAC------------------------------------TACTTCATGCATGC   729
eGen23     AGCATCTGAC------------------------------------TACTTCATGCATGC   744
           ********                                        ************
```

```
Potato    TCCACTTGGCTCGGGGGGATATAGCTCAGTTGGTAGAGCTCCGCTCTTGCAATTGGGTCG   804
Gen1      TCCACTTGGCTCGGGGGGATATAGCTCAGTTGGTAGAGCTCCGCTCTTGCAATTGGGTCG   825
eGen1     TCCACTTGGCTCGGGGGGATATAGCTCAGTTGGTAGAGCTCCGCTCTTGCAATTGGGTCG   795
eGen2Δ    TCCACTTGGCTCGGGGGGATATAGCTCAGTTGGTAGAGCTCCGCTCTTGCAATTGGGTCG   789
eGen21    TCCACTTGGCTCGGGGGGATATAGCTCAGTTGGTAGAGCTCCGCTCTTGCAATTGGGTCG   804
eGen22    TCCACTTGGCTCGGGGGGATATAGCTCAGTTGGTAGAGCTCCGCTCTTGCAATTGGGTCG   789
eGen23    TCCACTTGGCTCGGGGGGATATAGCTCAGTTGGTAGAGCTCCGCTCTTGCAATTGGGTCG   804
          ************************************************************

Potato    TTGCGATTACGGGTTGGATGTCTAATTGTCCAGGCGGTAATGATAGTATCTTGTACCTGA   864
Gen1      TTGCGATTACGGGTTGGATGTCTAATTGTCCAGGCGGTAATGATAGTATCTTGTACCTGA   885
eGen1     TTGCGATTACGGGTTGGATGTCTAATTGTCCAGGCGGTAATGATAGTATCTTGTACCTGA   855
eGen2Δ    TTGCGATTACGGGTTGGATGTCTAATTGTCCAGGCGGTAATGATAGTATCTTGTACCTGA   849
eGen21    TTGCGATTACGGGTTGGATGTCTAATTGTCCAGGCGGTAATGATAGTATCTTGTACCTGA   864
eGen22    TTGCGATTACGGGTTGGATGTCTAATTGTCCAGGCGGTAATGATAGTATCTTGTACCTGA   849
eGen23    TTGCGATTACGGGTTGGATGTCTAATTGTCCAGGCGGTAATGATAGTATCTTGTACCTGA   864
          ************************************************************

Potato    ACCGGTGGCTCACTTTTTCTAAGTAATGGGGAAGAGGACCGAAACGTGCCACTGAAAGAC   924
Gen1      ACCGGTGGCTCACTTTTTCTAAGTAATGGGGAAGAGGACCGAAACGTGCCACTGAAAGAC   945
eGen1     ACCGGTGGCTCACTTTTTCTAAGTAATGGGGAAGAGGACCGAAACGTGCCACTGAAAGAC   915
eGen2Δ    ACCGGTGGCTCACTTTTTCTAAGTAATGGGGAAGAGGACCGAAACGTGCCACTGAAAGAC   909
eGen21    ACCGGTGGCTCACTTTTTCTAAGTAATGGGGAAGAGGACCGAAACGTGCCACTGAAAGAC   924
eGen22    ACCGGTGGCTCACTTTTTCTAAGTAATGGGGAAGAGGACCGAAACGTGCCACTGAAAGAC   909
eGen23    ACCGGTGGCTCACTTTTTCTAAGTAATGGGGAAGAGGACCGAAACGTGCCACTGAAAGAC   924
          ************************************************************

Potato    TCTACTGAGACAAAGATGGGCTGTCAAGAACGTAGAGGAGGTAGGATGGGCAGTTGGTCA   984
Gen1      TCTACTGAGACAAAGATGGGCTGTCAAGAACGTAGAGGAGGTAGGATGGGCAGTTGGTCA   1005
eGen1     TCTACTGAGACAAAGATGGGCTGTCAAGAACGTAGAGGAGGTAGGATGGGCAGTTGGTCA   975
eGen2Δ    TCTACTGAGACAAAGATGGGCTGTCAAGAACGTAGAGGAGGTAGGATGGGCAGTTGGTCA   969
eGen21    TCTACTGAGACAAAGATGGGCTGTCAAGAACGTAGAGGAGGTAGGATGGGCAGTTGGTCA   984
eGen22    TCTACTGAGACAAAGATGGGCTGTCAAGAACGTAGAGGAGGTAGGATGGGCAGTTGGTCA   969
eGen23    TCTACTGAGACAAAGATGGGCTGTCAAGAACGTAGAGGAGGTAGGATGGGCAGTTGGTCA   984
          ************************************************************

Potato    GATCTAGTATGGATCGTACATGGACGGTAGTTGGAGTCGGCGGCTCTCCCAGGGTTCCCT   1044
Gen1      GATCTAGTATGGATCGTACATGGACGGTAGTTGGAGTCGGCGGCTCTCCCAGGGTTCCCT   1065
eGen1     GATCTAGTATGGATCGTACATGGACGGTAGTTGGAGTCGGCGGCTCTCCCAGGGTTCCCT   1035
eGen2Δ    GATCTAGTATGGATCGTACATGGACGGTAGTTGGAGTCGGCGGCTCTCCCAGGGTTCCCT   1029
eGen21    GATCTAGTATGGATCGTACATGGACGGTAGTTGGAGTCGGCGGCTCTCCCAGGGTTCCCT   1044
eGen22    GATCTAGTATGGATCGTACATGGACGGTAGTTGGAGTCGGCGGCTCTCCCAGGGTTCCCT   1029
eGen23    GATCTAGTATGGATCGTACATGGACGGTAGTTGGAGTCGGCGGCTCTCCCAGGGTTCCCT   1044
          ************************************************************

Potato    CATCTGAGATCTCTGGGGAAGAGGATCAAGTTGGCCCTTGCGAACAGCTTGATGCACTAT   1104
Gen1      CATCTGAGATCTCTGGGGAAGAGGATCAAGTTGGCCCTTGCGAACAGCTTGATGCACTAT   1125
eGen1     CATCTGAGATCTCTGGGGAAGAGGATCAAGTTGGCCCTTGCGAACAGCTTGATGCACTAT   1095
eGen2Δ    CATCTGAGATCTCTGGGGAAGAGGATCAAGTTGGCCCTTGCGAACAGCTTGATGCACTAT   1089
eGen21    CATCTGAGATCTCTGGGGAAGAGGATCAAGTTGGCCCTTGCGAACAGCTTGATGCACTAT   1104
eGen22    CATCTGAGATCTCTGGGGAAGAGGATCAAGTTGGCCCTTGCGAACAGCTTGATGCACTAT   1089
eGen23    CATCTGAGATCTCTGGGGAAGAGGATCAAGTTGGCCCTTGCGAACAGCTTGATGCACTAT   1104
          ************************************************************

Potato    CTCCCTTCAACCCTTTGAGCGAAATGCGGCAAAAGAAAAGGAAGGAAAATCCATGGACCG   1164
Gen1      CTCCCTTCAACCCTTTGAGCGAAATGCGGCAAAAGAAAAGGAAGGAAAATCCATGGACCG   1185
eGen1     CTCCCTTCAACCCTTTGAGCGAAATGCGGCAAAAGAAAAGGAAGGAAAATCCATGGACCG   1155
eGen2Δ    CTCCCTTCAACCCTTTGAGCGAAATGCGGCAAAAGAAAAGGAAGGAAAATCCATGGACCG   1149
eGen21    CTCCCTTCAACCCTTTGAGCGAAATGCGGCAAAAGAAAAGGAAGGAAAATCCATGGACCG   1164
eGen22    CTCCCTTCAACCCTTTGAGCGAAATGCGGCAAAAGAAAAGGAAGGAAAATCCATGGACCG   1149
eGen23    CTCCCTTCAACCCTTTGAGCGAAATGCGGCAAAAGAAAAGGAAGGAAAATCCATGGACCG   1164
          ************************************************************
```

```
Potato      ACCCCATCATCTCCACCCCGTAGGAACTACGAGATCACCCCAAGGACGCCTTCGGCATCC    1224
Gen1        ACCCCATCATCTCCACCCCGTAGGAACTACGAGATCACCCCAAGGACGCCTTCGGCATCC    1245
eGen1       ACCCCATCATCTCCACCCCGTAGGAACTACGAGATCACCCCAAGGACGCCTTCGGCATCC    1215
eGen2Δ      ACCCCATCATCTCCACCCCGTAGGAACTACGAGATCACCCCAAGGACGCCTTCGGCATCC    1209
eGen21      ACCCCATCATCTCCACCCCGTAGGAACTACGAGATCACCCCAAGGACGCCTTCGGCATCC    1224
eGen22      ACCCCATCATCTCCACCCCGTAGGAACTACGAGATCACCCCAAGGACGCCTTCGGCATCC    1209
eGen23      ACCCCATCATCTCCACCCCGTAGGAACTACGAGATCACCCCAAGGACGCCTTCGGCATCC    1224
            ************************************************************

Potato      AGGGGTCACGGACCGACCATAGAACCCTGTTCAATAAGTGGAACGCATTAGCTGTCCGCT    1284
Gen1        AGGGGTCACGGACCGACCATAGAACCCTGTTCAATAAGTGGAACGCATTAGCTGTCCGCT    1305
eGen1       AGGGGTCACGGACCGACCATAGAACCCTGTTCAATAAGTGGAACGCATTAGCTGTCCGCT    1275
eGen2Δ      AGGGGTCACGGACCGACCATAGAACCCTGTTCAATAAGTGGAACGCATTAGCTGTCCGCT    1269
eGen21      AGGGGTCACGGACCGACCATAGAACCCTGTTCAATAAGTGGAACGCATTAGCTGTCCGCT    1284
eGen22      AGGGGTCACGGACCGACCATAGAACCCTGTTCAATAAGTGGAACGCATTAGCTGTCCGCT    1269
eGen23      AGGGGTCACGGACCGACCATAGAACCCTGTTCAATAAGTGGAACGCATTAGCTGTCCGCT    1284
            ************************************************************
                                                         14
Potato      CTCAGGTTGGGCAGTCAGGGTCGGAGAAGGGCAATGACTCATTCTTAAAACCAGCGTTCT    1344
Gen1        CTCAGGTTGGGCAGTCAGGGTCGGAGAAGGGCAATGACTCATTC----------------    1349
eGen1       CTCAGGTTGGGCAGTCAGGGTCGGAGAAGGGCAATGACTCATTCTTAAAACCAGCGTTCT    1335
eGen2Δ      CTCAGGTTGGGCAGTCAGGGTCGGAGAAGGGCAATGACTCATTC----------------    1313
eGen21      CTCAGGTTGGGCAGTCAGGGTCGGAGAAGGGCAATGACTCATTCTTAAAACCAGCGTTCT    1344
eGen22      CTCAGGTTGGGCAGTCAGGGTCGGAGAAGGGCAATGACTCATTCTTAAAACCAGCGTTCT    1329
eGen23      CTCAGGTTGGGCAGTCAGGGTCGGAGAAGGGCAATGACTCATTCTTAAAACCAGCGTTCT    1344
            ********************************************

Potato      TAAGACCAAAGAGTCGGGCGGAAGGGGGGGAAAGCCCTCCGTTCCTGGTTCTCCTGTAGT    1404
Gen1        ------------------------------------------------------------    1349
eGen1       TAAGACCAAAGAGTCGGGCGGAAGGGGGGGAAAGCCCTCCGTTCCTGGTTCTCCTGTAGT    1395
eGen2Δ      ------------------------------------------------------------    1313
eGen21      TAAGACCAAAGAGTCGGGCGGAAGGGGGGGAAAGCCCTCCGTTCCTGGTTCTCCTGTAGT    1404
eGen22      TAAGACCAAAGAGTCGGGCGGAAGGGGGGGAAAGCCCTCCGTTCCTGGTTCTCCTGTAGT    1389
eGen23      TAAGACCAAAGAGTCGGGCGGAAGGGGGGGAAAGCCCTCCGTTCCTGGTTCTCCTGTAGT    1404


Potato      TGGATCCTCCGGAACCACAAGAATCCTTAGTTAGAATGGGATTCCAACTCAGCACCTTTT    1464
Gen1        --------------------------TTAGTTAGAATGGGATTCCAACTCAGCACCTTTT    1383
eGen1       TGGATCCTCCGGAACCACAAGAATCCTTAGTTAGAATGGGATTCCAACTCAGCACCTTTT    1455
eGen2Δ      --------------------------TTAGTTAGAATGGGATTCCAACTCAGCACCTTTT    1347
eGen21      TGGATCCTCCGGAACCACAAGAATCCTTAGTTAGAATGGGATTCCAACTCAGCACCTTTT    1464
eGen22      TGGATCCTCCGGAACCACAAGAATCCTTAGTTAGAATGGGATTCCAACTCAGCACCTTTT    1449
eGen23      TGGATCCTCCGGAACCACAAGAATCCTTAGTTAGAATGGGATTCCAACTCAGCACCTTTT    1464
                                      **********************************

Potato      GAGTGAGATTTTGAGAAGAGTTGCTCTTTGGAGAGCACAGTACGATGAAAGTTGTAAGCT    1524
Gen1        GAGTGAGATTTTGAGAAGAGTTGCTCTTTGGAGAGCACAGTACGATGAAAGTTGTAAGCT    1443
eGen1       GAGTGAGATTTTGAGAAGAGTTGCTCTTTGGAGAGCACAGTACGATGAAAGTTGTAAGCT    1515
eGen2Δ      GAGTGAGATTTTGAGAAGAGTTGCTCTTTGGAGAGCACAGTACGATGAAAGTTGTAAGCT    1407
eGen21      GAGTGAGATTTTGAGAAGAGTTGCTCTTTGGAGAGCACAGTACGATGAAAGTTGTAAGCT    1524
eGen22      GAGTGAGATTTTGAGAAGAGTTGCTCTTTGGAGAGCACAGTACGATGAAAGTTGTAAGCT    1509
eGen23      GAGTGAGATTTTGAGAAGAGTTGCTCTTTGGAGAGCACAGTACGATGAAAGTTGTAAGCT    1524
            ************************************************************

Potato      GTGTTCGGGGGGGAGTTATTGTCTATCGTTGGCCTCTATGGTAGAATCAGTCGGGGGACC    1584
Gen1        GTGTTCGGGGGGGAGTTATTGTCTATCGTTGGCCTCTATGGTAGAATCAGTCGGGGGACC    1503
eGen1       GTGTTCGGGGGGGAGTTATTGTCTATCGTTGGCCTCTATGGTAGAATCAGTCGGGGGACC    1575
eGen2Δ      GTGTTCGGGGGGGAGTTATTGTCTATCGTTGGCCTCTATGGTAGAATCAGTCGGGGGACC    1467
eGen21      GTGTTCGGGGGGGAGTTATTGTCTATCGTTGGCCTCTATGGTAGAATCAGTCGGGGGACC    1584
eGen22      GTGTTCGGGGGGGAGTTATTGTCTATCGTTGGCCTCTATGGTAGAATCAGTCGGGGGACC    1569
eGen23      GTGTTCGGGGGGGAGTTATTGTCTATCGTTGGCCTCTATGGTAGAATCAGTCGGGGGACC    1584
            ************************************************************
```

```
Potato     TGAGAGGCGGTGGTTTACCCTGCGGCGGATGTCAGCGGTTCGAGTCCGCTTATCTCCAAC    1644
Gen1       TGAGAGGCGGTGGTTTACCCTGCGGCGGATGTCAGCGGTTCGAGTCCGCTTATCTCCAAC    1563
eGen1      TGAGAGGCGGTGGTTTACCCTGCGGCGGATGTCAGCGGTTCGAGTCCGCTTATCTCCAAC    1635
eGen2Δ     TGAGAGGCGGTGGTTTACCCTGCGGCGGATGTCAGCGGTTCGAGTCCGCTTATCTCCAAC    1527
eGen21     TGAGAGGCGGTGGTTTACCCTGCGGCGGATGTCAGCGGTTCGAGTCCGCTTATCTCCAAC    1644
eGen22     TGAGAGGCGGTGGTTTACCCTGCGGCGGATGTCAGCGGTTCGAGTCCGCTTATCTCCAAC    1629
eGen23     TGAGAGGCGGTGGTTTACCCTGCGGCGGATGTCAGCGGTTCGAGTCCGCTTATCTCCAAC    1644
           ************************************************************

Potato     TCGTGAACTTAGCCGATACAA 1665
Gen1       TCGTGAACTTAGCCGATACAA 1584
eGen1      TCGTGAACTTAGCCGATACAA 1656
eGen2Δ     TCGTGAACTTAGCCGATACAA 1548
eGen21     TCGTGAACTTAGCCGATACAA 1665
eGen22     TCGTGAACTTAGCCGATACAA 1650
eGen23     TCGTGAACTTAGCCGATACAA 1665
           *********************
```

Supplementary Table S3: Primers used in this study. The primer id, the full name and the nucleotide sequence (from 5' to 3') are indicated in the table. The primers are subdivided in forward (1-23 Fw) and reverse (1-23 Rv) primers.

Primers forward

id	name	sequence (5'-3')
1 Fw	Selectio-Cassette-1-Fw	CAATGTGAGTTTTTGTAGTTGGATTTGCTCC
2 Fw	Cloning-Magic-PsiI-Fw	GACTCATTATAAAAACTGCCGAATTCGGAT CC
3 Fw	IR-frag-KasI-Fw	CTCATCGGCGCCTGACCCTGAGATGTGGATCA
4 Fw	trnA-Fw	CAGTAGAGTCTTTCAGTGGCACGTT
5 Fw	SSC2-Fw	CCCCCTAATATAAGACCCGACCC
6 Fw	mGFP-full-Fw	ATGAGTAAAGGAGAAGAACTTTTCAC
7 Fw	SmR-full-Fw	ATGGCAGAAGCGGTGATCGCCG
8 Fw	KanR-full-Fw	ATGATTGAACAGGATGGCCTG
9 Fw	SpcR-full-Fw	ATGCGTAGCCGTAATTGGA
10 Fw	rbcL-P-Fw	GCTGCCGAATCTTCTACTGG
11 Fw	IR3-full-left-Fw	TCTCCACTGGATCTGTTCCCGG
12 Fw	IR3-full-right-Fw	CAAACCTGCTCCCATTTCGAG
13 Fw	IR3-5'-ext-cas-Fw	GAAGGCGTCCTTGGGGTGAT
14 Fw	rbcL-q-Fw	AGATCTGCGAATCCCTGTTG
15 Fw	EF1-alpha-q-Fw	GATGGTCAGACACGTGAACA
16 Fw	KanR-q-Fw	CGGCAGAAAAAGTGAGCATT
17 Fw	Backbone-q-Fw	CTGGCGTAATAGCGAAGAGG
18 Fw	Actin-PoAc58-q1-Fw	GCTTCCCGATGGTCAAGTCA
19 Fw	SmR-q-Fw	TGAGGCGCTAAATGAAACCT
20 Fw	mGFP-q-Fw	TGGAAGCGTTCAACTAGCAG
21 Fw	IR-probe-Fw	GATATAGCTCAGTTGGTAGAGCTCCGCTCT
22 Fw	KanR-Probe-Fw	AACGCAATACGCTGGCTATCCG
23 Fw	aadA-Probe-Fw	ATCAGAGGTAGTTGGCGTCATCGAG

Primers reverse

id	name	sequence (5'-3')
1 Rv	Selectio-Cassette-1-Rv	CTGCAGCCCAAACAAATACAAAATCAAAATAGA
2 Rv	Cloning-Magic-PsiI-Rv	GACTCATTATAACATGTGCATCCTCTAGTAGCG
3 Rv	IR-frag-HindIII-Rv	ATCATAAAGCTTTGTATCGGCTAAGTTCACGAG
4 Rv	trnI-Rv	GCCAGGGTAAGGAAGAAGGGG
5 Rv	SSC2-Rv	CCGAATTACGAAGGCTTAGTTCGG
6 Rv	mGFP-full-Rv	TTATTTGTATAGTTCATCCATGCCATG
7 Rv	SmR-full-Rv	TTATTTGCCGACTACCTTGGTGATC
8 Rv	KanR-full-Rv	TTAGAAAAATTCATCCAGCAGAC
9 Rv	SpcR-full-Rv	TTATTTACCCACCACTTTGGTAA
10 Rv	rbcL-P-Rv	CAGGGCTTTGAACCCAAATA
11 Rv	IR3-full-left-Rv	CATGGACGGTAGTTGGAGTCG

12 Rv	IR3-full-right-Rv	GTGGAACAGAATTGACTGGGTGGT
13 Rv	IR3-3'-ext-cas-Rv	TCTCTCGAGCACAGGTTTAGCA
14 Rv	rbcL-q-Rv	CAGGGGACGACCATACTTGT
15 RV	EF1-alpha-q-Rv	CCTTGGAGTACTTGGGGGTG
16 Rv	KanR-q-Rv	CGCACGTTCAATACGATGTT
17 Rv	Backbone-q-Rv	ACCGAGATAGGGTTGAGTGG
18 Rv	Actin-PoAc58-q1-Rv	GGATTCCAGCTGCTTCCATTC
19 Rv	SmR-q-Rv	TACTGCGCTGTACCAAATGC
20 Rv	mGFP-q-Rv	AAAGGGCAGATTGTGTGGAC
21 Rv	IR-probe-Rv	GCGGACAGCTAATGCGTTCCACTTATTGAA
22 Rv	KanR-Probe-Rv	TGCTGGATGTGGTGACCGAAGC
23 Rv	aadA-Probe-Rv	AAGCACTACATTTCGCTCATCGCCA
